# Detection and correction of underassigned rotational symmetry prior to structure deposition

**DOI:** 10.1107/S0907444910001502

**Published:** 2010-04-21

**Authors:** Billy K. Poon, Ralf W. Grosse-Kunstleve, Peter H. Zwart, Nicholas K. Sauter

**Affiliations:** a Physical Biosciences Division, Lawrence Berkeley National Laboratory, One Cyclotron Road, Berkeley, CA 94720, USA

**Keywords:** underassigned rotational symmetry, *LABELIT*, validation

## Abstract

Up to 2% of X-ray structures in the Protein Data Bank (PDB) potentially fit into a higher symmetry space group. Redundant protein chains in these structures can be made compatible with exact crystallographic symmetry with minimal atomic movements that are smaller than the expected range of coordinate uncertainty. The incidence of problem cases is somewhat difficult to define precisely, as there is no clear line between underassigned symmetry, in which the subunit differences are unsupported by the data, and pseudosymmetry, in which the subunit differences rest on small but significant intensity differences in the diffraction pattern. To help catch symmetry-assignment problems in the future, it is useful to add a validation step that operates on the refined coordinates just prior to structure deposition. If redundant symmetry-related chains can be removed at this stage, the resulting model (in a higher symmetry space group) can readily serve as an isomorphous replacement starting point for re-refinement using re-indexed and re-integrated raw data. These ideas are implemented in new software tools available at http://cci.lbl.gov/labelit.

## Abstract 2

An X-ray structural model can be reassigned to a higher symmetry space group using the presented framework if its noncrystallographic symmetry operators are close to being exact crystallographic relationships. About 2% of structures in the Protein Data Bank can be reclassified in this way.

## 1. Introduction

The accuracy of the molecular model derived from X-ray crystallography is inherently limited by measurement uncertainty in the structure factors and intrinsic disorder of the crystal. Indeed, atomic level accuracy is only possible if the data-set resolution approaches or exceeds 1.0 Å (see Afonine *et al.*, 2007 ▶, and references therein). At lower resolutions, prior assumptions about the stereochemistry are required in order to sufficiently restrain the refinement process (Hendrickson, 1985 ▶). Likewise, restraints arising from noncrystallographic sym­metry (NCS) averaging are important for shaping the molecular envelope and producing interpretable electron-density maps (Jones & Liljas, 1984 ▶). However, in view of the probabilistic nature of these restraints it is best to exploit true constraints such as crystallographic symmetry when they are available. Symmetry constraints have two benefits: merging of the symmetry-equivalent reflections increases the accuracy of the measured structure factors and modeling the asymmetric unit rather than the entire unit cell markedly decreases the number of parameters in the molecular model. A failure to identify the highest space-group symmetry compatible with the observations can have severe consequences for model building (Kleywegt *et al.*, 1996 ▶), leading to unwarranted con­clusions about the biology of the system under study.

This paper deals with the issue of finding potentially higher crystallographic symmetry given a particular data set and model. While the choice of space group is a routine aspect of structure solution, it is worth keeping in mind that experimental measurements never establish the space group with absolute confidence. There are always physical uncertainties to be considered both in the positions and the intensities of the Bragg reflections. Uncertainties in Bragg spot position affect the first step of space-group assignment, in which the crystal is classified into one of 14 Bravais types (based on the metric symmetry of the unit-cell dimensions). Starting with the three lengths and three angles of the unit cell, a convenient way to evaluate a potential symmetry axis is to compute the δ angle between the axis vectors expressed in direct and reciprocal space (Le Page, 1982 ▶). If the δ angle is identically zero the axis qualifies as a rotational symmetry operator as far as the unit-cell measurements are concerned. However, practical experience with typical rotation photography experiments shows that an allowance must be made for deviations as high as 1.4° from perfect alignment in order to construct the highest symmetry Bravais type consistent with the data (Sauter *et al.*, 2004 ▶, 2006 ▶).

Beyond the classification of Bravais type, measurement uncertainties in the Bragg intensities can potentially hinder the assignment of the diffraction’s symmetry. Here again it is possible to evaluate individual symmetry operators based on the agreement of symmetry-related intensity measurements. (Friedel mates are treated as equivalent throughout this paper, regardless of whether there is an anomalous scattering signal.) Defining the symmetry-operator reliability *R*_symop_ as the average percentage difference between pairs of symmetry-related intensity measurements (equation 2 in Sauter *et al.*, 2006 ▶), this statistic is ideally zero for a valid symmetry operation. However, nonzero values of up to 25% must be permitted (to account for poor measurement and/or anomalous signals) in order to assemble an optimal set of operators to describe the diffraction symmetry (Sauter *et al.*, 2006 ▶; Evans, 2006 ▶).

It would be desirable if the acceptable tolerances chosen for δ and *R*_symop_ could always be large enough to reflect the physical uncertainties for the specific experiment, but there is no established method to make this guarantee. Either by intention or by mistake structures can be solved in space groups with symmetries that are too low. Indeed, from time to time it has been remarked (Hooft *et al.*, 1994 ▶, 1996 ▶; Zwart *et al.*, 2008 ▶) that certain structures deposited in the PDB (Berman *et al.*, 2003 ▶) appear to have redundant subunit chains that are related by unassigned rotational symmetry operators. Furthermore, we have observed that some commonly used methods to determine the Bravais lattice are susceptible to numerical instability (Grosse-Kunstleve *et al.*, 2004 ▶; Sauter *et al.*, 2004 ▶), making it possible for high-symmetry Bravais types to be improperly identified, such as hexagonal rhombohedral (hR) being assigned as *C*-centered monoclinic (mC).

For small-molecule crystal structures, cases requiring re­assignment into a higher space group have been well documented (Marsh & Herbstein, 1988 ▶; Marsh, 1995 ▶, 1997 ▶, 2009 ▶; Marsh & Spek, 2001 ▶) and symmetry-validation software is available (Le Page, 1988 ▶; Palatinus & van der Lee, 2008 ▶; Spek, 2009 ▶). Here, we perform a similar function for the macromolecular field, surveying the entire PDB for underassigned rotational symmetry operators. [We address neither underassigned translational symmetry operators, as was performed recently by Zwart *et al.* (2005 ▶, 2008 ▶), nor the topic of merohedral twinning, as has been covered by Lebedev *et al.* (2006) ▶.] Since we do not usually have recourse to the original raw data images, no judgements are made about the true crystallo­graphic symmetry in individual cases. Rather, we develop scoring tools to quantify how closely a particular atomic model appears to fit into a higher symmetry, and coordinate-manipulation tools to interconvert models between space groups. The tools are intended to be used by the original investigator for validating the model at any stage prior to structure deposition or for correcting a model that is deemed suitable for re-analysis in a higher symmetry.

## 2. Computational methods

Software development was greatly facilitated by the framework provided by the open-source *Computational Crystallo­graphy Toolbox* (*cctbx*; Grosse-Kunstleve *et al.*, 2002 ▶, 2006 ▶). PDB coordinate files from http://wwpdb.org were parsed with the *cctbx.iotbx.pdb* file reader. Analysis was restricted to co­ordinate sets determined by X-ray crystallography and additionally to proteins rather than oligonucleotides. Solvent molecules, ligands, covalent modifications and alternate conformations were ignored. Structure factors from the PDB, when available, were validated with *phenix.cif_as_mtz* (Urzhumtseva *et al.*, 2009 ▶) to assure consistency with the corresponding PDB coordinate entry. Raw diffraction images for selected cases were downloaded from the Joint Center for Structural Genomics (JCSG; http://www.jcsg.org).

### 2.1. Automated structure solution in all possible subgroups

Before proceeding with the all-PDB survey, we wish to confirm that the true symmetry can be deduced from the atomic model if the structure is intentionally solved in a lower symmetry space group. Such structures were generated automatically using original JCSG data sets as a starting point and are illustrated here using PDB entry 3b77 (Table S2^1^ gives further examples). After integrating the 3b77 data set in the triclinic setting, merging trials performed with *labelit.rsymop* (Sauter *et al.*, 2006 ▶) show that the Bragg intensities, together with the unit-cell dimensions, are consistent with Patterson symmetries *P*4/*m, P*12/*m*1 or 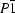. To obtain structure solutions in all three possible symmetries, the data were re-integrated, scaled and merged separately in each of these settings. Molecular-replacement solutions were determined with the program *phenix.automr* (McCoy *et al.*, 2007 ▶) using the published *P*4 structure as a replacement model. Solutions *A*1, *A*2 and *A*3 (corresponding to the three symmetries noted above) were then built and refined with *phenix.autobuild* (Terwilliger *et al.*, 2008 ▶). As this particular data set consists of a 90° rotation wedge intended for a tetragonal structure, the completeness of the data is quite low (57% out to a limiting resolution of 3.5 Å) when pro­cessed in the triclinic setting; however, this is still sufficient for the present purpose. To afford a comparison between crystallographic *R* factors (Table 1 ▶), each structure is refined at the same resolution and the same set of free-*R* flags as initially calculated for highest symmetry space group (*P*4) is expanded into the monoclinic and tri­clinic settings.

### 2.2. Relating the input symmetry to potential higher symmetries

In principle, it should be straightforward to check whether an atomic model can be reassigned to a higher symmetry target space group **G**. One simply lists the symmetry operators of the target space group and selects the operators that are absent in the input space group **H**. Applying these trial operators to the input structure will leave both atomic coordinates and structure-factor intensities invariant if the target symmetry is valid.

**Figure 1:**
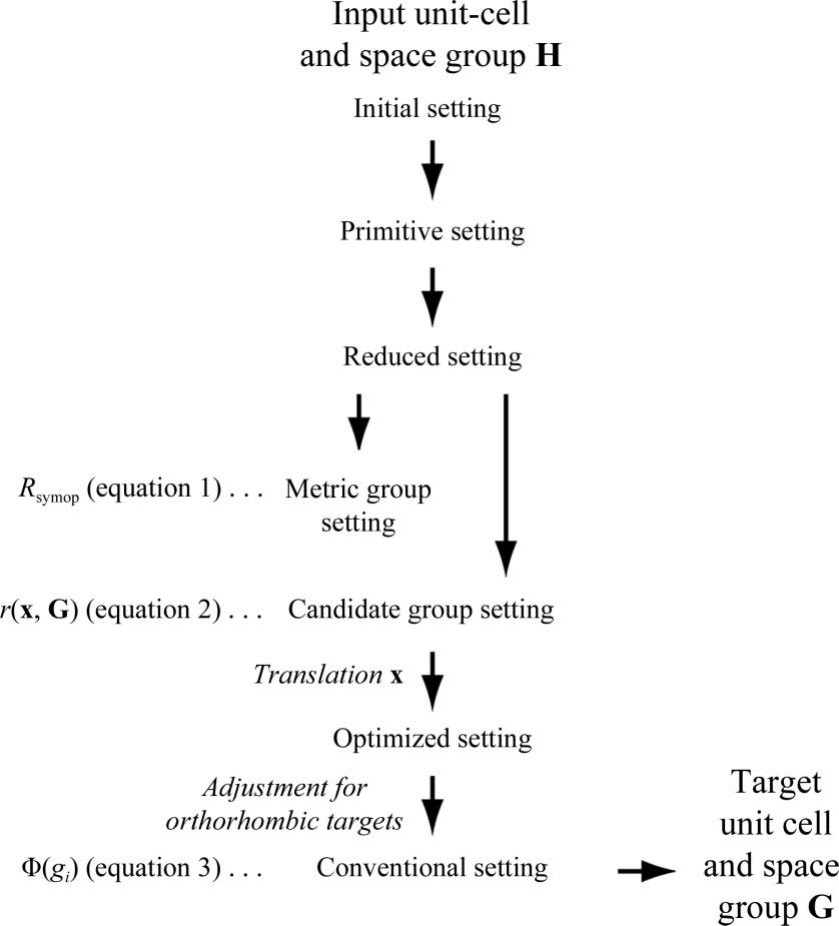
Reference-frame manipulations required before an input structure can be evaluated for fit into a higher-symmetry target space group. All the vertical arrows (except for the one labeled ‘translation **x**’) represent change of basis operators consisting of a rotation matrix and a translation vector, each containing rational values (ratios of small whole numbers). The operators can be composed together to form an overall operator (**R**, **T**), for example, transforming the input structure into a setting that is consistent with the reference frame of the candidate symmetry. An additional real-valued translation **x** optimizes the position of the input model with respect to the symmetry axes of the target space group. The reference frames used for evaluating equations (1)–(3) [Disp-formula fd2] [Disp-formula fd3] are indicated. Importantly, the final ‘conventional setting’ structure is still exactly superimposable with the input structure. The imposition of target symmetry constraints is a separate operation (horizontal arrow) and is discussed in §[Sec sec2.6]2.6.

In practice this calculation is fairly complicated since space groups are conventionally expressed in different reference frames (Hahn, 1996 ▶). In the general case, the input and target symmetries will have different unit-cell basis vectors **a**, **b**, **c** and choices of origin. To assure that **H** is a subgroup of **G** a single point of view must be chosen, and the approach taken here is to perform all comparisons in the reference frame of the target symmetry. Converting from the input to the target reference frame requires the sequence of transformations depicted in Fig. 1 ▶. Beginning with the initial setting, a change of basis (Boisen & Gibbs, 1990 ▶) is applied to remove any centering operations (Grosse-Kunstleve, 1999 ▶). This primitive cell is then changed to a standard reduced setting (the ‘minimum’ setting defined in Grosse-Kunstleve *et al.*, 2004 ▶). To afford comparisons between Bragg reflections that are potentially symmetry-equivalent, we enumerate all Patterson settings that align with the cell to within a tolerance δ (Sauter *et al.*, 2006 ▶) and change the basis to each of these metric group settings in turn. Having selected one of these metric settings (see §[Sec sec2.3]2.3), we then need to evaluate all of the candidate space groups that share the same Patterson symmetry as the metric group, each requiring a basis change from the reduced setting to the candidate setting. At this point, a fractional translation (see §[Sec sec2.4]2.4) must be applied so that duplicate polypeptide chains are correctly related by the the candidate space group’s rotational symmetry operators. A final adjustment to the conventional setting is necessary in certain cases, particularly those orthorhombic cases in which the target symmetry is in a nonstandard setting such as *P*2_1_22, which must be con­verted to the standard setting (*P*222_1_) by an axis swap.

Each transformation in this sequence is represented by a change-of-basis operator, which combines a rotation matrix and a translation vector, each containing rational-valued elements. These operators are mathematically associative, so that the total transformation from input to target setting is succinctly expressed as a single rotation **R** and translation **T**. As detailed elsewhere (Giacovazzo *et al.*, 1992 ▶; Sauter *et al.*, 2006 ▶), the transformation (**R**, **T**) can be applied to fractional coordinates, Miller indices and symmetry operations from the input structure in order to re-express them in the target reference frame. It is important to realise that the entire input structure is moved as a rigid body under the operation (**R**, **T**), so the symmetry properties of the structure do not change during the transformation. It is just a matter of convenience to move the structure into the same reference frame where we already have a list of the trial symmetry operators of the target space group.

### 2.3. Evaluation of the Patterson symmetry

We expected the possibility that the models from §2.1 solved in suboptimal space groups (*A*2 and *A*3) would have poorer crystallographic *R* factors than the optimal model *A*1. Instead, we found that the *R*-factor statistic did not help at all to distinguish between the best symmetry and the underassigned symmetry. The implication is one of caution: if the optimal Patterson symmetry is passed over at the stage of indexing and integration then the model-building and refinement process may be completed successfully without any indication of the oversight.

Fortunately, the model itself can be examined (following the approach of §[Sec sec2.2]2.2) to assess its compatibility with higher symmetry. A first step (Tables 2 ▶ and 3 ▶) is to establish missing symmetry operators based on back-calculated reflection intensities, *I* ^calc^. After expanding the atomic coordinate model to space group *P*1, the unit-cell measurements are used to construct the largest possible set of lattice symmetry operators, as described previously (Sauter *et al.*, 2006 ▶). Each potential operator **W** is then independently scored based on the agreement of symmetry-related intensities, 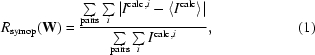
where 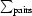 is a sum over all pairs of Bragg spots related by **W** and 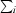 is a sum over both members of the pair. Low *R*_symop_ values indicate valid rotational symmetry in reciprocal space and in the illustrated example it is apparent that there is a fourfold rotation along the *z* axis (Table 2 ▶). The fourfold is equally clear regardless of whether the model is taken from the monoclinic or the triclinic structure. The triclinic structure (*A*3) additionally reveals a twofold symmetry along the *z* axis, while the monoclinic model (*A*2) already assumes the pre­sence of this twofold, so the *R*_symop_ value for this operator is zero.

In Table 3 ▶ the lattice symmetry operators are grouped together to show all possible Patterson settings consistent with the unit cell (to within the small angular tolerance δ). Each setting is scored by tabulating the worst-case symmetry-equivalence measure (*R*_symop_), considering all operators in the group. As expected, the illustrated example (triclinic structure *A*3) is consistent with only three of the metrically possible Patterson settings, namely *P*4/*m*, *P*12/*m*1 and 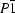, and not with any groups containing a twofold in the *xy* plane.

We arrive at the same conclusions about symmetry if we use the experimentally observed data (Tables 2 ▶ and 3 ▶) rather than model-calculated intensities. Starting with merged structure-factor amplitudes |*F* ^obs^|, the observations are expanded to *P*1, re-expressed as reflection intensities (*I* ^obs^) and used in (1) instead of *I* ^calc^. This methodology is readily used to evaluate the potential Patterson settings in any deposited reflection file from the PDB.

### 2.4. Identification of the space group and positioning of the model

Symmetry-equivalence of the reflections (1), together with knowledge of the unit cell, establishes the highest possible Patterson symmetry, but two questions remain to be answered: what is the space group and where should the model be placed in the higher symmetry unit cell? Taking the example of structure *A*3, we wish to know which of the tetragonal space groups to focus on (*P*4, *P*4_1_, *P*4_2_ or *P*4_3_) and where to place the polypeptide in relation to the *z* axis.

We begin by defining **x**, the fractional origin shift that must be applied in the setting of the target space group **G** to the input model in order to properly position it within the higher symmetry unit cell (denoted as ‘translation **x**’ in Fig. 1 ▶). The model is correctly positioned when the application of space-group symmetry operators leaves the model invariant. In view of the prohibitive computational cost of translating the model to every position in the unit cell, we adopt a method from Navaza & Vernoslova (1995 ▶), dramatically speeding up the calculation by gauging the correlation between two types of calculated Bragg intensity: *I* ^merge,**G**^ and *I* ^ensemble,**G**^(**x**). *I* ^merge,**G**^ is simply the set of reflection intensities calculated by expanding the atomic coordinates of the present model into space group *P*1 and merging the symmetry equivalents under space group **G**. *I* ^ensemble,**G**^(**x**) is the result of applying the origin shift **x**, thus repositioning the model in the unit cell. The symmetry elements of **G** are then applied, giving a hypothetical ensemble containing multiple copies of the *P*1 model superimposed upon each other (one copy for each symmetry operator) from which intensities *I* ^ensemble,**G**^(**x**) are calculated. The agreement between present model, origin shift and space group is described by the Pearson correlation coefficient
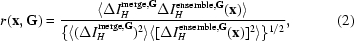
where 〈 〉 is the average over all Miller indices *H* and Δ*I_H_* = *I_H_* − 〈*I*〉 is the deviation between the calculated intensity for a given Miller index and the average over all intensities. Navaza and Vernoslova’s Fast Fourier approach for calculating *r*(**x**, **G**) is computationally tractable even for large structures.

**Figure 2:**
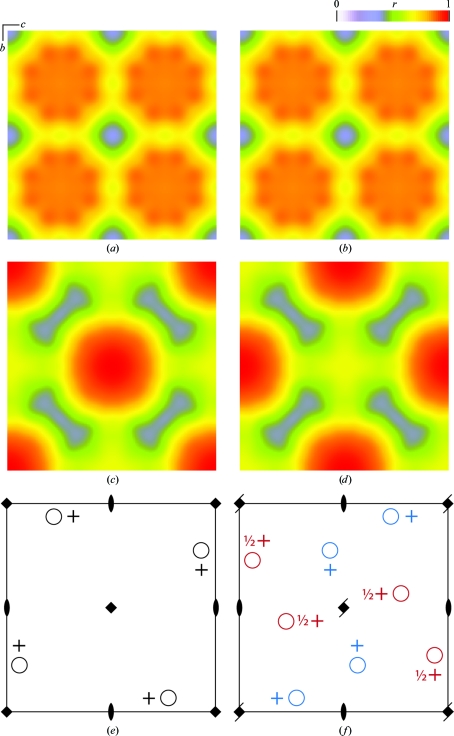
Correlation *r*(**x**, **G**) between model intensities from structure *A*3 and intensities from an ensemble to which symmetry operators from four space groups have been applied: *P*4_1_ (*a*), *P*4_3_ (*b*), *P*4 (*c*) and *P*4_2_ (*d*). For (*a*)–(*d*), the illustrated sections represent one unit cell sliced perpendicular to the *a* axis, which is the noncrystallographic fourfold symmetry axis of this triclinic structure. In space group *P*4 (*e*), the ensemble structure correlates nearly exactly with the triclinic model (both depicted as black atoms), reflecting the origin-shift peak at **x**_max_ = **0** in (*c*). For space group *P*4_2_ (*f*), in contrast, the application of the origin shift **x**_max_ = ½**c** gives a triclinic model (blue atoms) that is different from the ensemble structure (blue + red atoms together), yet the calculated intensities from the blue and red models are identical. This explains why the peaks in (*c*) and (*d*) are both approximately equal to 1.0. Symmetry-operator symbols are as defined in *International Tables for Crystallography* (Hahn, 1996 ▶).

Peaks in the *r*(**x**, **G**) map that approach a value of 1.0 represent candidate translations for positioning the model into the target unit cell. In the illustrated example (Figs. 2 ▶ *a*–2 ▶ *d*), the relatively low correlation coefficients under *P*4_1_ and *P*4_3_ allow us to rule out these space groups, while space groups *P*4 and *P*4_2_ are both shown to be viable candidates as far as intensity correlations are concerned. When viewing these correlation maps it is useful to realise that the *r*(**x**, **G**) function has a special type of symmetry variously called the Cheshire group (Hirshfeld, 1968 ▶) or affine normalizer (Koch & Fischer, 1996 ▶); the effect of this is to restrict the range of possible origin shifts to an area or volume smaller than the unit cell of **G**. For the four tetragonal space groups under consideration *r*(**x**, **G**) is independent of the position along the fourfold, so it is only necessary to illustrate a single section in Figs. 2 ▶(*a*)–2 ▶(*d*).

The correlation coefficient of (2) is very efficient for discriminating among origin shifts, but in this case it does not distinguish between the two candidate models that might be consistent with structure *A*3: a *P*4 model with origin shift **x**_max_ = **0** (Fig. 2 ▶ *e*) and a *P*4_2_ model shifted by **x**_max_ = ½**c** (Fig. 2 ▶ *f*). The latter model happens to be incorrect in the sense that application of the 4_2_ screw leads to an atomic model (red circles in Fig. 2 ▶ *f*) that sterically clashes with the starting model (blue circles) rather than aligning with it; each asymmetric unit is effectively duplicated. Yet the calculated intensities for the two sets of asymmetric units are identical since intensities are invariant under the screw axis operator. What is missing in (2) is a recognition that the screw operation affects the structure-factor phase, even though it does not affect the amplitude.

Properly accounting for phases requires a separate calculation. We take the input model (triclinic structure *A*3 in this case), apply the origin shift **x**_max_ determined above, and then consider the calculated structure factors *F* ^calc^ and phases ϕ^calc^. Looking separately at each symmetry operator *g*_*i*_ of space group  **G**, a weighted phase difference factor is used to construct a symmetry agreement score as suggested by Palatinus & van der Lee (2008 ▶),
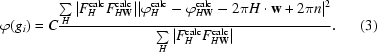
In this expression, symmetry operator *g*_*i*_ has a rotational part **W** and a translational part **w**. The normalization constant *C* and modular integer *n* are as described in Palatinus & van der Lee (2008 ▶). Models that are invariant under the symmetry operation will have equal values of ϕ_*H*_^calc^ and ϕ_*H***W**_^calc^ + 2π*H*·**w**, so the score will be zero. In our example, the symmetry agreement scores ϕ(**4**) = 0.002 and ϕ(**4**_2_) = 0.578 clearly establish the correct space group as *P*4.

### 2.5. Positional refinement of the higher symmetry model

The Pearson correlation coefficient (2) is evaluated on a grid whose granularity is approximately half the limiting resolution of the diffraction. Therefore, the origin shift **x**_max_ from §[Sec sec2.4]2.4 is only a first approximation. Indeed, the displacement between the atomic model and the symmetry axes of the unit cell should arguably be the most precise element of any structure. Since the displacement is derived jointly from the positions of all the atoms, its uncertainty should be a tiny fraction of a bond length. It is thus appropriate to subject the origin shift to additional refinement. Furthermore, while (3) scores the symmetry agreement of structure factors in reciprocal space, it is also desirable to quantify the symmetry based on the atomic model in direct space (or even to provide a computer-graphics snapshot of superimposed symmetry-equivalent molecules), giving a better intuitive grasp of the symmetry fit. This section presents methods for addressing these issues.

#### 2.5.1. Matching of symmetry-equivalent molecules aided by coset decomposition

As noted in §[Sec sec2.2]2.2, we judge a target space group **G** by applying symmetry operators present in **G** that are absent in the input space group  **H**. The relationship between group **G** and its subgroup **H** can be most usefully explored by the decomposition tools of group theory. In particular, the left coset decomposition of  **G** with respect to **H** is defined as
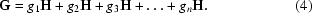
In this expansion, **G** is broken down into a series of *n* subsets (left cosets) generated by applying the symmetry operators *g*_*i*_ ∈ **G** to each element of **H**. Operator *g*_1_ is defined to be the identity, while the elements *g*_2_…*g*_*n*_, termed left coset representatives, are the elements that require evaluation as trial sym­metry operators for the crystal structure. The choice of which elements to count as left coset representatives is not unique; within each left coset any element can be chosen as the representative with equivalent results. The important property here is that only one representative from each coset need be considered.

The coset expansion makes it possible to quantify how close the non­crystallographic symmetry relationships of a structure come to crystallo­graphic exactness. A necessary first step is to derive trial mappings of the asymmetric unit contents to itself, one mapping for each coset. The algorithm begins by origin-shifting the input structure to the optimized setting (Fig. 1 ▶). Matching polypeptide pairs (*X* to *Y*) are then determined for each coset representative *g*_*i*_ using a triple loop. In the outer loop, *g*_*i*_ is applied to each polypeptide chain *X* of the asymmetric unit. In the middle loop, each polypeptide chain *Y* is evaluated as a matching target (with the requirement that *Y* is only considered as a candidate if *X* and *Y* have similar amino-acid sequences). In the innermost loop, each operator *h* ∈ **H** is applied to *Y* and a match is declared if the coordinates approximately superimpose,
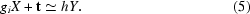
In this expression, the atomic coordinates of polypeptides *X* and *Y* are expressed in fractional coordinates and **t** represents an allowable translation vector on the lattice (one containing full-integer components). Superposition is determined using the method of Kearsley (1989 ▶) and calculations throughout this paper are limited to the C^α^ atoms of polypeptide chains.

**Figure 3:**
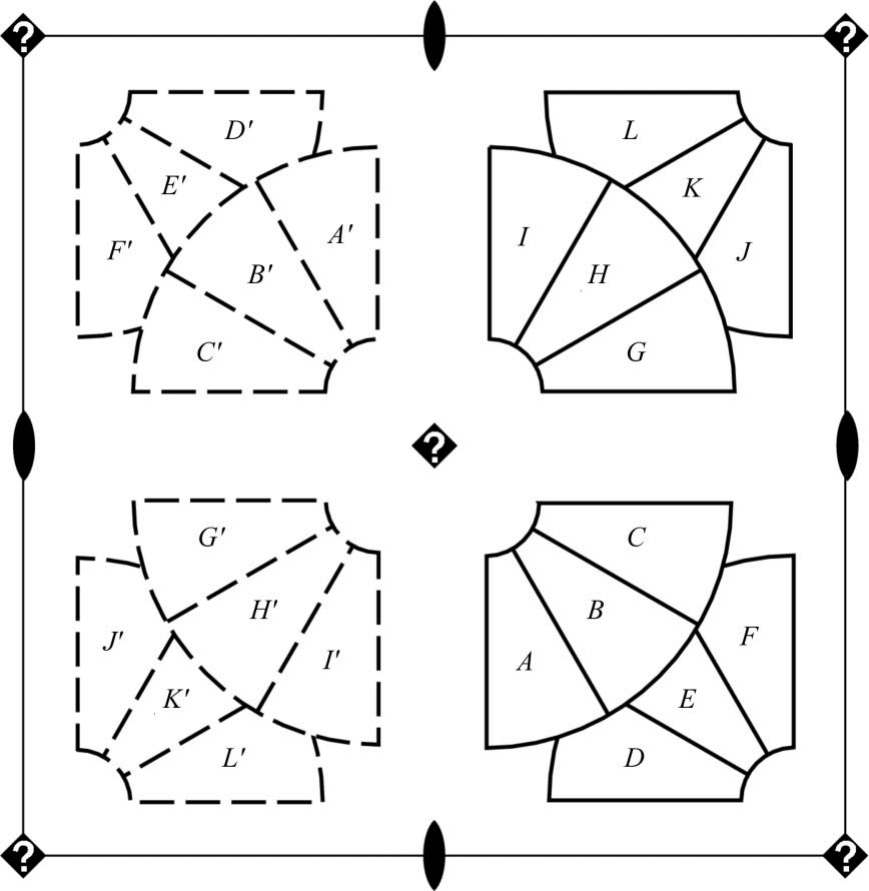
The process of constraining PDB entry 3b77 into a tetragonal space group, *P*4, starting with a model (structure *A*2) that is intentionally solved in space group *P*2. The monoclinic asymmetric unit contains 12 polypeptide chains (labeled *A*–*L*). The twofold operator **2** of space group *P*2 generates a second asymmetric unit populated by chains *A*′–*L*′. The trial fourfold (marked by ‘?’) maps chains *A*–*F* to chains *G*–*L* and maps chains *G*–*L* to chains *A*′–*F*′.

A simple example of chain matching is illustrated in Fig. 3 ▶. There are 12 identical polypeptides in the asymmetric unit of the monoclinic structure *A*2. Adapting the input space group (**H** = *P*2) into the target space group (**G** = *P*4) leads to the coset decomposition
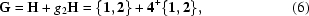
where the numerical symbols are intended to represent the identity operator **1**, the twofold rotation **2** of space group *P*2 and the fourfold *g*_2_ = **4** ^+^ chosen as the single left coset representative. Under the operation of *g*_2_, polypeptide chains *A*–*F* map to chains *G*–*L*, while chains *G*–*L* map to chains *A*′–­*F*′ in the second asymmetric unit of the monoclinic cell (corresponding to *h* = **2**).

#### 2.5.2. High-precision refinement of the origin shift

In the preceding section, the approximately known origin shift **x** is used to discover symmetry-matched peptide pairs. We now turn this process around, performing least-squares refinement on these known matches to produce the best possible chain alignment, while considering **x** to be a free variable. For these purposes we revert the atomic coordinates back to the candidate group setting (Fig. 1 ▶) prior to the application of the origin shift. The function to be minimized is the Cartesian square difference between chain-matched C^α^ positions,
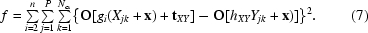
The outer summation here is over all *n* cosets except for the first one, which just produces the identity mapping. The middle sum is over all *P* polypeptide chains in the asymmetric unit and the inner sum is over the *N*_α_ C^α^ pairs in the *j*th matching pair of chains (*X*, *Y*). Operator *g*_*i*_ is the *i*th coset representative, while **t**_*XY*_ and *h*_*XY*_ are the translational and rotational symmetry operators in **H** required to produce a match between chains *X* and *Y* (5). Matrix **O** is the orthogonalization matrix required to convert fractional to Cartesian coordinates. After minimization of the function *f*, the refined origin shift is used to recalculate the optimized structure (Fig. 1 ▶).

Having determined the final origin shift, the input structure’s fit with target space group **G** can now be evaluated. If the structure is perfectly invariant when the coset representative operators are applied, the value of the function *f* will be identically zero. The deviation from perfect symmetry can be expressed as the root-mean-squared deviation of C^α^ atoms from their symmetry-predicted positions,
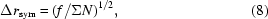
where Σ*N* symbolizes the total count of C^α^ matches over all matching polypeptide pairs and all cosets in the triple sum of (7).

#### 2.5.3. Generating coordinate sets corresponding to each asymmetric unit

Imposing additional symmetry on a structure implies that the number of unique polymer chains will be reduced; in fact, the resulting asymmetric unit will contain exactly *P*/*n* chains, the original number of chains divided by the number of cosets. The chain-matching results of §[Sec sec2.5.1]2.5.1 can be used to construct approximate models of the higher symmetry asymmetric unit. The key idea is to select one chain from each group of mutual chain matches; *e.g.* in Fig. 3 ▶ one chain is selected from each of the six groups {*A*, *G*}, {*B*, *H*}, {*C*, *I*}, {*D*, *J*}, {*E*, *K*}  and {*F*, *L*}. While there are many possible combinations [*n* ^(*P*/*n*)^], we take the simple expedient of selecting the polypeptide from each group that appears first in the original PDB input file, so in this case chains *A*–*F* are selected as the primary model of the asymmetric unit. To visualize the extent to which the input structure differs from the perfect symmetry of space group **G**, *n* − 1 additional models are then generated, one for each coset. These arise by looping over the polypeptides *X* of the primary model and transforming their matched polypeptides *Y* with
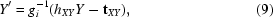
thus placing the matching chains and the primary model in approximate alignment. The end product of this exercise is a set of *n* different models of the higher symmetry asymmetric unit, nearly superimposed, with differences among models reflecting the NCS variability of the input structure. These models can be readily output as PDB-format files for visual inspection and further analysis. In the example of Fig. 3 ▶, the two models consist of chains *A*–*F* and *G*–*L*, respectively.

#### 2.5.4. Interpreting the deviation from perfect symmetry

Differences among these asymmetric unit (ASU) models combine two types of variation: a rigid-body component describing the motion of the asymmetric unit contents as a whole and a residual component reflecting the positions of individual atoms. The Δ*r*_sym_ measure of (8) contains both components, but it is also informative to separate the rigid-body and residual terms. To evaluate the residual component by itself, we perform a Kearsley (1989 ▶) alignment of the entire C^α^ contents of ASU models *i* and* j*, and evaluate the root mean-squared deviation of superimposed atoms, Δ*r*_*ij*_. Averaging this quantity over all 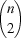 pairwise combinations of ASU models, the overall residual component can be expressed as
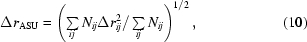
where *N*_*ij*_ is the total number of C^α^ matches between ASU models *i* and *j*. For cases where the ASU model contains more than one polypeptide chain, an additional measure of the residual term, Δ*r*_chain_, is defined to represent deviations of atoms within individual chains. This quantity is calculated in an identical manner to (10) except that the Kearsley alignment is performed on individual pairs of polypeptides and the resulting summation contains (*P*/*n*)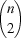 terms.

Values for Δ*r*_sym_, Δ*r*_ASU_ and Δ*r*_chain_ for structures *A*2 and *A*3 are reported in Table 4 ▶. The predominant contribution to the NCS differences in these structures is from random deviations of individual atoms of the order of 0.1 Å. There is only an insignificant contribution (0.002 Å in structure *A*2 and 0.03 Å in structure *A*3) from rigid-body rearrangements of poly­peptide chains.

### 2.6. Re-indexing the diffraction images in higher symmetry

We now suppose that a decision has been made to increase the symmetry of the atomic model. Clearly, the best outcome can be achieved by returning to the original diffraction images. Imposing the new space group **G** (*P*4 in the case of structures *A*2 and *A*3) on the original data will permit better unit-cell constraints for the prediction of spot positions during integration, afford more symmetry equivalents for outlier rejection during scaling and possibly remove model bias resulting from introducing too many free atoms during the model-building step.

Yet there are certain steps of the data-processing pipeline that would be wasteful to repeat. Since we already have an ensemble of models of the higher symmetry asymmetric unit, it is no longer necessary to repeat the decision during autoindexing in which the Bravais lattice and space group are chosen from a list of lattices compatible with the observed cell. Similarly, no phasing protocols should be required, as the structure of the atomic model and its position in the unit cell have adequately been addressed by the fast translation function (§[Sec sec2.4]2.4) and subsequent refinement (§[Sec sec2.5.2]2.5.2).

An express route to re-refinement is achieved by adapting the autoindexing program *labelit.index* (Sauter *et al.*, 2004 ▶) to accept the additional input of a PDB file containing one of the proposed ASU models from §[Sec sec2.5.3]2.5.3. Structure factors are calculated, taking into account a bulk-solvent correction (Afonine *et al.*, 2005 ▶) to more realistically model the observed intensities. Separately, data from one or two frames of the raw data are integrated and corrected for Lorentz and polarization factors (Leslie, 1999 ▶), using a preliminary reduced unit cell (Grosse-Kunstleve *et al.*, 2004 ▶) to model the lattice. We now wish to determine how the unit-cell basis vectors of the calculated and observed patterns need to be aligned in order to obtain the best fit between intensities. Two types of ambiguity need to be resolved. Firstly, in some cases the unit cell is close to fitting into a higher symmetry metric. A triclinic cell, *e.g.* with dimensions *a* ≃ *b* and α ≃ β, may require an axis swap (**a**′, **b**′, **c**′ = −**b**, −**a**, −**c**) to correctly model the observed pattern. Secondly, certain space groups permit multiple non-equivalent indexing schemes (Dauter, 1999 ▶), only one of which will allow the ASU model to align properly with the observations. For example, point groups 3, 4 and 6 can be indexed with the *c* axis up or down. All of these ambiguities can be resolved by exhaustively testing each possible re­indexing scheme that preserves the unit-cell dimensions, and assessing the mutual scaling *R* factor (Weiss, 2001 ▶) between calculated and observed intensities. The result is an indexing solution for the diffraction pattern that correctly accounts for the position and orientation of the ASU model in space group **G**. At this point the full data set is integrated, scaled and converted to structure factors. Structure refinement is initiated (*e.g.* with *phenix.refine*) starting with the aforementioned ASU model. As shown in Table 1 ▶, the re-refinement of triclinic structure *A*3 in space group *P*4, without any further manual intervention, leads to a new structure (*A*4) that is comparable to the original published PDB file.

## 3. Results and discussion

**Figure 4:**
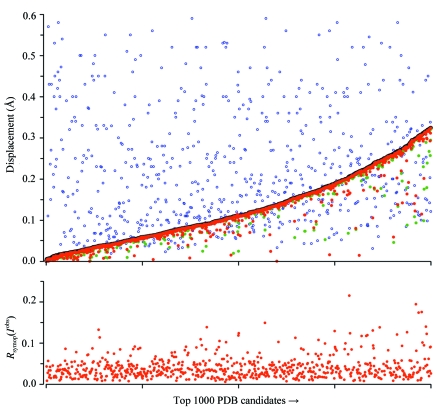
Top 1000 candidate structures for reassignment into a higher symmetry space group, ranked in increasing order of Δ*r*_sym_. (*a*) The average displacement needed to bring C^α^ atoms into a perfect symmetrical arrangement (Δ*r*_sym_, solid black line) is compared with the NCS-aligned displacements among alternate ASU models (Δ*r*_ASU_, red dots) and freestanding chains (Δ*r*_chain_, green dots), along with the estimated coordinate uncertainty for each structure (purple circles). (*b*) The maximal merging *R* factor for symmetry-equivalent reflections under the target space group for cases where observed intensities (*I* ^obs^) are available. PDB structures are only plotted here if Δ*r*_sym_ < 0.325 Å, if *R*_symop_ (*I* ^obs^) < 0.25 and if *R*_symop_ (*I* ^calc^) < 0.25 (*I* ^calc^ data are shown in Table S1).

A November 2009 snapshot of the PDB was analyzed to identify X-ray structures that are nearly invariant when additional rotational symmetry operators are imposed. Of almost 62 000 files in the database, about 53 000 are X-ray structures. Here, we focus on the approximately 52 000 that contain protein chains rather than exclusively nucleic acids or small peptides. About 1000 structures, or 2%, were conservatively found to produce a good fit with a higher symmetry space group. Fig. 4 ▶ ranks these candidates in order of increasing Δ*r*_sym_ (a measure of the average C^α^ displacement required to impose the additional symmetry; see equation 8) up to an arbitrary cutoff value (see below) of Δ*r*_sym_ = 0.325 Å. A full listing is given in Table S1^1^.

It is beyond the scope of this paper to deliver a definitive choice as to which space groups are best for individual structures. However, if the conservative group of 1000 shown in Fig. 4 ▶ is considered as a whole, there are strong arguments to favor the higher symmetry settings. Foremost is the small size of the displacements needed to bring equivalent atoms into a perfectly symmetrical arrangement. It is generally recognized that the coordinate accuracy of an X-ray structure is a fraction of the diffraction pattern’s limiting resolution (Luzzati, 1952 ▶). Various methods are presently used to estimate the coordinate uncertainty (Kleywegt, 2000 ▶) and where reported in the PDB these 1σ uncertainty values are plotted in Fig. 4 ▶(*a*). Most of the estimated values shown (75%) are at least as high as Δ*r*_sym_. Generally speaking then, for this group, there is a good chance that displacements seeming to be a product of noncrystallographic symmetry differences are really a result of experimental coordinate uncertainty.

This argument is made stronger by a considering whether the imposition of added symmetry requires random displacements of individual atoms or rigid-body motions of entire polypeptide chains. The quantity Δ*r*_ASU_ (10) gives an indication of the random variations of equivalent atoms once the polypeptide chains are superimposed by a rigid-body motion (Δ*r*_chain_ serves the same function for cases where there are multiple chains in the asymmetric unit). The plotted values in Fig. 4 ▶(*a*) demonstrate that for most cases Δ*r*_ASU_ (or Δ*r*_chain_ where appropriate) is nearly identical to Δ*r*_sym_; on average, all but 0.01 Å of the displacement required comes from individual atomic motions. The fact that there is virtually no rigid-body component is consistent with the idea that subunit differences are a consequence of experimental uncertainties rather than true observations of NCS variation.

A final and compelling factor supporting the higher sym­metries is the distribution of observed structure factors, which are published in the PDB for 694 of the cases. The agreement of symmetry-equivalent observed intensities is quite good under many of the higher symmetry operators, with values of *R*_symop_ (*I* ^obs^) clustering about an average of 4% (Fig. 4 ▶ *b*). The fact that the reported merging *R* values from these same 694 structures have an average of 8% suggests that any observed differences in symmetry-equivalent intensities is not experimentally significant.

Taken together, the data in Fig. 4 ▶ are evidence that a con­siderable number of PDB structures could be reassigned to higher symmetry space groups. Reassigning the space group would reduce the number of polypeptide chains in the model by a factor of *n*, where *n* = 2 for most cases but in some cases is found to be 3, 4, 6 or even 12 (Table 5 ▶). It is not apparent whether the reassignment candidates have any particular properties in common, *e.g.* they seem to be distributed over the entire range of limiting resolutions represented in the PDB. Furthermore, all point groups for which supergroups are available are present in the list (Tables 6 ▶ and S1).

Care should be taken to distinguish between the present results and a previous study by Wang & Janin (1993 ▶) showing that NCS symmetry axes tend to lie nearly parallel to unit-cell edges or face or body diagonals. The vast majority of structures listed by Wang and Janin are likely to have correctly classified space groups, with verifiable differences between NCS-related subunits. None of the cases listed in that paper appear in our list of candidates for reclassification (Table S1).

The choice of Δ*r*_sym_ = 0.325 Å as a cutoff for producing Fig. 4 ▶ and Table S1, while arbitrary, reflects the notion that larger values of Δ*r*_sym_ and Δ*r*_chain_ are more likely to exceed the expected coordinate uncertainty, implying confident pseudosymmetry rather than underassigned symmetry. It is instructional to consider how these latter categorizations relate to the mathematical treatment of §[Sec sec2.5.1]2.5.1: with underassigned sym­metry the coset representatives *g*_2_…*g*_*n*_ are exact symmetry operators leaving the structure invariant, while with pseudosymmetry these operators match atoms in the asymmetric unit in an approximate rather than an exact fashion. Furthermore, with pseudosymmetry there is the attendant possibility of merohedral twinning (Padilla & Yeates, 2003 ▶), in which the coset representatives act as twinning operators that describe the mutual relationship of different unit cells in the crystal. The *R*_symop_(*g*_*i*_) values obtained from (1) correspond to the *R*_twin_ formula defined by Lebedev *et al.* (2006 ▶), suggesting a role for the *R*_symop_ (*I* ^calc^) and *R*_symop_ (*I* ^obs^) statistics in quantifying twinning, as discussed in that reference.

Ideally, any validation process to prepare structures for final publication and deposition should scrutinize the choice of space group. Normally the compatible Bravais lattices are evident at the stage of autoindexing, when the observed unit-cell dimensions are checked for higher symmetry metrics. Subsequently, at the step of data-set merging, it is usually possible to unambiguously identify the point group of the diffraction pattern. Yet the data shown here indicate that a fraction of cases are misassigned, suggesting that a third check should be added at a later step, after the atomic model is built.

It is fair to ask how beneficial such a procedure would be. In the unusual but ideal situation in which the data are very accurately measured and there is an ample data-to-parameter ratio, it should be possible to obtain an accurate structure even if the symmetry is underassigned. However, in more typical cases in which the desired atomic details may be only marginally observable in the electron-density map, the con­straints offered by perfect symmetry may be crucial to map interpretation. Much of crystallography today is centered on elucidating the relationship between proteins and small-molecular ligands, including ions, saccharides, lipids, nucleotides, drugs and small peptides, and the models for these interactions may not be as well restrained by stereochemistry as those of proteins. Assignment into a higher symmetry may prove helpful in borderline cases where it is barely possible to discern the ligand. The ability to align symmetry-equivalent models arising from space-group reassignment (explained in §[Sec sec2.5.3]2.5.3) is intended to assist the crystallographer in determining whether there are regions of the model that may exhibit especially large changes under the proposed symmetry target and which therefore warrant extra attention.

The spectre of re-evaluating the space groups assigned to hundreds of crystal structures calls to mind recent discussions regarding the worth of archiving original crystallographic diffraction images (see, for example,  Baker *et al.*, 2008 ▶). If the objective is to justify a certain choice of symmetry to future investigators, then data archival assumes a new importance.

The procedures described here are included in the software package *LABELIT*, available for download by noncommercial users at http://cci.lbl.gov/labelit and for licensing by commercial users. Command-line parameters for the program *labelit.check_pdb_symmetry*, explained in the online manual, permit the input of both coordinates and structure factors. *LABELIT* is also included with the *PHENIX* package (Adams *et al.*, 2002 ▶), available for download at http://www.phenix-online.org.

## Supplementary Material

Supplementary material file. DOI: 10.1107/S0907444910001502/dz5193sup1.pdf

## Figures and Tables

**Table 1:** Refinement statistics for the alternate 3b77 models The data collected by the JCSG consisted of 90 1° rotation photographs acquired from a single crystal on ALS beamline 8.2.2 (X-ray wavelength 0.9795 Å).

Solution	3b77(published)	*A*1 (resolved)	*A*2 (resolved)	*A*3 (resolved)	*A*4 (re-indexed)
Space group	*P*4	*P*4	*P*121	*P*1	*P*4
No. of chains	6	6	12	24	6
Unit-cell parameters					
*a* (Å)	151.0	151.1	151.0	76.3	151.0
*b* (Å)	151.0	151.1	76.3	151.0	151.0
*c* (Å)	76.2	76.3	151.1	151.1	76.2
α, β, γ (°)	90	90	∼90	∼90	90
Resolution (Å)	47.7–2.42	67.6–3.5	67.6–3.5	62.1–3.5	67.5–2.42
No. of unique reflections	65460	21837	40536	48677	57641
Completeness (%)	99.7	99.3	92.2	57.3	87.6
Free-*R* test-set size (%)	5.1	3.9	3.8	3.9	3.1
Refinement statistics					
*R*/*R*_free_† (%)	21.4/25.4	18.7/21.9	18.2/21.3	17.3/21.3	22.6/27.1
R.m.s.d. bond lengths (Å)	0.015	0.010	0.010	0.009	0.009
R.m.s.d. bond angles (°)	1.50	1.18	1.21	1.12	1.15
Estimated coordinate error (Å)	0.23	0.30	0.30	0.34	0.42

† *R* and *R*_free_ = 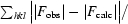 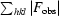 for either the working set (*R*) or the test set (*R*_free_).

**Table 2:** Symmetry-operator reliabilities (*R*_symop_) for alternate models (%) Statistical values are computed to a limiting resolution of 3.5 Å.

Operator short notation†	Model *A*2 (*I*^calc^)	Model *A*2 (*I*^obs^)	Model *A*3 (*I*^calc^)	Model *A*3 (*I*^obs^)
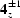	1.6	3.8	2.5	4.3
**2**_*z*_	0.0	0.0	2.8	3.6
**2**_*y*_	27.4	42.2	27.3	42.8
**2**_*x*_	27.4	42.2	27.3	42.9
**2**_*xy*_	27.4	42.2	27.3	42.4
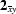	27.4	42.2	27.3	42.1

† Rotation-axis directions are expressed in the reference setting of the tetragonal structure, *A*1, thus the fourfold along *z*.

**Table 3:** Potential Patterson settings that fit the unit cell based on structure *A*3 Statistical values are computed to a limiting resolution of 3.5 Å.

Patterson setting {rotational operators}	No. of polypeptide chains	Le Page δ (°)	Maximum *R*_symop_ (*I*^calc^) (%)	Maximum *R*_symop_ (*I*^obs^) (%)	Plausible
*P*4/*mmm* { 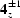 , **2**_*z*_, **2**_*y*_, **2**_*x*_, **2**_*xy*_, 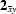 , **1**}	3	0.046	27.3	42.9	No
*P*4/*m* { 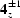 , **2**_*z*_, **1**}	6	0.046	2.8	4.3	Yes
*Cmmm* {**2**_*z*_, **2**_*xy*_, 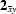 , **1**}	6	0.046	27.3	42.4	No
*Pmmm* {**2**_*z*_, **2**_*y*_, **2**_*x*_, **1**}	6	0.032	27.3	42.9	No
*C*12/*m*1 {**2**_*xy*_, **1**}	12	0.046	27.3	42.4	No
*C*12/*m*1 { 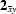 , **1**}	12	0.046	27.3	42.1	No
*P*12/*m*1 {**2**_*z*_, **1**}	12	0.030	2.8	3.6	Yes
*P*12/*m*1 {**2**_*x*_, **1**}	12	0.032	27.3	42.9	No
*P*12/*m*1 {**2**_*y*_, **1**}	12	0.010	27.3	42.8	No
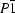 {**1**}	24	0.000	0.0	0.0	Yes

**Table 4:** Higher symmetry scoring parameters

	Structure *A*3	Structure *A*2
Input symmetry	*P*1	*P*121
Target symmetry	*P*4	*P*4
No. of cosets	4	2
Maximum Φ(*G*_*i*_)	0.0020	0.0015
Δ*r*_sym_ (Å)	0.107	0.110
Δ*r*_ASU_ (Å)	0.102	0.110
Δ*r*_chain_ (Å)	0.075	0.108

**Table 5:** Decrease in subunit redundancy upon imposing higher symmetry for the group of structures in Fig. 4 ▶

Coset count (redundancy factor)	No. of cases (total 1000)
2	893
3	44
4	50
6	12
12	1

**Table 6:** Cases of near-crystallographic symmetry in Fig. 4 ▶, sorted by published point group

Point group of the published structure	No. of cases (total 1000)	Total in PDB (total 51924†)	Fraction reassigned (%)
1	134	1759	7.6
2	299	12928	2.3
222	76	19072	0.4
4	132	1278	3.0
422	2	5353	0.0
3	186	1332	14.0
321	17	4789	0.4
312	6	114	5.3
23	16	740	2.2
6	132	1884	7.0
622	—	2236	—
432	—	439	—

† Number of polypeptide structures in the database.

## Footnotes

1 Supplementary material has been deposited in the IUCr electronic archive (Reference: DZ5193). Services for accessing this material are described at the back of the journal.

## References

[bb1] Afonine, P. V., Grosse-Kunstleve, R. W. & Adams, P. D. (2005). *Acta Cryst.* D**61**, 850–855. 10.1107/S0907444905007894 PMC2808320 15983406

[bb2] Afonine, P. V., Grosse-Kunstleve, R. W., Adams, P. D., Lunin, V. Y. & Urzhumtsev, A. (2007). *Acta Cryst.* D**63**, 1194–1197. 10.1107/S0907444907046148 PMC2808317 18007035

[bb3] Adams, P. D., Grosse-Kunstleve, R. W., Hung, L.-W., Ioerger, T. R., McCoy, A. J., Moriarty, N. W., Read, R. J., Sacchettini, J. C., Sauter, N. K. & Terwilliger, T. C. (2002). *Acta Cryst.* D**58**, 1948–1954. 10.1107/s0907444902016657 12393927

[bb4] Baker, E. N., Dauter, Z., Guss, M. & Einspahr, H. (2008). *Acta Cryst.* D**64**, 337–338. 10.1107/S0907444908004915 18391400

[bb5] Berman, H., Henrick, K. & Nakamura, H. (2003). *Nature Struct. Biol.* **10**, 980. 10.1038/nsb1203-980 14634627

[bb6] Boisen, M. B. Jr & Gibbs, G. V. (1990). *Mathematical Crystallography (Reviews in Minerology, Vol. 15)*, revised ed. Washington DC: Mineralogical Society of America.

[bb7] Dauter, Z. (1999). *Acta Cryst.* D**55**, 1703–1717. 10.1107/s0907444999008367 10531520

[bb8] Evans, P. (2006). *Acta Cryst.* D**62**, 72–82. 10.1107/S0907444905036693 16369096

[bb9] Giacovazzo, G., Monaco, H. L., Vitergo, D., Scordari, F., Gilli, G., Zonotti, G. & Catti, M. (1992). *Fundamentals of Crystallography.* Chester, Oxford: IUCr/Oxford University Press.

[bb10] Grosse-Kunstleve, R. W. (1999). *Acta Cryst.* A**55**, 383–395. 10.1107/s0108767398010186 10927267

[bb11] Grosse-Kunstleve, R. W., Sauter, N. K. & Adams, P. D. (2004). *Acta Cryst.* A**60**, 1–6. 10.1107/s010876730302186x 14691322

[bb12] Grosse-Kunstleve, R. W., Sauter, N. K., Moriarty, N. W. & Adams, P. D. (2002). *J. Appl. Cryst.* **35**, 126–136.

[bb13] Grosse-Kunstleve, R. W., Zwart, P. H., Afonine, P. V., Ioerger, T. R. & Adams, P. D. (2006). *Newsl. IUCr Comm. Crystallogr. Comput.* **7**, 92–105.

[bb14] Hahn, T. (1996). Editor. *International Tables for Crystallography*, Vol. *A*, 4th ed. Dordrecht: Kluwer Academic Publishers.

[bb15] Hendrickson, W. A. (1985). *Methods Enzymol.* **115**, 252–270. 10.1016/0076-6879(85)15021-4 3841182

[bb16] Hirshfeld, F. L. (1968). *Acta Cryst.* A**24**, 301–311.

[bb17] Hooft, R. W. W., Sander, C. & Vriend, G. (1994). *J. Appl. Cryst.* **27**, 1006–1009.

[bb18] Hooft, R. W. W., Vriend, G., Sander, C. & Abola, E. E. (1996). *Nature (London)*, **381**, 272. 10.1038/381272a0 8692262

[bb19] Jones, T. A. & Liljas, L. (1984). *Acta Cryst.* A**40**, 50–57.

[bb20] Kearsley, S. K. (1989). *Acta Cryst.* A**45**, 208–210.

[bb21] Kleywegt, G. J. (2000). *Acta Cryst.* D**56**, 249–265. 10.1107/s0907444999016364 10713511

[bb22] Kleywegt, G. J., Hoier, H. & Jones, T. A. (1996). *Acta Cryst.* D**52**, 858–863. 10.1107/S0907444995008936 15299651

[bb23] Koch, E. & Fischer, W. (1996). *International Tables for Crystallo­graphy*, Vol. *A*, 4th ed., edited by T. Hahn, pp. 855–869. Dordrecht: Kluwer Academic Publishers.

[bb24] Lebedev, A. A., Vagin, A. A. & Murshudov, G. N. (2006). *Acta Cryst.* D**62**, 83–95. 10.1107/S0907444905036759 16369097

[bb25] Le Page, Y. (1982). *J. Appl. Cryst.* **15**, 255–259.

[bb26] Le Page, Y. (1988). *J. Appl. Cryst.* **21**, 983–984.

[bb27] Leslie, A. G. W. (1999). *Acta Cryst.* D**55**, 1696–1702. 10.1107/s090744499900846x 10531519

[bb28] Luzzati, V. (1952). *Acta Cryst.* **5**, 802–810.

[bb30] Marsh, R. E. (1995). *Acta Cryst.* B**51**, 897–907.

[bb29] Marsh, R. E. (1997). *Acta Cryst.* B**53**, 317–322.

[bb31] Marsh, R. E. (2009). *Acta Cryst.* B**65**, 782–783. 10.1107/S0108768109046448 19923706

[bb32] Marsh, R. E. & Herbstein, F. H. (1988). *Acta Cryst.* B**44**, 77–88. 10.1107/s0108768187009492 3271104

[bb33] Marsh, R. E. & Spek, A. L. (2001). *Acta Cryst.* B**57**, 800–805. 10.1107/S0108768101014331 11717479

[bb34] McCoy, A. J., Grosse-Kunstleve, R. W., Adams, P. D., Winn, M. D., Storoni, L. C. & Read, R. J. (2007). *J. Appl. Cryst.* **40**, 658–674. 10.1107/S0021889807021206 PMC2483472 19461840

[bb35] Navaza, J. & Vernoslova, E. (1995). *Acta Cryst.* A**51**, 445–449.

[bb36] Padilla, J. E. & Yeates, T. O. (2003). *Acta Cryst.* D**59**, 1124–1130. 10.1107/s0907444903007947 12832754

[bb37] Palatinus, L. & van der Lee, A. (2008). *J. Appl. Cryst.* **41**, 975–984.

[bb38] Sauter, N. K., Grosse-Kunstleve, R. W. & Adams, P. D. (2004). *J. Appl. Cryst.* **37**, 399–409. 10.1107/S0021889804005874 PMC2808709 20090869

[bb39] Sauter, N. K., Grosse-Kunstleve, R. W. & Adams, P. D. (2006). *J. Appl. Cryst.* **39**, 158–168.

[bb41] Spek, A. L. (2009). *Acta Cryst.* D**65**, 148–155. 10.1107/S090744490804362X PMC2631630 19171970

[bb42] Terwilliger, T. C., Grosse-Kunstleve, R. W., Afonine, P. V., Moriarty, N. W., Zwart, P. H., Hung, L.-W., Read, R. J. & Adams, P. D. (2008). *Acta Cryst.* D**64**, 61–69. 10.1107/S090744490705024X PMC2394820 18094468

[bb43] Urzhumtseva, L., Afonine, P. V., Adams, P. D. & Urzhumtsev, A. (2009). *Acta Cryst.* D**65**, 297–300. 10.1107/S0907444908044296 PMC2651759 19237753

[bb44] Wang, X. & Janin, J. (1993). *Acta Cryst.* D**49**, 505–512. 10.1107/S0907444993003737 15299486

[bb45] Weiss, M. S. (2001). *J. Appl. Cryst.* **34**, 130–135.

[bb46] Zwart, P. H., Grosse-Kunstleve, R. W. & Adams, P. D. (2005). *CCP4 Newsl.* **43**, contribution 7.

[bb47] Zwart, P. H., Grosse-Kunstleve, R. W., Lebedev, A. A., Murshudov, G. N. & Adams, P. D. (2008). *Acta Cryst.* D**64**, 99–107. 10.1107/S090744490705531X PMC2394827 18094473

