# Supplementary material for: Detection and correction of underassigned rotational symmetry prior to structure deposition
Source: Acta Crystallogr D Biol Crystallogr. 2010 Apr 21;66(Pt 5):503–13. doi: 10.1107/S0907444910001502 (PMC2865365; doi:10.1107/S0907444910001502)
Supplement: Supplementary file 1 [file d-66-00503-sup1.pdf]

Table S1. PDB entries with near-crystallographic symmetry

PDB Code	Resolution (Å)	Published Symmetry	Candidate Symmetry	No. of Cosets	Chains / asu	δ (°)	R_{symop} (I^{calc}) (%)	$\varphi(g_i)$	Δr_{sym} (Å)	Δr_{asu} (Å)	Δr_{chain} (Å)	Coord. Error (Å)	R_{symop} (I^{obs}) (%)
**1qu9**	1.2	P 2 2 2	P 2 3	3	1	0.00	0.0	0.000	0.00	0.00	0.00	---	---
**2fiv**	2.0	P 31	P 31 2 1	2	1	0.00	0.6	0.000	0.00	0.00	0.00	---	4.3
**2a8k**	1.5	P 31	P 31 2 1	2	2	0.00	0.6	0.002	0.01	0.00	0.00	---	0.9
**1jvl**	2.0	P 61	P 61 2 2	2	1	0.00	0.3	0.000	0.01	0.00	0.00	---	---
**2b7k**	1.8	P 1 21 1	P 21 21 2	2	2	0.00	0.9	0.001	0.01	0.01	0.00	---	2.8
**3dg7**	2.0	C 1 2 1	I 4 2 2	4	1	0.02	1.0	0.001	0.01	0.01	0.01	0.11	5.7
**1iwo**	3.1	P 41	P 41 21 2	2	1	0.00	0.2	0.000	0.01	0.00	0.00	0.57	---
**1bwv**	2.4	P 64	P 64 2 2	2	4	0.00	1.1	0.001	0.01	0.01	0.00	---	3.4
**3g3q**	2.6	P 32	P 32 2 1	2	1	0.00	0.2	0.000	0.01	0.00	0.00	0.43	1.7
**1sma**	2.8	P 61	P 61 2 2	2	1	0.00	6.4	0.007	0.01	0.00	0.00	---	2.3
**3fiv**	1.9	P 31	P 31 2 1	2	1	0.00	0.9	0.000	0.01	0.01	0.01	---	4.2
**2gpz**	2.5	P 6	P 6 2 2	2	1	0.00	0.3	0.000	0.01	0.00	0.00	0.24	3.4
**1jig**	1.5	R 3 :H	F 2 3	4	1	0.00	1.5	0.007	0.01	0.01	0.01	0.15	1.5
**1xuv**	2.1	C 1 2 1	P 32 2 1	3	1	0.00	0.7	0.001	0.01	0.01	0.01	0.27	---
**1ru8**	2.7	P 21 21 21	P 41 21 2	2	1	0.01	0.7	0.000	0.01	0.01	0.01	0.43	---
**3ebw**	2.8	P 41	P 41 21 2	2	1	0.00	0.3	0.000	0.01	0.01	0.01	---	---
**3k9f**	2.9	P 32	P 62	2	2	0.00	1.4	0.000	0.01	0.01	0.00	0.61	---
**1qy7**	2.0	I 41 2 2	I 41 3 2	3	1	0.01	4.1	0.001	0.01	0.01	0.01	---	4.6
**1ki1**	2.3	P 1 21 1	C 2 2 21	2	2	0.00	0.5	0.000	0.01	0.00	0.00	---	---
**2gsh**	2.4	P 2 3	P 4 3 2	2	1	0.00	0.5	0.000	0.01	0.01	0.01	0.21	---
**2z0g**	2.1	R 3 :H	I 2 3	4	1	0.01	0.9	0.006	0.01	0.01	0.01	0.18	1.7
**1izy**	2.8	P 65	P 65 2 2	2	1	0.00	0.7	0.000	0.01	0.01	0.01	0.40	2.0
**2r8a**	3.0	P 41	P 41 21 2	2	1	0.00	0.9	0.000	0.01	0.00	0.00	0.53	2.0
**2agv**	2.4	P 41	P 41 21 2	2	1	0.00	0.7	0.000	0.01	0.01	0.01	0.45	---
**1xdk**	2.9	P 31 2 1	P 61 2 2	2	4	0.00	0.4	0.000	0.01	0.01	0.01	0.80	2.0
**1okb**	1.9	P 1 21 1	C 2 2 21	2	1	0.06	0.8	0.001	0.01	0.01	0.01	0.23	---
**3eyy**	2.4	P 31	P 31 2 1	2	1	0.00	2.0	0.002	0.01	0.01	0.01	0.37	1.7
**3eg5**	2.7	P 32	P 32 2 1	2	2	0.00	0.5	0.000	0.01	0.01	0.01	0.25	3.2
**2b7j**	2.3	P 1 21 1	P 21 21 2	2	2	0.00	1.9	0.002	0.01	0.01	0.01	---	---
**1pq3**	2.7	P 32	P 32 2 1	2	3	0.00	0.7	0.000	0.01	0.01	0.01	---	---
**1x90**	2.7	R 3 :H	R 3 2 :H	2	1	0.00	1.5	0.002	0.02	0.01	0.01	0.42	3.6
**1z2i**	2.2	P 31	P 31 2 1	2	2	0.00	4.5	0.003	0.02	0.01	0.01	0.46	4.4
**1g5u**	3.1	P 32	P 32 2 1	2	1	0.00	0.6	0.000	0.02	0.01	0.01	0.58	---
**2z4r**	3.0	C 1 2 1	R 3 2 :H	3	1	0.02	2.3	0.002	0.02	0.01	0.01	0.64	3.3
**1sms**	3.1	P 61	P 61 2 2	2	1	0.00	1.5	0.003	0.02	0.01	0.01	---	---
**1s3n**	2.5	I 41	I 41 2 2	2	1	0.00	0.9	0.001	0.02	0.01	0.01	0.44	1.0
**3bp3**	1.6	C 2 2 21	P 43 21 2	2	1	0.02	1.0	0.000	0.02	0.01	0.01	0.40	3.6
**2g4c**	3.2	P 32	P 65	2	2	0.00	2.3	0.003	0.02	0.02	0.01	---	4.7
**3eqy**	1.6	P 31	P 61	2	2	0.00	1.8	0.000	0.02	0.02	0.02	0.04	1.3
**2pjx**	3.1	P 21 3	P 43 3 2	2	2	0.00	0.4	0.000	0.02	0.02	0.02	0.47	1.8
**3e8d**	2.7	P 32	P 65	2	2	0.00	6.0	0.003	0.02	0.02	0.01	0.29	---
**1bvs**	3.0	P 31	P 64 2 2	4	2	0.00	1.6	0.001	0.02	0.01	0.01	0.77	2.8
**1p7h**	2.6	P 1	C 1 2 1	2	2	0.03	1.4	0.002	0.02	0.01	0.01	0.54	2.5

PDB Code	Resolution (Å)	Published Symmetry	Candidate Symmetry	No. of Cosets	Chains / asu	δ (°)	R_{symop} (I^{calc}) (%)	φ(g_i)	Δr_{sym} (Å)	Δr_{asu} (Å)	Δr_{chain} (Å)	Coord. Error (Å)	R_{symop} (I^{obs}) (%)
**2al5**	1.6	P 1 21 1	P 21 21 2	2	1	0.07	1.8	0.002	0.02	0.02	0.02	0.17	3.6
**3cqf**	3.1	C 2 2 21	P 43 21 2	2	1	0.00	0.4	0.000	0.02	0.02	0.02	0.27	2.5
**1r95**	2.6	P 62	P 62 2 2	2	1	0.00	0.7	0.000	0.02	0.02	0.02	0.17	---
**3djb**	2.9	P 43	P 43 21 2	2	1	0.00	0.5	0.002	0.02	0.01	0.01	0.63	---
**1m22**	1.4	P 1 21 1	P 21 21 21	2	1	0.00	1.9	0.001	0.02	0.02	0.02	0.07	3.5
**3g5c**	2.4	R 3 :H	R 3 2 :H	2	1	0.00	3.0	0.002	0.02	0.02	0.02	---	3.7
**2g4d**	2.8	P 43	P 43 2 2	2	2	0.00	1.4	0.001	0.02	0.02	0.01	---	5.7
**1m21**	1.8	P 1 21 1	P 21 21 21	2	1	0.00	1.2	0.002	0.02	0.02	0.02	0.06	8.6
**2a0s**	2.2	R 3 :H	R 3 2 :H	2	1	0.00	3.1	0.001	0.02	0.02	0.02	0.14	3.4
**2cs7**	1.2	C 1 2 1	P 31 2 1	3	1	0.01	4.5	0.002	0.02	0.02	0.02	0.01	---
**2r88**	2.6	C 2 2 21	P 41 21 2	2	1	0.03	0.8	0.000	0.02	0.02	0.02	0.39	1.8
**1hci**	2.8	P 31	P 31 2 1	2	1	0.00	1.6	0.001	0.02	0.01	0.01	0.78	4.9
**1xal**	2.8	P 43	P 43 21 2	2	1	0.00	1.4	0.001	0.02	0.01	0.01	0.66	3.2
**1ow0**	3.1	P 3	P 3 2 1	2	2	0.00	0.5	0.000	0.02	0.01	0.01	0.77	3.2
**2wg6**	2.5	P 1 21 1	P 63	3	4	0.03	1.3	0.005	0.02	0.02	0.02	0.16	2.8
**3e87**	2.3	P 32	P 65	2	2	0.00	4.1	0.002	0.02	0.02	0.01	0.20	3.9
**2i14**	2.9	P 31	P 31 2 1	2	3	0.00	0.8	0.002	0.02	0.02	0.01	0.66	2.8
**3dev**	3.1	P 3	P 3 2 1	2	1	0.00	1.0	0.001	0.02	0.02	0.02	0.67	---
**3bry**	3.2	F 2 2 2	I 41 2 2	2	1	0.02	1.0	0.000	0.02	0.02	0.02	0.32	2.4
**2zjt**	2.8	P 21 21 21	P 43 21 2	2	1	0.07	0.7	0.000	0.02	0.02	0.02	0.64	2.5
**2hb6**	2.0	P 63	P 63 2 2	2	1	0.00	1.8	0.002	0.02	0.02	0.02	0.07	1.6
**1uiz**	2.5	R 3 :H	P 21 3	4	1	0.02	0.9	0.002	0.02	0.01	0.01	---	---
**3co7**	2.9	P 32	P 32 2 1	2	1	0.00	0.4	0.000	0.02	0.02	0.02	---	---
**2a0k**	1.8	C 1 2 1	C 2 2 21	2	1	0.03	4.6	0.003	0.02	0.02	0.02	0.08	5.4
**2gh1**	2.5	P 32	P 32 2 1	2	1	0.00	0.9	0.001	0.02	0.02	0.02	0.34	4.3
**1t6s**	1.9	P 62	P 62 2 2	2	1	0.00	1.2	0.000	0.02	0.02	0.02	---	1.2
**1wpg**	2.3	P 1 21 1	P 21 21 21	2	2	0.01	0.8	0.000	0.02	0.02	0.01	0.40	---
**2e2e**	2.0	P 1 21 1	P 21 21 21	2	1	0.01	0.9	0.000	0.02	0.02	0.02	0.16	---
**2fbk**	2.3	P 61	P 61 2 2	2	1	0.00	0.9	0.002	0.02	0.00	0.00	---	1.3
**2dfy**	1.6	P 3	P 3 1 2	2	1	0.00	1.7	0.001	0.02	0.02	0.02	0.05	---
**3gvz**	2.8	P 1 21 1	C 2 2 21	2	1	0.03	1.2	0.000	0.02	0.01	0.01	0.06	---
**2p88**	2.4	P 1 21 1	C 2 2 21	2	4	0.00	1.1	0.001	0.02	0.01	0.01	0.35	5.5
**1mo1**	1.8	P 43	P 43 2 2	2	2	0.00	4.6	0.004	0.02	0.02	0.02	0.12	1.4
**1udd**	2.2	P 32	P 32 2 1	2	2	0.00	6.2	0.006	0.02	0.02	0.02	0.25	2.6
**1s3l**	2.4	I 41	I 41 2 2	2	1	0.00	1.6	0.002	0.02	0.02	0.02	0.34	---
**3evx**	2.5	P 1	P 1 21 1	2	2	0.07	2.7	0.001	0.02	0.02	0.02	0.40	7.8
**2j50**	3.0	P 21 21 21	P 41 21 2	2	1	0.03	0.7	0.000	0.02	0.01	0.01	0.67	3.6
**2okf**	1.6	P 31	P 31 2 1	2	1	0.00	2.8	0.001	0.02	0.02	0.02	0.04	---
**1r94**	2.3	P 62	P 62 2 2	2	1	0.00	0.8	0.001	0.02	0.02	0.02	0.17	---
**2jaq**	2.3	P 31	P 31 2 1	2	1	0.00	3.6	0.002	0.02	0.00	0.00	0.19	3.0
**1f9w**	2.5	P 43	P 43 21 2	2	1	0.00	2.1	0.001	0.02	0.01	0.01	---	4.6
**2pyn**	1.9	P 61	P 61 2 2	2	1	0.00	3.8	0.004	0.03	0.02	0.02	0.10	1.3
**1r5w**	2.9	P 41	P 41 21 2	2	3	0.00	0.8	0.001	0.03	0.02	0.02	---	---
**1ooh**	1.2	P 43	P 43 21 2	2	1	0.00	6.1	0.005	0.03	0.03	0.03	0.04	---
**1rpm**	2.3	P 1 21 1	C 2 2 21	2	1	0.09	1.9	0.001	0.03	0.02	0.02	---	2.4

PDB Code	Resolution (Å)	Published Symmetry	Candidate Symmetry	No. of Cosets	Chains / asu	δ (°)	R_{symop} (I^{calc}) (%)	φ(g_i)	Δr_{sym} (Å)	Δr_{asu} (Å)	Δr_{chain} (Å)	Coord. Error (Å)	R_{symop} (I^{obs}) (%)
**2pih**	2.1	P 1	C 1 2 1	2	1	0.07	1.4	0.002	0.03	0.02	0.02	0.50	---
**3b54**	3.1	F 2 3	F 41 3 2	2	1	0.00	0.8	0.000	0.03	0.03	0.03	---	---
**1xfx**	3.2	C 1 2 1	P 65 2 2	6	2	0.02	0.7	0.000	0.03	0.03	0.03	0.79	1.8
**3bs0**	2.6	P 1	P 1 21 1	2	1	0.06	1.3	0.007	0.03	0.01	0.01	0.50	4.6
**1am4**	2.7	P 1	R 3 :H	3	2	0.04	1.2	0.002	0.03	0.02	0.02	---	---
**3glm**	2.5	C 1 2 1	C 2 2 21	2	2	0.02	2.6	0.001	0.03	0.03	0.03	0.22	5.5
**1hm8**	2.5	R 3 :H	R 3 2 :H	2	1	0.00	1.7	0.001	0.03	0.03	0.03	0.33	1.8
**1uum**	2.3	P 3	P 63	2	1	0.00	1.5	0.000	0.03	0.03	0.03	0.24	2.1
**1ii4**	2.7	P 1	P 1 21 1	2	4	0.02	3.9	0.004	0.03	0.03	0.02	---	---
**2deu**	3.4	C 1 2 1	I 21 21 21	2	1	0.01	4.0	0.002	0.03	0.02	0.02	1.05	3.6
**3e88**	2.5	P 32	P 65	2	2	0.00	3.8	0.002	0.03	0.03	0.02	0.26	5.0
**2f62**	1.5	C 1 2 1	C 2 2 21	2	1	0.03	4.5	0.003	0.03	0.03	0.03	0.04	2.2
**2z66**	1.9	P 1	P 21 21 2	4	1	0.15	2.1	0.002	0.03	0.03	0.03	0.09	5.0
**3eaa**	2.8	P 6	P 6 2 2	2	1	0.00	1.6	0.001	0.03	0.02	0.02	0.43	---
**3elp**	2.4	P 42	P 42 21 2	2	2	0.00	1.1	0.002	0.03	0.03	0.03	---	3.3
**2gw3**	1.4	C 1 2 1	I 2 2 2	2	1	0.01	8.5	0.009	0.03	0.03	0.03	---	1.7
**2wg5**	2.1	P 1 21 1	P 63	3	4	0.12	1.5	0.000	0.03	0.03	0.03	---	2.3
**3e9h**	2.1	P 1 21 1	P 21 21 21	2	2	0.05	2.7	0.003	0.03	0.03	0.03	0.25	3.9
**2euc**	2.5	P 21 21 21	P 41 21 2	2	1	0.08	3.1	0.001	0.03	0.02	0.02	0.74	---
**3buo**	2.6	P 1 21 1	P 21 21 2	2	2	0.06	1.0	0.000	0.03	0.02	0.02	---	7.1
**3g1v**	1.3	P 41	P 41 21 2	2	1	0.00	7.8	0.009	0.03	0.03	0.03	0.05	1.3
**2o7u**	2.8	C 1 2 1	P 31 2 1	3	3	0.03	1.0	0.003	0.03	0.03	0.02	0.37	7.2
**3c4n**	2.4	P 1 21 1	C 2 2 21	2	1	0.01	2.4	0.001	0.03	0.03	0.03	0.27	---
**2p7k**	3.3	P 61	P 61 2 2	2	1	0.00	3.4	0.002	0.03	0.03	0.03	0.37	---
**1km1**	1.6	P 1 21 1	C 2 2 21	2	1	0.00	6.3	0.005	0.03	0.03	0.03	0.12	---
**1qzx**	4.0	I 4	I 4 2 2	2	1	0.00	0.9	0.000	0.03	0.00	0.00	---	3.0
**3fmt**	3.0	R 3 :H	R 3 2 :H	2	2	0.00	0.8	0.001	0.03	0.01	0.00	1.83	2.7
**3gw7**	3.3	P 63	P 63 2 2	2	1	0.00	0.5	0.001	0.03	0.02	0.02	0.74	---
**2c82**	1.9	P 41	P 41 2 2	2	1	0.00	1.2	0.002	0.03	0.02	0.02	0.12	3.1
**2r1u**	1.5	P 31	P 31 2 1	2	1	0.00	6.3	0.006	0.03	0.03	0.03	---	---
**2z5i**	2.1	P 1 21 1	C 2 2 21	2	5	0.04	1.7	0.002	0.03	0.03	0.01	0.13	3.5
**2o96**	3.0	C 1 2 1	I 21 21 21	2	1	0.02	4.1	0.002	0.03	0.03	0.03	0.26	3.8
**2v6e**	3.2	P 41	P 41 2 2	2	1	0.00	0.9	0.001	0.03	0.03	0.03	0.45	8.7
**2gw4**	1.6	C 1 2 1	I 2 2 2	2	2	0.04	8.5	0.008	0.03	0.03	0.03	---	5.3
**1qma**	2.5	P 1 21 1	C 2 2 21	2	2	0.11	2.2	0.002	0.03	0.03	0.03	---	---
**1nt0**	2.7	P 1 21 1	C 2 2 21	2	1	0.10	1.0	0.001	0.03	0.02	0.02	0.48	5.6
**1y0v**	3.6	C 1 2 1	P 65 2 2	6	2	0.05	0.8	0.006	0.03	0.03	0.03	0.44	---
**1oog**	1.4	P 43	P 43 21 2	2	1	0.00	6.6	0.006	0.04	0.03	0.03	0.07	---
**1oki**	1.4	P 43	P 43 21 2	2	1	0.00	6.2	0.005	0.04	0.03	0.03	0.05	1.3
**3sxl**	2.7	P 1 21 1	P 65	3	1	0.03	1.9	0.000	0.04	0.03	0.03	0.50	---
**2ceu**	1.8	I 2 2 2	I 4 2 2	2	2	0.02	2.0	0.000	0.04	0.03	0.03	0.07	2.1
**3d5s**	2.3	P 41	P 41 21 2	2	2	0.00	2.2	0.001	0.04	0.03	0.03	---	3.5
**2f1v**	2.7	P 21 21 21	P 41 21 2	2	3	0.07	1.0	0.000	0.04	0.03	0.01	0.33	2.7
**3it3**	1.5	P 41	P 41 21 2	2	1	0.00	3.8	0.003	0.04	0.04	0.04	0.23	---
**1yq5**	2.0	P 21 3	P 41 3 2	2	1	0.00	1.8	0.000	0.04	0.03	0.03	---	1.0

PDB Code	Resolution (Å)	Published Symmetry	Candidate Symmetry	No. of Cosets	Chains / asu	δ (°)	R_{symop} (I^{calc}) (%)	φ(g_i)	Δr_{sym} (Å)	Δr_{asu} (Å)	Δr_{chain} (Å)	Coord. Error (Å)	R_{symop} (I^{obs}) (%)
**2rkg**	1.8	P 61	P 61 2 2	2	1	0.00	6.6	0.007	0.04	0.04	0.04	0.11	1.3
**2w0h**	3.0	P 41	P 41 21 2	2	1	0.00	3.9	0.003	0.04	0.03	0.03	0.32	5.1
**1pl6**	2.0	P 62	P 62 2 2	2	2	0.00	2.3	0.002	0.04	0.04	0.04	0.17	---
**3gw1**	2.4	P 1 21 1	P 21 21 2	2	1	0.10	3.2	0.001	0.04	0.02	0.02	0.45	13.3
**2j5y**	1.4	P 1 21 1	P 21 21 21	2	1	0.03	5.4	0.006	0.04	0.04	0.04	0.04	4.5
**1x1z**	1.4	P 1 21 1	C 2 2 21	2	1	0.00	5.5	0.003	0.04	0.04	0.04	0.09	---
**1rir**	2.9	P 32	P 32 2 1	2	2	0.00	3.7	0.003	0.04	0.03	0.02	---	11.4
**2i0j**	2.9	P 1 21 1	C 2 2 21	2	2	0.02	3.5	0.002	0.04	0.04	0.04	0.70	5.5
**2qqr**	1.8	I 21 3	I 41 3 2	2	1	0.00	2.6	0.001	0.04	0.04	0.04	0.07	2.6
**3gu3**	2.3	P 32	P 32 2 1	2	1	0.00	1.4	0.000	0.04	0.04	0.04	0.23	---
**3fvb**	1.8	F 2 3	F 4 3 2	2	1	0.00	4.0	0.002	0.04	0.04	0.04	0.07	1.7
**3icf**	2.3	P 32	P 32 2 1	2	1	0.00	2.6	0.001	0.04	0.04	0.04	0.23	2.0
**3f1r**	2.5	R 3 :H	R 3 2 :H	2	1	0.00	5.7	0.008	0.04	0.03	0.03	---	0.8
**3dbj**	2.9	P 3	P 63 2 2	4	2	0.00	3.7	0.002	0.04	0.03	0.03	---	6.4
**2g09**	2.1	P 32	P 62	2	1	0.00	3.9	0.002	0.04	0.04	0.04	0.10	---
**2g08**	2.4	P 32	P 62	2	1	0.00	4.7	0.003	0.04	0.04	0.04	0.19	---
**3i1e**	2.9	P 31	P 31 2 1	2	1	0.00	1.5	0.001	0.04	0.01	0.01	0.74	---
**3exa**	2.3	P 1	P 1 21 1	2	2	0.02	3.4	0.003	0.04	0.04	0.04	0.33	---
**2r1v**	1.7	P 31	P 31 2 1	2	1	0.00	8.6	0.009	0.04	0.04	0.04	---	---
**3bix**	1.8	P 32	P 65 2 2	4	1	0.00	4.4	0.004	0.04	0.04	0.04	0.22	3.7
**1zct**	2.6	P 64	P 64 2 2	2	1	0.00	1.1	0.000	0.04	0.04	0.04	0.45	1.0
**1kiu**	3.0	C 1 2 1	P 41 21 2	4	4	0.02	2.4	0.001	0.04	0.03	0.01	0.55	6.0
**3cae**	3.0	P 1 21 1	C 2 2 21	2	5	0.06	1.5	0.002	0.04	0.04	0.02	---	4.3
**3gup**	1.5	P 43	P 43 21 2	2	1	0.00	2.7	0.003	0.04	0.04	0.04	0.07	2.0
**2r55**	2.5	P 65	P 65 2 2	2	1	0.00	0.9	0.000	0.04	0.04	0.04	0.32	2.0
**2f67**	1.6	C 1 2 1	C 2 2 21	2	1	0.01	4.0	0.004	0.04	0.04	0.04	0.05	4.7
**2al4**	1.7	P 1 21 1	P 21 21 2	2	3	0.03	2.0	0.003	0.04	0.02	0.01	0.09	5.4
**3c6q**	2.3	P 1 21 1	P 21 21 21	2	2	0.10	3.0	0.003	0.04	0.04	0.03	---	7.7
**3dmo**	1.6	P 1 21 1	C 2 2 21	2	2	0.01	4.6	0.003	0.04	0.04	0.04	0.08	3.9
**2pyb**	2.6	R 3 :H	F 2 3	4	1	0.12	1.7	0.000	0.04	0.03	0.03	0.99	4.6
**3ef2**	1.8	P 3	P 63 2 2	4	1	0.00	4.1	0.003	0.04	0.04	0.04	0.06	---
**3iq2**	1.7	P 32	P 32 2 1	2	1	0.00	4.5	0.003	0.04	0.04	0.04	0.06	---
**2erg**	3.1	P 62	P 62 2 2	2	1	0.00	1.2	0.003	0.04	0.01	0.01	0.61	---
**2zz4**	1.7	P 1 21 1	C 2 2 21	2	1	0.01	4.7	0.003	0.04	0.04	0.04	0.11	4.0
**1oof**	1.5	P 43	P 43 21 2	2	1	0.00	6.3	0.007	0.04	0.04	0.04	0.08	---
**1xfy**	3.3	C 1 2 1	P 65 2 2	6	2	0.08	0.9	0.002	0.04	0.04	0.03	0.64	3.7
**3ccd**	1.0	P 1 21 1	P 21 21 2	2	1	0.05	9.2	0.011	0.04	0.04	0.04	0.02	3.3
**2p3d**	2.8	P 61	P 61 2 2	2	1	0.00	1.9	0.001	0.04	0.04	0.04	0.33	4.5
**2zz2**	1.5	P 1 21 1	C 2 2 21	2	1	0.01	6.1	0.004	0.04	0.04	0.04	0.04	2.5
**1hw1**	1.5	P 1 21 1	C 2 2 21	2	1	0.06	5.5	0.004	0.04	0.04	0.04	---	2.5
**3g18**	1.6	P 41	P 41 21 2	2	1	0.00	4.0	0.005	0.04	0.04	0.04	---	1.0
**1xfu**	3.4	C 1 2 1	P 65 2 2	6	2	0.08	1.0	0.002	0.04	0.03	0.03	0.69	3.0
**1yo3**	1.7	C 1 2 1	R 3 2 :H	3	1	0.01	5.6	0.004	0.04	0.04	0.04	0.06	1.7
**1fqx**	3.1	P 61	P 61 2 2	2	1	0.00	2.5	0.001	0.04	0.02	0.02	---	7.4
**2zxi**	2.3	P 1 21 1	P 21 21 2	2	2	0.00	5.0	0.003	0.04	0.04	0.04	0.41	3.3

PDB Code	Resolution (Å)	Published Symmetry	Candidate Symmetry	No. of Cosets	Chains / asu	δ (°)	R_{symop} (I^{calc}) (%)	φ(g_i)	Δr_{sym} (Å)	Δr_{asu} (Å)	Δr_{chain} (Å)	Coord. Error (Å)	R_{symop} (I^{obs}) (%)
**1pt5**	2.0	P 62	P 62 2 2	2	1	0.00	5.1	0.003	0.04	0.04	0.04	0.06	1.9
**3k8d**	1.9	P 31	P 31 2 1	2	2	0.00	3.7	0.003	0.04	0.04	0.04	0.22	---
**2qs2**	1.8	C 2 2 21	P 41 21 2	2	1	0.03	5.1	0.003	0.04	0.04	0.04	0.07	2.3
**2ixx**	2.5	P 3 2 1	P 63 2 2	2	1	0.00	2.8	0.001	0.04	0.04	0.04	0.13	4.0
**1iil**	2.3	P 1	P 1 21 1	2	4	0.01	5.9	0.004	0.04	0.04	0.04	---	---
**3fod**	1.4	P 1 21 1	P 21 21 21	2	4	0.01	5.6	0.009	0.04	0.04	0.03	0.07	5.2
**1w9s**	1.6	P 1	C 1 2 1	2	1	0.01	4.6	0.004	0.04	0.04	0.04	0.05	1.7
**1u8g**	2.2	P 61	P 61 2 2	2	1	0.00	1.9	0.001	0.04	0.04	0.04	---	2.3
**1t91**	1.9	P 1	C 1 2 1	2	2	0.18	8.4	0.016	0.04	0.04	0.04	---	4.6
**2q5c**	1.5	P 1	C 1 2 1	2	1	0.03	4.8	0.004	0.05	0.04	0.04	0.05	5.5
**2je1**	2.7	P 63	P 63 2 2	2	2	0.00	7.8	0.012	0.05	0.05	0.04	0.30	4.6
**1tc5**	1.9	P 2 2 21	P 43 2 2	2	2	0.01	5.1	0.002	0.05	0.05	0.04	0.13	4.6
**2p3b**	2.1	P 61	P 61 2 2	2	1	0.00	5.1	0.004	0.05	0.04	0.04	0.14	3.3
**2cu6**	2.0	I 2 3	I 4 3 2	2	1	0.00	5.9	0.004	0.05	0.05	0.05	0.14	0.9
**1b5e**	1.6	C 1 2 1	I 2 2 2	2	1	0.27	3.6	0.001	0.05	0.04	0.04	---	8.5
**1zlj**	2.0	P 1	P 1 21 1	2	4	0.14	5.4	0.005	0.05	0.04	0.04	0.11	3.9
**2g8s**	1.5	P 1 21 1	P 21 21 21	2	1	0.03	5.1	0.003	0.05	0.05	0.05	0.03	2.6
**2jc2**	2.5	P 65	P 65 2 2	2	2	0.00	2.5	0.001	0.05	0.05	0.04	0.29	2.4
**2p3c**	2.8	P 61	P 61 2 2	2	1	0.00	3.2	0.002	0.05	0.04	0.04	0.20	4.5
**2p3a**	1.8	P 61	P 61 2 2	2	1	0.00	3.5	0.003	0.05	0.05	0.05	0.11	1.8
**2i9b**	2.8	P 1 21 1	C 2 2 21	2	4	0.10	1.2	0.001	0.05	0.05	0.05	0.28	4.1
**2g0a**	2.4	P 32	P 62	2	1	0.00	4.7	0.003	0.05	0.05	0.05	---	---
**3gne**	1.2	P 1 21 1	C 2 2 21	2	1	0.01	5.7	0.005	0.05	0.05	0.05	---	---
**2g06**	2.2	P 32	P 62	2	1	0.00	4.9	0.003	0.05	0.05	0.05	0.14	---
**3c61**	1.8	P 32	P 32 2 1	2	2	0.00	4.9	0.005	0.05	0.05	0.05	0.07	---
**3cnr**	1.9	P 61	P 61 2 2	2	1	0.00	11.4	0.022	0.05	0.05	0.05	0.12	---
**3g1x**	1.6	P 41	P 41 21 2	2	1	0.00	7.0	0.008	0.05	0.05	0.05	0.10	1.5
**2r7i**	3.0	P 43	P 43 21 2	2	2	0.00	2.7	0.001	0.05	0.05	0.05	0.38	3.3
**2aqp**	1.3	C 1 2 1	I 2 2 2	2	1	0.08	6.0	0.003	0.05	0.05	0.05	---	3.2
**2d16**	1.7	P 3	P 63	2	2	0.00	6.3	0.003	0.05	0.05	0.05	0.24	2.0
**2zz1**	1.6	P 1 21 1	C 2 2 21	2	1	0.02	5.3	0.004	0.05	0.05	0.05	0.04	2.9
**1ev2**	2.2	P 1	P 1 21 1	2	4	0.02	8.6	0.010	0.05	0.05	0.05	---	---
**1klf**	2.8	C 1 2 1	P 41 21 2	4	4	0.02	1.8	0.001	0.05	0.03	0.01	0.51	3.2
**1ucf**	1.9	P 31	P 31 2 1	2	1	0.00	5.5	0.005	0.05	0.05	0.05	0.18	3.6
**2vhz**	2.0	P 21 3	P 43 3 2	2	1	0.00	2.9	0.001	0.05	0.05	0.05	0.10	2.9
**1b49**	2.3	C 1 2 1	I 2 2 2	2	1	0.03	3.5	0.001	0.05	0.05	0.05	---	5.7
**3ezq**	2.7	P 61	P 61 2 2	2	8	0.00	4.1	0.005	0.05	0.05	0.02	0.27	0.8
**2gr7**	2.3	C 1 2 1	P 3 2 1	3	2	0.09	3.8	0.003	0.05	0.05	0.05	0.15	4.8
**3h7n**	3.0	P 1	P 21 21 2	4	1	0.07	1.0	0.002	0.05	0.05	0.05	---	4.4
**3it0**	1.7	P 41	P 41 21 2	2	1	0.00	4.3	0.003	0.05	0.05	0.05	0.33	---
**2inv**	1.8	C 1 2 1	R 3 2 :H	3	1	0.00	5.3	0.007	0.05	0.05	0.05	---	2.4
**1hv4**	2.8	P 1	C 1 2 1	2	4	0.16	2.5	0.001	0.05	0.05	0.03	0.55	6.9
**2aen**	1.6	P 1	P 1 21 1	2	4	0.03	5.1	0.004	0.05	0.05	0.05	0.06	3.7
**1c03**	2.3	P 1	I 2 2 2	4	1	0.31	2.4	0.003	0.05	0.05	0.05	---	4.9
**1hm9**	1.8	R 3 :H	R 3 2 :H	2	1	0.00	3.3	0.004	0.05	0.05	0.05	0.23	3.0

PDB Code	Resolution (Å)	Published Symmetry	Candidate Symmetry	No. of Cosets	Chains / asu	δ (°)	R_{symop} (I^{calc}) (%)	φ(g_i)	Δr_{sym} (Å)	Δr_{asu} (Å)	Δr_{chain} (Å)	Coord. Error (Å)	R_{symop} (I^{obs}) (%)
**1r52**	2.9	P 1	I 2 2 2	4	1	0.04	4.2	0.002	0.05	0.05	0.05	0.27	3.5
**2f2t**	1.7	C 1 2 1	C 2 2 21	2	1	0.05	6.2	0.005	0.05	0.05	0.05	0.06	4.6
**1ww5**	2.2	P 1	C 2 2 21	4	1	0.14	4.2	0.004	0.05	0.05	0.05	0.46	4.4
**1b5d**	2.2	C 1 2 1	I 2 2 2	2	1	0.04	4.5	0.002	0.05	0.05	0.05	---	8.6
**2c57**	3.1	P 31	P 64 2 2	4	3	0.00	1.5	0.001	0.05	0.05	0.05	0.34	4.4
**1w9t**	1.6	P 1	C 1 2 1	2	1	0.04	4.8	0.003	0.05	0.05	0.05	0.06	2.5
**3hi8**	3.2	C 1 2 1	I 2 3	6	1	0.06	1.0	0.002	0.05	0.01	0.01	---	---
**1ukp**	2.1	P 1	P 21 21 21	4	1	0.23	3.8	0.003	0.05	0.05	0.05	0.25	9.2
**1s3m**	2.5	I 41	I 41 2 2	2	1	0.00	1.7	0.001	0.05	0.05	0.05	0.40	2.1
**1xqb**	2.9	P 21 21 2	P 42 21 2	2	1	0.22	4.7	0.002	0.05	0.05	0.05	0.49	3.8
**2p23**	1.8	P 3	P 63	2	1	0.00	8.8	0.008	0.05	0.05	0.05	---	1.6
**1et1**	0.9	P 65	P 65 2 2	2	1	0.00	5.6	0.010	0.05	0.05	0.05	---	---
**3foe**	4.0	P 32	P 62	2	2	0.00	5.1	0.003	0.05	0.05	0.05	0.68	4.0
**3fix**	2.3	P 1	P 1 21 1	2	2	0.10	3.0	0.002	0.05	0.05	0.05	0.23	4.7
**2bnk**	2.9	P 61	P 61 2 2	2	1	0.00	1.2	0.000	0.06	0.04	0.04	0.23	2.0
**1ov8**	1.9	P 65	P 65 2 2	2	2	0.00	4.1	0.003	0.06	0.06	0.05	0.09	2.7
**3hvd**	3.2	P 1	I 4	4	2	0.17	5.2	0.004	0.06	0.05	0.05	3.22	8.6
**3he8**	1.9	P 32 2 1	P 62 2 2	2	1	0.00	5.0	0.003	0.06	0.06	0.06	0.07	---
**1jd4**	2.7	I 4	I 4 2 2	2	1	0.00	3.5	0.003	0.06	0.06	0.06	---	---
**2uwb**	2.0	P 6	P 6 2 2	2	1	0.00	4.1	0.004	0.06	0.06	0.06	0.08	2.4
**2inu**	1.8	C 1 2 1	R 3 2 :H	3	1	0.02	5.6	0.007	0.06	0.06	0.06	---	2.7
**3cdf**	1.5	P 1 21 1	P 61	3	2	0.45	9.1	0.003	0.06	0.06	0.06	---	8.8
**1gus**	1.8	C 1 2 1	R 3 2 :H	3	2	0.30	5.5	0.006	0.06	0.06	0.06	0.09	3.0
**3kb1**	2.9	P 1 21 1	P 21 21 2	2	1	0.09	1.7	0.002	0.06	0.05	0.05	0.50	---
**2zz6**	1.7	P 1 21 1	C 2 2 21	2	1	0.01	5.3	0.003	0.06	0.06	0.06	0.10	2.4
**2vnk**	1.9	P 1 21 1	P 21 21 2	2	2	0.03	4.7	0.002	0.06	0.06	0.06	---	3.8
**3g01**	2.5	P 61	P 61 2 2	2	1	0.00	2.4	0.001	0.06	0.06	0.06	0.18	1.8
**1zwz**	1.9	P 31	P 31 2 1	2	1	0.00	7.9	0.007	0.06	0.06	0.06	0.09	1.4
**3e17**	1.8	P 1	C 1 2 1	2	1	0.00	8.8	0.008	0.06	0.06	0.06	---	2.0
**3cv2**	1.4	P 1 21 1	P 21 21 21	2	1	0.06	6.6	0.006	0.06	0.05	0.05	0.05	3.7
**2yzj**	1.7	P 1	R 3 :H	3	1	0.03	5.9	0.006	0.06	0.06	0.06	0.21	---
**2vax**	2.6	P 1 21 1	P 21 21 21	2	6	0.03	3.7	0.001	0.06	0.06	0.05	0.24	3.0
**1vhj**	2.2	P 1 21 1	C 2 2 21	2	3	0.06	5.5	0.004	0.06	0.06	0.06	---	3.7
**3czy**	1.5	P 1 21 1	P 21 21 2	2	1	0.00	6.6	0.005	0.06	0.06	0.06	0.06	2.7
**2wqk**	1.5	P 32 2 1	P 62 2 2	2	1	0.00	4.9	0.002	0.06	0.06	0.06	0.04	---
**1smq**	3.1	P 1	C 1 2 1	2	2	0.08	4.5	0.007	0.06	0.06	0.04	---	---
**1u9d**	1.7	P 3	P 63	2	1	0.00	6.1	0.004	0.06	0.06	0.06	0.10	2.3
**2dc4**	1.7	P 32	P 32 2 1	2	1	0.00	8.2	0.010	0.06	0.06	0.06	0.18	1.9
**1qun**	2.8	C 1 2 1	P 41 21 2	4	4	0.07	1.3	0.000	0.06	0.06	0.06	---	---
**3bj6**	2.0	P 21 21 21	P 41 21 2	2	1	0.08	6.6	0.004	0.06	0.06	0.06	0.09	2.7
**3b6x**	2.0	P 43	P 43 21 2	2	1	0.00	7.3	0.010	0.06	0.06	0.06	0.11	1.0
**2iwg**	2.4	P 61	P 61 2 2	2	2	0.00	1.3	0.002	0.06	0.06	0.06	0.20	3.2
**1yfl**	3.1	P 1 21 1	P 43	2	2	0.02	2.2	0.000	0.06	0.06	0.06	0.36	---
**3it1**	1.7	P 41	P 41 21 2	2	1	0.00	3.9	0.003	0.06	0.06	0.06	0.25	---
**2w2a**	1.4	P 43	P 43 21 2	2	1	0.00	6.2	0.007	0.06	0.06	0.06	0.04	---

PDB Code	Resolution (Å)	Published Symmetry	Candidate Symmetry	No. of Cosets	Chains / asu	δ (°)	R_{symop} (I^{calc}) (%)	φ(g_i)	Δr_{sym} (Å)	Δr_{asu} (Å)	Δr_{chain} (Å)	Coord. Error (Å)	R_{symop} (I^{obs}) (%)
**3it2**	1.8	P 41	P 41 21 2	2	1	0.00	4.0	0.004	0.06	0.06	0.06	0.38	---
**3d5r**	2.1	P 41	P 41 21 2	2	2	0.00	2.8	0.001	0.06	0.06	0.06	---	4.3
**2w2b**	1.4	P 43	P 43 21 2	2	1	0.00	5.1	0.003	0.06	0.06	0.06	0.04	---
**2vhv**	2.8	P 21 3	P 43 3 2	2	1	0.00	4.2	0.002	0.06	0.06	0.06	0.25	3.0
**2zz3**	1.8	P 1 21 1	C 2 2 21	2	1	0.00	5.1	0.004	0.06	0.06	0.06	0.13	2.8
**3g1y**	1.4	P 41	P 41 21 2	2	1	0.00	5.0	0.005	0.06	0.06	0.06	0.23	1.3
**2g07**	2.3	P 32	P 62	2	1	0.00	5.4	0.004	0.06	0.06	0.06	0.18	---
**2pp1**	2.2	P 2 2 2	P 4 2 2	2	3	0.01	1.6	0.000	0.06	0.06	0.06	0.29	5.6
**1xai**	2.3	P 43	P 43 21 2	2	1	0.00	3.4	0.003	0.06	0.06	0.06	0.56	1.9
**3hee**	2.0	P 32 2 1	P 62 2 2	2	1	0.00	4.0	0.002	0.06	0.06	0.06	0.09	---
**3fdq**	1.8	P 1 21 1	C 2 2 21	2	1	0.03	1.8	0.001	0.06	0.00	0.00	---	5.0
**3g89**	1.5	P 61	P 61 2 2	2	1	0.00	6.1	0.007	0.06	0.06	0.06	0.23	3.3
**3c1v**	1.5	P 32	P 32 2 1	2	2	0.00	8.0	0.010	0.06	0.06	0.06	0.05	3.5
**2f64**	1.6	C 1 2 1	C 2 2 21	2	1	0.01	6.8	0.005	0.06	0.06	0.06	0.04	4.0
**2v5r**	3.0	C 1 2 1	I 2 2 2	2	1	0.06	2.3	0.001	0.06	0.06	0.06	---	6.2
**2ddd**	1.6	C 1 2 1	I 2 2 2	2	1	0.00	5.9	0.003	0.06	0.06	0.06	---	---
**2c91**	2.3	P 1	C 1 2 1	2	5	0.02	5.4	0.004	0.06	0.06	0.06	0.14	6.8
**1f5v**	1.7	P 1	C 1 2 1	2	1	0.37	3.8	0.002	0.06	0.06	0.06	---	5.0
**1bqa**	2.1	P 1 21 1	C 2 2 21	2	1	0.07	4.3	0.002	0.06	0.06	0.06	---	---
**3g88**	1.9	P 61	P 61 2 2	2	1	0.00	6.3	0.008	0.06	0.06	0.06	0.21	2.1
**1vhw**	1.5	P 1 21 1	C 2 2 21	2	3	0.03	5.7	0.005	0.07	0.07	0.06	---	3.0
**2g7r**	2.7	C 1 2 1	I 2 2 2	2	1	0.00	2.7	0.001	0.07	0.06	0.06	0.28	5.1
**2o95**	2.0	C 1 2 1	I 21 21 21	2	1	0.00	5.5	0.003	0.07	0.06	0.06	0.10	2.2
**2bdu**	2.4	P 32	P 62	2	1	0.00	4.1	0.002	0.07	0.07	0.07	0.15	---
**2ddr**	1.4	P 1	C 1 2 1	2	2	0.03	9.0	0.019	0.07	0.06	0.06	0.20	3.3
**2vav**	2.5	P 1 21 1	P 21 21 21	2	6	0.03	4.0	0.002	0.07	0.06	0.05	0.21	4.4
**1ybt**	2.3	P 1 2 1	P 21 21 2	2	2	0.00	5.0	0.002	0.07	0.07	0.06	0.39	2.5
**3dca**	3.4	R 3 :H	I 21 3	4	1	0.16	8.0	0.010	0.07	0.05	0.05	---	5.9
**3f98**	1.7	C 2 2 21	P 61 2 2	3	1	0.01	4.5	0.002	0.07	0.07	0.07	0.06	---
**3fg7**	2.0	P 31	P 61	2	1	0.00	2.0	0.000	0.07	0.06	0.06	0.12	2.3
**2d3e**	2.6	P 1	C 1 2 1	2	2	0.20	6.9	0.010	0.07	0.06	0.04	0.60	2.7
**3cff**	1.8	C 2 2 21	P 41 2 2	2	4	0.03	5.7	0.003	0.07	0.07	0.07	0.09	3.0
**1w6x**	2.0	P 1 21 1	C 2 2 21	2	1	0.18	5.8	0.006	0.07	0.06	0.06	0.47	6.0
**2oqy**	2.0	P 1	I 4	4	2	0.11	2.7	0.005	0.07	0.07	0.06	0.28	4.2
**1jt7**	1.7	C 1 2 1	C 2 2 21	2	2	0.00	4.5	0.004	0.07	0.07	0.07	---	2.6
**3eo0**	1.8	P 1 21 1	C 2 2 21	2	2	0.17	4.5	0.004	0.07	0.07	0.07	0.06	5.5
**1jtc**	1.7	C 1 2 1	C 2 2 21	2	2	0.02	5.2	0.005	0.07	0.07	0.07	---	3.9
**3h0l**	2.3	P 1	P 1 21 1	2	12	0.09	4.9	0.004	0.07	0.07	0.05	0.26	4.8
**2w41**	2.4	P 1 21 1	P 21 21 2	2	1	0.25	2.6	0.001	0.07	0.07	0.07	0.22	3.6
**2ddc**	1.6	C 1 2 1	I 2 2 2	2	1	0.01	7.0	0.006	0.07	0.07	0.07	---	---
**3bdw**	2.5	P 1 21 1	P 21 21 2	2	2	0.28	3.3	0.001	0.07	0.06	0.05	0.28	---
**2h11**	1.9	P 42	P 42 21 2	2	1	0.00	4.7	0.004	0.07	0.07	0.07	0.19	---
**1r0m**	1.3	P 4	P 4 21 2	2	2	0.00	7.5	0.007	0.07	0.07	0.07	0.02	2.2
**1z9o**	1.9	P 1	P 31 2 1	6	2	0.04	4.4	0.005	0.07	0.07	0.07	0.15	---
**3d0e**	2.0	P 32	P 65	2	1	0.00	6.5	0.004	0.07	0.07	0.07	0.14	3.9

PDB Code	Resolution (Å)	Published Symmetry	Candidate Symmetry	No. of Cosets	Chains / asu	δ (°)	R_{symop} (I^{calc}) (%)	φ(g_i)	Δr_{sym} (Å)	Δr_{asu} (Å)	Δr_{chain} (Å)	Coord. Error (Å)	R_{symop} (I^{obs}) (%)
**1oe8**	1.6	P 1 21 1	C 2 2 21	2	1	0.06	7.0	0.005	0.07	0.07	0.07	0.10	3.4
**3cn9**	2.1	P 21 21 2	P 4 21 2	2	1	0.04	4.0	0.002	0.07	0.07	0.07	0.24	3.2
**1xfz**	3.2	C 1 2 1	P 65 2 2	6	2	0.17	1.6	0.004	0.07	0.06	0.06	0.73	4.1
**2e6y**	1.6	P 1 21 1	C 2 2 21	2	1	0.01	6.1	0.004	0.07	0.07	0.07	0.10	1.7
**2bkx**	1.4	P 1 21 1	P 21 21 21	2	1	0.06	8.1	0.006	0.07	0.04	0.04	0.03	5.2
**2zad**	1.6	P 4	P 4 21 2	2	2	0.00	7.3	0.008	0.07	0.07	0.07	0.05	5.8
**2gf0**	1.9	P 1 21 1	P 21 21 21	2	2	0.02	7.1	0.008	0.07	0.07	0.07	0.09	5.7
**5pgm**	2.1	P 1 21 1	P 21 21 2	2	4	0.15	2.8	0.002	0.07	0.07	0.03	---	3.9
**3fgq**	2.1	P 1	P 1 21 1	2	1	0.11	3.3	0.003	0.07	0.07	0.07	0.31	---
**2idf**	2.2	P 61	P 61 2 2	2	1	0.00	2.7	0.003	0.07	0.07	0.07	---	---
**1azd**	3.0	P 21 21 21	P 43 21 2	2	2	0.29	3.2	0.001	0.07	0.07	0.07	---	---
**3fzz**	2.5	P 61	P 61 2 2	2	1	0.00	2.6	0.002	0.07	0.07	0.07	0.23	1.9
**2ac7**	1.7	P 63	P 63 2 2	2	1	0.00	7.2	0.006	0.07	0.07	0.07	0.05	3.0
**1mq8**	3.3	P 1	C 1 2 1	2	2	0.08	2.2	0.001	0.07	0.07	0.07	0.90	7.7
**1ww4**	2.3	P 1	C 2 2 21	4	1	0.28	5.0	0.004	0.07	0.07	0.07	0.52	6.9
**1m0b**	2.5	P 61	P 61 2 2	2	1	0.00	2.6	0.001	0.07	0.07	0.07	0.25	3.3
**3guw**	3.2	P 1	P 1 21 1	2	2	0.28	2.3	0.002	0.08	0.07	0.07	0.76	---
**2giz**	1.7	P 1 21 1	P 21 21 2	2	1	0.18	5.7	0.004	0.08	0.07	0.07	---	2.4
**1xpm**	1.6	P 1 21 1	P 21 21 2	2	2	0.08	4.6	0.003	0.08	0.06	0.06	---	4.7
**1hqq**	1.7	P 1 21 1	C 2 2 21	2	4	0.00	4.9	0.002	0.08	0.07	0.07	0.12	6.6
**1knz**	2.5	P 1	P 1 21 1	2	4	0.01	7.2	0.006	0.08	0.07	0.07	---	---
**1m2x**	1.5	R 3 :H	I 2 3	4	1	0.02	6.3	0.003	0.08	0.07	0.07	0.08	7.8
**2pzv**	1.2	P 1	C 2 2 21	4	1	0.02	7.1	0.008	0.08	0.08	0.08	0.04	3.5
**3hk2**	2.8	P 21 21 21	P 43 21 2	2	1	0.16	3.2	0.001	0.08	0.07	0.07	0.36	---
**1ynf**	1.9	P 1	C 1 2 1	2	3	0.26	8.3	0.009	0.08	0.08	0.08	0.35	6.7
**2jdj**	2.0	P 32 1 2	P 62 2 2	2	1	0.00	5.8	0.005	0.08	0.08	0.08	0.11	0.9
**1gwc**	2.2	C 2 2 21	P 61 2 2	3	1	0.00	6.4	0.004	0.08	0.07	0.07	0.18	---
**1tw7**	1.3	P 41	P 41 21 2	2	1	0.00	4.6	0.004	0.08	0.08	0.08	---	1.6
**1ob5**	3.1	C 1 2 1	R 3 2 :H	3	1	0.23	1.2	0.002	0.08	0.06	0.06	0.60	3.6
**2r1t**	1.7	P 31	P 31 2 1	2	1	0.00	7.8	0.009	0.08	0.08	0.08	---	---
**3fgx**	2.9	P 43	P 43 21 2	2	1	0.00	4.1	0.005	0.08	0.04	0.04	0.39	4.9
**3bfo**	1.1	P 1	P 1 21 1	2	2	0.02	7.2	0.007	0.08	0.08	0.08	0.03	3.5
**2ag5**	1.8	P 1	C 1 2 1	2	2	0.09	7.2	0.007	0.08	0.08	0.08	0.09	5.3
**2oxl**	1.8	C 2 2 21	P 43 2 2	2	1	0.02	5.5	0.003	0.08	0.08	0.08	0.10	---
**1jlv**	1.8	P 43	P 43 21 2	2	3	0.00	6.0	0.004	0.08	0.08	0.08	0.15	3.6
**1om9**	2.5	P 31 2 1	P 64 2 2	2	2	0.00	8.0	0.009	0.08	0.08	0.07	0.23	---
**3fj5**	1.6	P 65	P 65 2 2	2	1	0.00	4.2	0.003	0.08	0.08	0.08	0.06	1.9
**3b7a**	1.9	P 43	P 43 21 2	2	1	0.00	8.3	0.011	0.08	0.08	0.08	0.11	3.0
**1zbl**	2.2	P 1	C 1 2 1	2	1	0.13	4.0	0.002	0.08	0.08	0.08	0.42	3.8
**2cz8**	1.5	P 3	P 63	2	4	0.00	6.5	0.004	0.08	0.08	0.08	0.08	2.9
**1xfw**	3.4	C 1 2 1	P 65 2 2	6	2	0.22	2.0	0.005	0.08	0.06	0.06	0.70	4.6
**3hjb**	1.5	P 1 21 1	P 21 21 21	2	2	0.21	6.2	0.004	0.08	0.07	0.06	0.03	5.4
**2qb1**	2.6	P 31	P 64	2	1	0.00	2.2	0.000	0.08	0.06	0.06	0.27	2.9
**3bp1**	1.5	P 1	C 1 2 1	2	2	0.06	6.6	0.006	0.08	0.08	0.08	0.05	2.6
**2on5**	1.9	P 1 21 1	P 21 21 2	2	4	0.02	7.4	0.007	0.08	0.08	0.08	0.07	---

PDB Code	Resolution (Å)	Published Symmetry	Candidate Symmetry	No. of Cosets	Chains / asu	δ (°)	R_{symop} (I^{calc}) (%)	$\varphi(g_i)$	Δr_{sym} (Å)	Δr_{asu} (Å)	Δr_{chain} (Å)	Coord. Error (Å)	R_{symop} (I^{obs}) (%)
**2rh0**	1.9	P 1	C 1 2 1	2	2	0.16	6.1	0.005	0.08	0.08	0.08	0.14	---
**1sc0**	1.7	C 1 2 1	F 2 2 2	2	1	0.02	6.9	0.005	0.08	0.08	0.08	0.14	---
**3h0m**	2.8	P 1	P 1 21 1	2	12	0.11	4.2	0.003	0.08	0.08	0.07	0.41	8.3
**2gd0**	1.7	C 1 2 1	I 2 2 2	2	2	0.01	4.0	0.002	0.08	0.08	0.07	0.11	2.9
**1je1**	1.8	P 1 21 1	C 2 2 21	2	3	0.00	9.0	0.008	0.08	0.08	0.08	---	---
**1nf7**	2.7	I 4	I 4 2 2	2	1	0.00	5.5	0.005	0.08	0.08	0.08	0.48	9.1
**1oe7**	1.8	P 1 21 1	C 2 2 21	2	1	0.08	8.1	0.008	0.08	0.08	0.08	0.11	1.1
**3hjo**	1.9	C 1 2 1	C 2 2 21	2	1	0.07	6.5	0.005	0.08	0.08	0.08	0.09	---
**2qg4**	2.1	R 3 :H	R 3 2 :H	2	4	0.00	4.9	0.004	0.08	0.08	0.08	0.12	---
**1rby**	2.1	P 31	P 31 2 1	2	2	0.00	4.0	0.003	0.08	0.08	0.07	0.11	7.1
**2z5c**	2.9	P 21 21 2	P 4 21 2	2	3	0.18	1.5	0.000	0.08	0.04	0.04	0.28	3.9
**3b6a**	3.1	P 1	P 21 21 21	4	2	0.46	3.1	0.003	0.08	0.08	0.08	---	7.6
**3eik**	1.9	R 3 :H	R 3 2 :H	2	1	0.00	7.1	0.008	0.08	0.08	0.08	0.31	---
**1wpu**	1.5	R 3 :H	R 3 2 :H	2	1	0.00	5.5	0.003	0.08	0.08	0.08	0.12	1.2
**2wmy**	2.2	P 1 21 1	P 21 21 2	2	4	0.01	5.7	0.004	0.08	0.08	0.08	0.77	3.7
**2ere**	3.0	P 62	P 62 2 2	2	1	0.00	1.3	0.001	0.08	0.01	0.01	0.67	---
**1pl8**	1.9	P 62	P 62 2 2	2	2	0.00	3.7	0.003	0.08	0.07	0.06	0.24	---
**1r0e**	2.2	P 21 21 21	P 43 21 2	2	1	0.04	4.9	0.003	0.08	0.08	0.08	0.13	---
**1t14**	1.9	P 43	P 43 21 2	2	1	0.00	6.8	0.009	0.08	0.08	0.08	---	---
**1izi**	2.1	P 61	P 61 2 2	2	1	0.00	5.5	0.006	0.08	0.08	0.08	0.16	---
**1zro**	2.3	C 2 2 21	P 41 2 2	2	1	0.18	1.8	0.000	0.08	0.06	0.06	0.13	3.5
**2axp**	2.5	C 1 2 1	F 2 2 2	2	1	0.15	3.2	0.002	0.08	0.08	0.08	0.59	---
**3k2j**	2.2	P 65	P 65 2 2	2	1	0.00	5.6	0.006	0.08	0.08	0.08	0.19	---
**3gyk**	1.8	P 1 21 1	C 2 2 21	2	2	0.04	6.5	0.004	0.09	0.08	0.08	0.07	2.4
**1jds**	1.8	P 1 21 1	C 2 2 21	2	3	0.39	10.3	0.012	0.09	0.08	0.08	---	---
**1h6d**	2.1	P 1 21 1	P 21 21 2	2	6	0.00	6.0	0.004	0.09	0.09	0.08	0.28	4.8
**3fmp**	3.2	P 1 21 1	P 21 21 21	2	2	0.14	2.2	0.001	0.09	0.08	0.08	0.37	5.3
**3cs2**	2.0	P 1	P 1 21 1	2	2	0.06	5.6	0.005	0.09	0.09	0.08	0.11	4.8
**3c3d**	2.5	P 32	P 65	2	2	0.00	3.5	0.002	0.09	0.09	0.09	0.34	---
**2w1v**	1.5	P 32	P 32 2 1	2	1	0.00	9.0	0.008	0.09	0.09	0.09	0.04	1.2
**1s7v**	2.2	C 1 2 1	I 2 2 2	2	3	0.05	5.4	0.003	0.09	0.08	0.08	0.19	4.2
**2qyh**	2.6	P 21 21 21	P 41 21 2	2	2	0.04	3.5	0.001	0.09	0.09	0.09	0.39	2.1
**1ffp**	2.6	P 1	C 1 2 1	2	3	0.17	3.6	0.001	0.09	0.09	0.09	0.52	9.0
**1te2**	1.8	P 1 21 1	C 2 2 21	2	1	0.00	8.3	0.007	0.09	0.09	0.09	---	3.1
**2o4q**	2.0	P 1	P 1 21 1	2	2	0.06	5.4	0.005	0.09	0.09	0.09	0.11	4.8
**2p1r**	2.5	P 1 21 1	C 2 2 21	2	2	0.00	6.1	0.004	0.09	0.09	0.09	0.52	3.7
**2dds**	1.8	P 1	C 1 2 1	2	2	0.05	6.7	0.006	0.09	0.09	0.09	0.17	2.2
**2clb**	2.4	I 21 3	I 41 3 2	2	4	0.00	4.1	0.002	0.09	0.09	0.09	0.19	1.9
**3g8a**	2.1	P 61	P 61 2 2	2	3	0.00	6.2	0.007	0.09	0.09	0.09	0.29	3.4
**3ehr**	2.0	P 1	C 1 2 1	2	1	0.08	10.4	0.012	0.09	0.09	0.09	0.11	3.4
**2f4p**	1.9	P 65	P 65 2 2	2	2	0.00	6.1	0.007	0.09	0.09	0.09	0.10	---
**1xfv**	3.4	C 1 2 1	P 65 2 2	6	2	0.22	2.0	0.001	0.09	0.07	0.07	0.69	5.0
**2p4u**	1.9	P 1 21 1	C 2 2 21	2	2	0.01	5.0	0.004	0.09	0.09	0.09	0.11	4.8
**2j4y**	3.4	P 31	P 64	2	1	0.00	3.1	0.001	0.09	0.09	0.09	0.88	10.9
**2g75**	2.3	P 31	P 31 2 1	2	2	0.00	5.7	0.006	0.09	0.09	0.09	0.47	3.0

**PDB Code**	**Resolution (Å)**	**Published Symmetry**	**Candidate Symmetry**	**No. of Cosets**	**Chains / asu**	**δ (°)**	R_{symop} (I^{calc}) **(%)**	φ(g_i)	Δr_{sym} **(Å)**	Δr_{asu} **(Å)**	Δr_{chain} **(Å)**	**Coord. Error (Å)**	R_{symop} (I^{obs}) **(%)**
**1zgs**	2.5	P 1 21 1	C 2 2 21	2	1	0.05	3.2	0.001	0.09	0.09	0.09	---	1.8
**1f1g**	1.4	P 1	R 3 2 :H	6	1	0.24	8.4	0.006	0.09	0.07	0.07	---	9.5
**2ayb**	3.2	P 61	P 61 2 2	2	1	0.00	2.6	0.001	0.09	0.09	0.09	0.40	---
**1oar**	2.2	P 21 21 21	P 41 21 2	2	4	0.03	7.8	0.005	0.09	0.08	0.02	0.15	2.2
**2pbf**	2.0	P 32	P 32 2 1	2	1	0.00	6.5	0.008	0.09	0.09	0.09	0.17	2.1
**2f3m**	2.7	P 1	C 1 2 1	2	3	0.07	4.4	0.003	0.09	0.09	0.08	0.38	5.7
**3cfh**	1.8	C 2 2 21	P 41 2 2	2	4	0.02	6.0	0.004	0.09	0.09	0.09	0.09	3.3
**1j1a**	2.2	P 31	P 31 2 1	2	1	0.00	3.6	0.002	0.09	0.09	0.09	0.40	2.6
**3ekt**	2.0	P 61	P 61 2 2	2	2	0.00	5.8	0.004	0.09	0.09	0.09	0.13	---
**1e6c**	1.8	P 1 21 1	C 2 2 21	2	1	0.04	3.6	0.003	0.09	0.09	0.09	---	---
**2vh9**	2.1	P 31	P 31 2 1	2	1	0.00	5.3	0.004	0.09	0.09	0.09	0.10	4.6
**2ddt**	1.8	P 1	C 1 2 1	2	1	0.06	6.2	0.003	0.09	0.08	0.08	0.09	5.1
**3es8**	2.2	P 1	I 4	4	2	0.14	3.4	0.002	0.09	0.05	0.04	0.52	8.5
**1ww6**	2.2	P 1	C 2 2 21	4	1	0.17	4.5	0.004	0.09	0.08	0.08	0.43	3.5
**1uko**	2.1	P 1	P 21 21 21	4	1	0.14	7.3	0.005	0.09	0.09	0.09	0.25	13.9
**1aaq**	2.5	P 61	P 61 2 2	2	1	0.00	3.9	0.003	0.09	0.09	0.09	---	---
**1pl7**	2.2	P 62	P 62 2 2	2	2	0.00	3.8	0.002	0.09	0.07	0.07	0.26	---
**2q63**	2.2	P 61	P 61 2 2	2	1	0.00	3.8	0.003	0.09	0.09	0.09	0.20	1.5
**2gf7**	2.2	R 3 2 :H	I 41 3 2	4	1	0.01	8.6	0.008	0.09	0.09	0.09	---	3.6
**3cxg**	2.0	P 2 3	P 42 3 2	2	1	0.00	7.4	0.007	0.09	0.09	0.09	0.12	---
**2a9w**	1.6	R 3 :H	I 21 3	4	1	0.03	6.6	0.006	0.09	0.09	0.09	0.23	3.6
**3gve**	1.2	P 1 21 1	P 21 21 2	2	1	0.06	6.5	0.006	0.09	0.07	0.07	0.12	5.8
**3eut**	2.0	P 1 21 1	P 21 21 21	2	2	0.10	5.9	0.004	0.09	0.09	0.09	0.11	3.3
**2fyx**	1.9	P 32	P 32 2 1	2	1	0.00	4.5	0.005	0.09	0.09	0.09	0.07	---
**2guj**	3.0	P 32	P 32 2 1	2	1	0.00	7.7	0.008	0.09	0.06	0.06	0.94	---
**1u0q**	1.6	P 32 2 1	P 65 2 2	2	1	0.00	10.2	0.009	0.09	0.09	0.09	---	---
**3g1s**	1.4	P 41	P 41 21 2	2	1	0.00	7.3	0.008	0.09	0.09	0.09	0.13	1.3
**1y6o**	2.0	P 1 21 1	C 2 2 21	2	1	0.03	6.7	0.006	0.09	0.09	0.09	0.24	3.5
**2dsa**	2.1	R 3 :H	R 3 2 :H	2	2	0.00	3.9	0.004	0.09	0.09	0.09	0.14	1.9
**3imd**	2.0	P 21 21 21	P 43 21 2	2	1	0.01	9.0	0.007	0.09	0.09	0.09	---	---
**2gdf**	2.4	P 32	P 32 2 1	2	2	0.00	2.8	0.001	0.10	0.07	0.06	---	---
**2h6l**	2.0	P 1	R 3 :H	3	1	0.07	6.4	0.007	0.10	0.09	0.09	0.22	---
**2vj4**	2.5	P 2 2 2	P 42 2 2	2	1	0.02	2.9	0.001	0.10	0.09	0.09	---	3.3
**3cse**	1.6	P 41	P 41 21 2	2	1	0.00	7.8	0.011	0.10	0.10	0.10	0.07	---
**1b8z**	1.6	P 43	P 43 21 2	2	1	0.00	8.3	0.013	0.10	0.09	0.09	---	1.5
**1v40**	1.9	P 1	P 1 21 1	2	2	0.02	7.1	0.007	0.10	0.10	0.10	0.21	3.3
**1les**	1.9	P 1 21 1	C 2 2 21	2	2	0.01	7.0	0.007	0.10	0.10	0.10	---	7.4
**2p6h**	2.0	P 3	P 3 2 1	2	1	0.00	5.1	0.003	0.10	0.09	0.09	0.25	0.9
**2jk6**	3.0	P 41	P 41 21 2	2	1	0.00	3.7	0.002	0.10	0.10	0.10	0.26	3.1
**1d01**	2.0	P 1 21 1	P 63	3	3	0.00	4.2	0.002	0.10	0.10	0.10	---	4.8
**2bii**	1.7	C 2 2 21	P 41 21 2	2	1	0.17	5.8	0.003	0.10	0.08	0.08	0.06	8.4
**2og9**	1.9	I 4	I 4 2 2	2	1	0.00	7.0	0.007	0.10	0.10	0.10	0.18	1.7
**3dwm**	2.7	C 1 2 1	I 2 2 2	2	1	0.10	5.1	0.005	0.10	0.10	0.10	0.26	4.6
**1idp**	1.4	P 1 21 1	P 63	3	1	0.03	6.6	0.004	0.10	0.10	0.10	---	---
**2e0m**	1.7	I 41	I 41 2 2	2	1	0.00	6.9	0.007	0.10	0.10	0.10	0.06	1.6

PDB Code	Resolution (Å)	Published Symmetry	Candidate Symmetry	No. of Cosets	Chains / asu	δ (°)	R_{symop} (I^{calc}) (%)	φ(g_i)	Δr_{sym} (Å)	Δr_{asu} (Å)	Δr_{chain} (Å)	Coord. Error (Å)	R_{symop} (I^{obs}) (%)
**2vvr**	2.1	C 1 2 1	P 32 2 1	3	2	0.11	6.2	0.005	0.10	0.10	0.10	0.13	9.0
**1ros**	2.0	R 3 :H	R 3 2 :H	2	1	0.00	9.4	0.010	0.10	0.10	0.10	---	4.9
**1ffn**	2.7	P 1	C 1 2 1	2	3	0.04	3.8	0.002	0.10	0.10	0.10	0.64	10.5
**2p6c**	2.0	R 3 :H	R 3 2 :H	2	1	0.00	5.9	0.004	0.10	0.10	0.10	0.15	1.2
**2p91**	2.0	P 21 21 21	P 43 21 2	2	2	0.09	9.7	0.008	0.10	0.08	0.08	---	3.6
**3fof**	4.0	P 32	P 62	2	2	0.00	5.4	0.003	0.10	0.10	0.10	0.60	---
**2a8y**	1.5	P 1	C 1 2 1	2	6	0.04	5.8	0.004	0.10	0.10	0.10	0.12	4.6
**2fkp**	2.0	P 4	P 4 21 2	2	2	0.00	7.5	0.008	0.10	0.10	0.10	0.11	3.7
**2pym**	1.9	P 61	P 61 2 2	2	1	0.00	5.7	0.007	0.10	0.10	0.10	0.14	1.2
**1gut**	1.5	C 1 2 1	R 3 2 :H	3	2	0.01	9.5	0.007	0.10	0.10	0.10	0.06	6.1
**2fdd**	1.6	P 41	P 41 21 2	2	1	0.00	8.4	0.009	0.10	0.10	0.10	---	1.2
**1xaj**	2.4	P 43	P 43 21 2	2	1	0.00	4.0	0.004	0.10	0.10	0.10	0.53	3.2
**2r89**	3.4	C 2 2 21	P 41 21 2	2	1	0.06	1.6	0.000	0.10	0.10	0.10	0.40	3.3
**2pdr**	1.7	P 1 21 1	P 21 21 21	2	2	0.06	7.7	0.009	0.10	0.10	0.10	---	6.2
**2ywv**	1.8	P 1	C 1 2 1	2	1	0.02	8.1	0.010	0.10	0.10	0.10	0.15	2.5
**1t8b**	3.2	P 32	P 32 2 1	2	1	0.00	3.1	0.008	0.10	0.09	0.09	0.53	2.4
**3clc**	2.8	P 65	P 65 2 2	2	2	0.00	2.3	0.002	0.10	0.07	0.06	0.25	3.7
**3hm3**	2.0	P 21 3	P 43 3 2	2	2	0.00	5.8	0.005	0.10	0.10	0.10	0.11	0.6
**1u8t**	1.5	P 1	C 1 2 1	2	3	0.10	8.8	0.009	0.10	0.10	0.10	---	3.1
**1ffo**	2.7	P 1	C 1 2 1	2	3	0.09	3.4	0.002	0.10	0.10	0.10	0.52	10.2
**1rl8**	2.0	P 61	P 61 2 2	2	1	0.00	4.1	0.005	0.10	0.10	0.10	0.40	1.1
**3k2c**	1.9	P 1	P 1 21 1	2	2	0.02	5.4	0.003	0.10	0.10	0.10	0.10	---
**1nfb**	2.9	I 4	I 4 2 2	2	1	0.00	5.7	0.006	0.11	0.08	0.08	0.53	7.0
**2vw5**	1.9	C 1 2 1	C 2 2 21	2	2	0.02	9.6	0.009	0.11	0.10	0.10	0.08	5.5
**3cfa**	1.8	C 2 2 21	P 41 2 2	2	4	0.05	7.7	0.006	0.11	0.10	0.10	0.08	3.8
**2ctz**	2.6	I 41	I 41 2 2	2	1	0.00	4.2	0.003	0.11	0.11	0.11	0.12	---
**1xah**	2.2	P 43	P 43 21 2	2	1	0.00	6.3	0.010	0.11	0.10	0.10	0.39	1.9
**3hqm**	1.7	P 1	C 1 2 1	2	2	0.44	10.3	0.010	0.11	0.09	0.09	0.07	---
**2hz5**	2.1	P 61	P 61 2 2	2	1	0.00	10.3	0.022	0.11	0.11	0.11	0.09	1.2
**2zgz**	2.2	P 41	P 41 2 2	2	1	0.00	3.0	0.003	0.11	0.11	0.11	0.28	1.6
**2g2x**	2.3	C 1 2 1	P 32 2 1	3	1	0.00	7.1	0.005	0.11	0.11	0.11	0.24	1.4
**2hms**	2.7	P 31 1 2	P 64 2 2	2	2	0.00	2.9	0.001	0.11	0.11	0.10	---	1.4
**1t1f**	2.8	P 1 21 1	P 65	3	1	0.04	2.3	0.000	0.11	0.02	0.02	---	6.3
**1bqd**	2.1	P 1 21 1	C 2 2 21	2	1	0.02	5.8	0.003	0.11	0.11	0.11	---	---
**2f35**	1.9	C 2 2 21	P 41 21 2	2	1	0.02	11.6	0.016	0.11	0.10	0.10	0.09	2.0
**1zsv**	2.3	P 43	P 43 21 2	2	2	0.00	4.5	0.004	0.11	0.11	0.11	0.18	4.5
**1ju9**	2.0	P 1 21 1	C 2 2 21	2	1	0.12	6.4	0.006	0.11	0.11	0.11	0.14	---
**2qs3**	1.8	C 2 2 21	P 41 21 2	2	1	0.04	6.7	0.005	0.11	0.10	0.10	0.07	1.6
**1tt9**	3.4	P 4	P 4 21 2	2	2	0.00	8.1	0.008	0.11	0.07	0.02	0.76	---
**3cch**	2.6	P 1 21 1	C 2 2 21	2	6	0.00	2.6	0.001	0.11	0.11	0.11	0.27	2.9
**2fkz**	2.0	R 3 :H	R 3 2 :H	2	4	0.00	9.2	0.012	0.11	0.11	0.11	0.24	5.0
**3hbw**	1.9	P 1 21 1	C 2 2 21	2	1	0.02	7.1	0.007	0.11	0.11	0.11	---	1.6
**2cv9**	2.5	P 41	P 41 21 2	2	2	0.00	5.3	0.004	0.11	0.11	0.11	0.35	---
**3kqf**	1.8	P 1 21 1	C 2 2 21	2	3	0.05	9.8	0.010	0.11	0.10	0.10	0.06	---
**1z6t**	2.2	P 1	P 1 21 1	2	2	0.06	6.8	0.009	0.11	0.11	0.11	---	---

PDB Code	Resolution (Å)	Published Symmetry	Candidate Symmetry	No. of Cosets	Chains / asu	δ (°)	R_{symop} (I^{calc}) (%)	φ(g_i)	Δr_{sym} (Å)	Δr_{asu} (Å)	Δr_{chain} (Å)	Coord. Error (Å)	R_{symop} (I^{obs}) (%)
**2e0l**	1.6	I 41	I 41 2 2	2	1	0.00	7.3	0.008	0.11	0.11	0.11	0.06	1.1
**2z54**	2.4	P 61	P 61 2 2	2	1	0.00	5.7	0.006	0.11	0.11	0.11	0.22	2.7
**2hp4**	2.1	P 64	P 64 2 2	2	1	0.00	4.3	0.004	0.11	0.11	0.11	0.12	5.3
**2wer**	1.6	P 2 2 21	P 43 2 2	2	1	0.04	4.6	0.002	0.11	0.11	0.11	0.09	2.8
**2vat**	2.4	P 1 21 1	P 21 21 21	2	6	0.23	5.6	0.003	0.11	0.11	0.07	0.14	2.9
**3ehk**	3.2	P 41	P 41 2 2	2	3	0.00	3.4	0.002	0.11	0.11	0.09	0.43	---
**2pfd**	3.4	P 4	P 4 21 2	2	2	0.00	4.3	0.004	0.11	0.07	0.02	0.46	11.9
**2c9d**	2.8	P 1	C 1 2 1	2	5	0.12	10.8	0.018	0.11	0.10	0.08	0.41	12.4
**2p38**	1.8	C 1 2 1	F 2 2 2	2	1	0.01	7.3	0.006	0.11	0.11	0.11	0.09	1.7
**1q2x**	2.0	P 1 21 1	P 21 21 2	2	1	0.00	6.4	0.006	0.11	0.11	0.11	0.37	2.4
**2jat**	2.6	P 31	P 31 2 1	2	1	0.00	7.0	0.006	0.11	0.11	0.11	0.31	1.6
**1egw**	1.5	P 1	P 1 21 1	2	2	0.01	4.9	0.004	0.11	0.06	0.06	0.16	5.0
**3ggu**	1.8	P 61	P 61 2 2	2	1	0.00	9.0	0.014	0.11	0.11	0.11	0.12	1.0
**3f4w**	1.6	P 4	P 4 21 2	2	1	0.00	6.8	0.007	0.11	0.11	0.11	0.25	3.5
**1ylo**	2.2	I 41	F 41 3 2	6	1	0.07	5.3	0.004	0.11	0.11	0.11	0.12	4.4
**2qdi**	2.0	P 43	P 43 21 2	2	1	0.00	8.1	0.011	0.11	0.11	0.11	0.16	2.4
**1aib**	2.8	P 1 21 1	C 2 2 21	2	1	0.17	5.2	0.003	0.11	0.10	0.10	---	---
**3k44**	2.1	P 1 2 1	P 21 21 2	2	2	0.01	3.3	0.003	0.11	0.11	0.11	---	---
**1bay**	2.0	I 4	I 4 2 2	2	1	0.00	6.4	0.006	0.11	0.11	0.11	---	6.3
**2hrq**	2.7	P 1 21 1	P 21 21 21	2	3	0.01	5.2	0.004	0.11	0.11	0.11	0.38	---
**2fle**	1.9	P 61	P 61 2 2	2	1	0.00	6.9	0.009	0.11	0.11	0.11	---	1.7
**1zsr**	2.1	P 61	P 61 2 2	2	1	0.00	6.5	0.009	0.11	0.11	0.11	---	1.9
**2b6m**	2.7	P 32	P 62	2	1	0.00	3.9	0.002	0.11	0.11	0.11	---	2.1
**2c2b**	2.6	C 1 2 1	P 31 2 1	3	2	0.03	5.4	0.004	0.11	0.11	0.11	0.27	4.9
**2zgy**	1.9	P 41	P 41 2 2	2	1	0.00	3.5	0.003	0.11	0.11	0.11	0.20	1.6
**3kb4**	2.4	C 1 2 1	I 2 2 2	2	2	0.16	3.0	0.002	0.11	0.11	0.11	0.27	---
**3bcg**	2.5	P 31	P 64	2	1	0.00	3.2	0.001	0.11	0.09	0.09	---	---
**3ekp**	2.1	P 61	P 61 2 2	2	2	0.00	5.5	0.005	0.11	0.11	0.11	0.16	---
**3ds5**	2.4	P 1	I 41	4	1	0.06	5.2	0.003	0.12	0.11	0.11	0.19	2.3
**3fho**	2.8	C 1 2 1	C 2 2 21	2	1	0.04	6.7	0.004	0.12	0.11	0.11	0.35	---
**2isk**	2.1	P 1 21 1	P 21 21 21	2	4	0.01	8.4	0.009	0.12	0.12	0.12	---	7.7
**3e1y**	3.8	P 43	P 43 21 2	2	4	0.00	1.7	0.002	0.12	0.12	0.11	0.71	---
**4vhb**	1.8	P 1 21 1	C 2 2 21	2	1	0.15	8.2	0.009	0.12	0.11	0.11	---	---
**2z4b**	2.3	P 61	P 61 2 2	2	1	0.00	6.1	0.009	0.12	0.12	0.12	0.37	3.0
**3fwx**	2.0	C 2 2 21	P 41 21 2	2	1	0.14	8.4	0.009	0.12	0.11	0.11	0.12	5.5
**1gc0**	1.7	P 1	I 2 2 2	4	1	0.24	11.0	0.012	0.12	0.12	0.12	0.09	3.4
**1zlk**	3.1	C 1 2 1	I 21 21 21	2	1	0.12	8.5	0.010	0.12	0.07	0.07	0.39	5.1
**1nlx**	2.8	P 21 21 21	P 43 21 2	2	7	0.25	4.8	0.003	0.12	0.12	0.09	0.47	5.4
**1vc1**	2.0	P 1 21 1	C 2 2 21	2	1	0.00	7.9	0.010	0.12	0.12	0.12	0.20	1.7
**3gua**	3.1	P 41	P 41 21 2	2	5	0.00	4.3	0.002	0.12	0.12	0.12	0.39	---
**2zw2**	1.6	P 21 21 21	P 41 21 2	2	1	0.01	10.2	0.009	0.12	0.12	0.12	0.18	---
**3i4j**	1.7	P 1 21 1	C 2 2 21	2	2	0.02	6.4	0.004	0.12	0.12	0.12	0.21	2.2
**3bfv**	1.8	P 1	C 1 2 1	2	1	0.05	8.8	0.008	0.12	0.12	0.12	0.08	4.8
**2w3o**	1.9	P 3	P 3 1 2	2	2	0.00	5.5	0.004	0.12	0.12	0.12	0.10	2.6
**3f7f**	2.6	P 1	P 21 21 2	4	1	0.25	2.7	0.002	0.12	0.10	0.10	---	---

PDB Code	Resolution (Å)	Published Symmetry	Candidate Symmetry	No. of Cosets	Chains / asu	δ (°)	R_{symop} (I^{calc}) (%)	$\varphi(g_i)$	Δr_{sym} (Å)	Δr_{asu} (Å)	Δr_{chain} (Å)	Coord. Error (Å)	R_{symop} (I^{obs}) (%)
**3c37**	1.7	P 1	C 1 2 1	2	1	0.06	6.8	0.005	0.12	0.12	0.12	0.09	1.5
**2nt0**	1.8	P 1 21 1	C 2 2 21	2	2	0.28	6.7	0.005	0.12	0.12	0.12	0.08	5.0
**3g7v**	1.9	P 1 21 1	C 2 2 21	2	2	0.02	6.5	0.005	0.12	0.12	0.12	0.07	5.6
**1m4v**	1.9	P 63	P 63 2 2	2	1	0.00	8.1	0.009	0.12	0.12	0.12	0.19	3.7
**3eno**	3.0	P 65	P 65 2 2	2	3	0.00	4.0	0.006	0.12	0.12	0.11	---	4.5
**3cv6**	2.1	P 32	P 32 2 1	2	1	0.00	6.1	0.005	0.12	0.12	0.12	0.14	3.2
**1nzk**	1.9	C 1 2 1	C 2 2 21	2	2	0.02	5.8	0.005	0.12	0.12	0.12	---	2.2
**2bni**	1.5	P 31	P 31 2 1	2	2	0.00	10.0	0.026	0.12	0.12	0.12	---	2.4
**2rkf**	1.8	P 61	P 61 2 2	2	1	0.00	8.6	0.014	0.12	0.12	0.12	0.11	1.5
**1r8h**	1.9	P 61	P 61 2 2	2	3	0.00	5.6	0.006	0.12	0.12	0.12	0.12	1.3
**3ivt**	2.7	P 62	P 62 2 2	2	1	0.00	5.4	0.005	0.12	0.12	0.12	0.16	---
**2zdc**	2.0	P 1	R 3 :H	3	1	0.07	8.6	0.007	0.12	0.12	0.12	0.32	6.7
**2qfr**	2.4	I 41	I 41 2 2	2	1	0.00	5.8	0.005	0.12	0.12	0.12	---	5.3
**2w9t**	3.0	P 1 21 1	P 43	2	1	0.04	5.4	0.003	0.12	0.12	0.12	0.17	3.8
**2qak**	2.2	P 61	P 61 2 2	2	1	0.00	7.4	0.012	0.12	0.12	0.12	---	2.1
**2zwm**	2.0	P 31	P 31 2 1	2	1	0.00	3.7	0.003	0.12	0.12	0.12	0.12	1.8
**3gk0**	2.3	P 1	C 1 2 1	2	4	0.05	7.6	0.008	0.12	0.12	0.12	0.17	7.6
**1xpj**	2.3	P 1 21 1	P 21 21 2	2	2	0.20	8.5	0.011	0.12	0.12	0.12	0.19	2.4
**3gcf**	2.3	C 1 2 1	R 3 2 :H	3	5	0.00	5.9	0.007	0.12	0.12	0.12	---	---
**3fdw**	2.2	P 1 21 1	P 21 21 2	2	1	0.01	4.3	0.003	0.12	0.12	0.12	0.16	2.5
**3d4g**	2.3	P 1	P 21 21 2	4	2	0.18	3.9	0.004	0.13	0.12	0.12	0.20	6.4
**2g1p**	1.9	P 1 21 1	P 21 21 21	2	1	0.03	6.6	0.007	0.13	0.12	0.12	0.21	---
**1su1**	2.2	P 31	P 31 2 1	2	2	0.00	6.7	0.007	0.13	0.13	0.12	---	2.8
**3gz6**	2.9	P 32 1 2	P 65 2 2	2	1	0.00	3.9	0.003	0.13	0.13	0.13	0.36	2.5
**2gt2**	2.0	P 32	P 65	2	2	0.00	12.9	0.017	0.13	0.12	0.12	0.15	---
**2pkr**	2.4	P 1 21 1	P 21 21 2	2	6	0.04	7.9	0.006	0.13	0.13	0.12	0.40	4.1
**2vfx**	2.0	C 1 2 1	C 2 2 21	2	6	0.02	8.5	0.009	0.13	0.13	0.13	0.12	3.9
**3jze**	1.8	P 1 21 1	P 21 21 21	2	2	0.35	8.6	0.008	0.13	0.12	0.11	0.06	---
**2ra6**	1.5	P 1 21 1	P 21 21 2	2	2	0.02	9.0	0.012	0.13	0.13	0.13	---	2.3
**1t9k**	2.6	P 31 2 1	P 61 2 2	2	2	0.00	3.6	0.002	0.13	0.13	0.13	0.52	---
**1c9t**	3.3	P 1 21 1	C 2 2 21	2	6	0.29	7.4	0.005	0.13	0.11	0.08	---	14.9
**2o30**	1.7	P 1 21 1	P 21 21 21	2	1	0.08	10.4	0.014	0.13	0.12	0.12	0.09	2.9
**1t5r**	2.0	P 43	P 43 21 2	2	4	0.00	6.5	0.008	0.13	0.13	0.13	0.34	1.9
**3ddm**	2.6	P 21 21 21	P 43 21 2	2	2	0.02	3.8	0.002	0.13	0.13	0.13	0.59	3.6
**1jti**	2.3	P 1	C 1 2 1	2	1	0.16	5.4	0.006	0.13	0.13	0.13	0.35	---
**2gce**	1.9	C 1 2 1	C 2 2 21	2	2	0.07	5.5	0.006	0.13	0.13	0.13	0.13	3.0
**1ybu**	2.4	P 1 2 1	P 21 21 2	2	2	0.00	4.4	0.003	0.13	0.13	0.13	0.47	2.6
**1lzq**	2.2	P 61	P 61 2 2	2	1	0.00	4.1	0.005	0.13	0.12	0.12	0.34	2.3
**3iru**	2.3	P 3 2 1	P 63 2 2	2	1	0.00	7.0	0.007	0.13	0.13	0.13	0.16	---
**2o7m**	2.0	P 43	P 43 2 2	2	1	0.00	5.7	0.006	0.13	0.13	0.13	0.10	---
**2i0g**	2.5	P 61	P 61 2 2	2	1	0.00	5.2	0.008	0.13	0.13	0.13	0.43	1.9
**3bcx**	2.4	P 32	P 32 2 1	2	1	0.00	6.2	0.006	0.13	0.13	0.13	---	---
**1pt8**	2.2	P 62	P 62 2 2	2	1	0.00	5.0	0.004	0.13	0.13	0.13	0.10	4.7
**3ced**	2.1	I 4 2 2	I 4 3 2	3	1	0.20	6.4	0.004	0.13	0.06	0.06	0.08	---
**2efr**	1.8	P 1	P 1 21 1	2	2	0.09	10.2	0.018	0.13	0.13	0.13	0.29	3.8

PDB Code	Resolution (Å)	Published Symmetry	Candidate Symmetry	No. of Cosets	Chains / asu	δ (°)	R_{symop} (I^{calc}) (%)	φ(g_i)	Δr_{sym} (Å)	Δr_{asu} (Å)	Δr_{chain} (Å)	Coord. Error (Å)	R_{symop} (I^{obs}) (%)
**1npm**	2.1	P 1	C 1 2 1	2	1	0.09	4.1	0.005	0.13	0.02	0.02	---	---
**2pms**	2.9	P 32	P 65	2	2	0.00	4.8	0.003	0.13	0.13	0.13	0.27	4.7
**2fl0**	2.7	R 3 :H	R 3 2 :H	2	4	0.00	7.0	0.008	0.13	0.13	0.13	0.44	---
**3hvu**	2.0	P 1 21 1	P 21 21 2	2	2	0.03	6.3	0.006	0.13	0.13	0.13	0.12	4.5
**2awh**	2.0	P 1 21 1	P 21 21 2	2	1	0.04	8.8	0.012	0.13	0.13	0.13	0.13	5.3
**1vg8**	1.7	P 1	C 1 2 1	2	2	0.04	9.4	0.014	0.13	0.13	0.13	0.17	3.4
**1xpl**	2.0	P 1 21 1	P 21 21 2	2	2	0.03	5.5	0.004	0.13	0.13	0.13	---	4.7
**2hqm**	2.4	P 42	P 42 21 2	2	1	0.00	7.7	0.008	0.13	0.13	0.13	0.27	3.5
**2dyk**	2.0	P 1	C 1 2 1	2	1	0.07	10.4	0.012	0.13	0.13	0.13	0.19	4.7
**3g7g**	2.0	P 1 21 1	C 2 2 21	2	4	0.07	11.0	0.017	0.14	0.13	0.13	0.14	3.4
**2fbl**	1.9	P 32 2 1	P 65 2 2	2	1	0.00	10.8	0.011	0.14	0.13	0.13	0.11	1.9
**3b86**	2.0	P 43	P 43 21 2	2	1	0.00	8.0	0.014	0.14	0.13	0.13	---	2.7
**3g8k**	2.0	I 2 2 2	I 4 2 2	2	1	0.00	7.4	0.007	0.14	0.13	0.13	0.17	---
**2nnw**	2.7	P 31 1 2	P 61 2 2	2	2	0.00	2.7	0.001	0.14	0.13	0.13	---	4.1
**2pjl**	2.3	R 3 :H	R 3 2 :H	2	1	0.00	7.2	0.008	0.14	0.14	0.14	0.20	1.7
**2g9z**	2.0	P 1	C 1 2 1	2	1	0.06	7.6	0.007	0.14	0.14	0.14	---	3.0
**3cf2**	3.5	P 3	P 6 2 2	4	1	0.00	2.1	0.001	0.14	0.00	0.00	1.15	---
**2r8e**	1.4	P 1	I 4	4	2	0.11	6.9	0.007	0.14	0.14	0.13	0.03	3.5
**2gu9**	1.4	P 1	C 1 2 1	2	1	0.09	12.2	0.029	0.14	0.13	0.13	---	---
**3esi**	2.5	P 41	P 41 21 2	2	2	0.00	3.0	0.002	0.14	0.14	0.14	0.36	---
**1rbq**	2.1	P 31	P 31 2 1	2	2	0.00	5.1	0.005	0.14	0.13	0.11	0.11	10.3
**1xs2**	2.3	P 4	P 4 21 2	2	2	0.00	6.9	0.007	0.14	0.14	0.14	0.16	---
**2w9s**	1.8	P 1 2 1	P 62	3	2	0.00	5.4	0.003	0.14	0.13	0.13	0.08	5.5
**2nnn**	2.4	P 31	P 31 2 1	2	5	0.00	6.4	0.007	0.14	0.14	0.14	0.20	---
**1gzm**	2.6	P 31	P 64	2	1	0.00	3.8	0.002	0.14	0.14	0.14	0.46	4.1
**1uzi**	1.9	I 4	I 4 2 2	2	1	0.00	6.2	0.007	0.14	0.14	0.14	0.10	2.1
**3eej**	2.1	P 41	P 41 21 2	2	1	0.00	7.6	0.012	0.14	0.14	0.14	0.14	1.8
**1j48**	1.8	P 31	P 31 2 1	2	1	0.00	7.9	0.009	0.14	0.14	0.14	---	---
**3bx8**	2.0	P 1	C 1 2 1	2	4	0.03	7.5	0.006	0.14	0.14	0.14	---	3.6
**2ntt**	1.6	P 1 21 1	P 21 21 21	2	1	0.02	7.1	0.006	0.14	0.14	0.14	0.05	3.5
**1pro**	1.8	P 61	P 61 2 2	2	1	0.00	8.7	0.019	0.14	0.14	0.14	---	---
**2fb5**	2.0	C 1 2 1	R 3 2 :H	3	1	0.04	7.4	0.008	0.14	0.14	0.14	0.09	3.1
**2nsx**	2.1	P 1 21 1	C 2 2 21	2	2	0.03	6.3	0.006	0.14	0.14	0.14	0.13	4.6
**3cpq**	1.9	P 43	P 43 21 2	2	1	0.00	7.2	0.011	0.14	0.14	0.14	0.13	1.4
**1mzn**	1.9	P 1	C 1 2 1	2	4	0.02	7.2	0.007	0.14	0.14	0.13	---	---
**1zg5**	2.3	P 1 21 1	P 21 21 2	2	2	0.00	5.7	0.007	0.14	0.14	0.14	0.40	2.6
**2f8f**	2.1	P 1 21 1	C 2 2 21	2	1	0.09	11.4	0.014	0.14	0.14	0.14	0.17	3.2
**2w1k**	2.1	P 2 3	P 4 3 2	2	1	0.00	5.8	0.004	0.14	0.14	0.14	0.14	5.1
**2q6o**	2.0	P 3	P 3 2 1	2	1	0.00	7.2	0.006	0.15	0.14	0.14	0.17	---
**2cg4**	2.4	P 4	P 4 2 2	2	1	0.00	5.0	0.006	0.15	0.14	0.14	0.20	4.7
**2qtu**	2.5	P 61	P 61 2 2	2	1	0.00	5.7	0.011	0.15	0.14	0.14	0.56	1.9
**2hjd**	2.1	P 43	P 43 21 2	2	2	0.00	6.9	0.008	0.15	0.14	0.14	0.23	0.7
**2qgq**	2.0	P 1	C 2 2 21	4	2	0.04	9.0	0.011	0.15	0.14	0.14	0.18	---
**2fpz**	2.0	P 31	P 31 2 1	2	2	0.00	8.3	0.010	0.15	0.15	0.14	0.24	4.0
**2aqu**	2.0	P 61	P 61 2 2	2	1	0.00	9.6	0.016	0.15	0.15	0.15	0.28	3.0

PDB Code	Resolution (Å)	Published Symmetry	Candidate Symmetry	No. of Cosets	Chains / asu	δ (°)	R_{symop} (I^{calc}) (%)	φ(g_i)	Δr_{sym} (Å)	Δr_{asu} (Å)	Δr_{chain} (Å)	Coord. Error (Å)	R_{symop} (I^{obs}) (%)
**2z6y**	2.0	P 1 21 1	C 2 2 21	2	2	0.05	7.7	0.006	0.15	0.15	0.15	0.22	---
**3fht**	2.2	P 1 21 1	P 21 21 21	2	1	0.02	11.4	0.017	0.15	0.15	0.15	0.14	5.2
**1v9e**	1.9	P 61	P 61 2 2	2	1	0.00	8.9	0.014	0.15	0.15	0.15	0.34	2.5
**2ntd**	2.5	P 1 21 1	C 2 2 21	2	2	0.31	7.0	0.007	0.15	0.15	0.15	0.36	4.2
**3ebk**	1.9	P 41	P 41 21 2	2	1	0.00	4.8	0.006	0.15	0.15	0.15	---	---
**3gxi**	1.8	P 1 21 1	C 2 2 21	2	2	0.05	6.6	0.006	0.15	0.15	0.15	0.10	3.3
**1u4j**	2.2	R 3 :H	R 3 2 :H	2	1	0.00	5.5	0.004	0.15	0.15	0.15	0.19	---
**1bwb**	1.8	P 61	P 61 2 2	2	1	0.00	10.6	0.027	0.15	0.15	0.15	---	---
**1wyy**	2.2	R 3 :H	R 3 2 :H	2	1	0.00	5.2	0.002	0.15	0.15	0.15	0.27	4.2
**1sed**	2.1	P 1	R 3 :H	3	1	0.44	6.8	0.004	0.15	0.13	0.13	0.19	5.8
**3eek**	2.0	P 41	P 41 21 2	2	1	0.00	8.0	0.013	0.15	0.15	0.15	0.15	2.8
**2az5**	2.1	R 3 :H	R 3 2 :H	2	2	0.00	9.9	0.010	0.15	0.15	0.15	0.15	4.7
**3dto**	3.3	P 1 21 1	P 21 21 21	2	2	0.01	2.9	0.001	0.15	0.15	0.15	0.87	---
**2r4b**	2.4	P 43	P 43 21 2	2	1	0.00	6.6	0.010	0.15	0.15	0.15	0.25	3.5
**1y9k**	2.4	P 32	P 32 2 1	2	2	0.00	9.8	0.018	0.15	0.15	0.15	0.16	1.4
**1hm0**	2.3	R 3 :H	R 3 2 :H	2	1	0.00	3.8	0.002	0.15	0.15	0.15	---	4.7
**1xss**	1.6	C 1 2 1	I 2 2 2	2	1	0.09	6.4	0.004	0.15	0.15	0.15	---	---
**3cue**	3.7	P 1 21 1	P 41 21 2	4	6	0.28	2.3	0.002	0.15	0.10	0.06	---	6.1
**1ylq**	2.0	P 32 2 1	P 65 2 2	2	1	0.00	10.4	0.012	0.15	0.15	0.15	0.13	1.3
**2jhv**	2.1	C 1 2 1	R 3 2 :H	3	2	0.23	6.4	0.005	0.15	0.14	0.14	0.15	11.3
**1wmq**	1.6	R 3 :H	R 3 2 :H	2	1	0.00	10.3	0.013	0.15	0.15	0.15	0.22	1.1
**1mw5**	2.1	P 64	P 64 2 2	2	1	0.00	6.2	0.009	0.16	0.15	0.15	---	1.2
**2qe2**	2.9	P 21 21 21	P 41 21 2	2	1	0.03	6.3	0.004	0.16	0.15	0.15	0.40	1.5
**2idr**	1.9	P 61	P 61 2 2	2	1	0.00	7.4	0.011	0.16	0.15	0.15	---	1.5
**1g2x**	2.5	C 1 2 1	R 3 2 :H	3	1	0.02	7.6	0.008	0.16	0.15	0.15	0.36	4.0
**2qs1**	1.8	C 2 2 21	P 41 21 2	2	1	0.00	7.5	0.006	0.16	0.16	0.16	0.08	2.1
**2ggj**	2.5	P 4	P 4 21 2	2	2	0.00	7.3	0.007	0.16	0.16	0.16	0.15	4.8
**2znz**	2.4	P 1 2 1	C 2 2 2	2	4	0.12	8.3	0.008	0.16	0.15	0.15	---	4.9
**3ec3**	1.9	P 31	P 31 2 1	2	1	0.00	8.6	0.012	0.16	0.15	0.15	0.10	1.0
**1yak**	2.5	C 2 2 21	P 41 21 2	2	2	0.02	5.3	0.004	0.16	0.15	0.15	0.42	2.6
**2nt1**	2.3	P 1 21 1	C 2 2 21	2	2	0.19	6.8	0.005	0.16	0.16	0.16	0.16	5.1
**1jxo**	2.3	P 1	P 1 21 1	2	1	0.04	9.5	0.011	0.16	0.16	0.16	0.28	2.7
**2ise**	2.2	P 1 21 1	P 21 21 2	2	1	0.00	7.4	0.009	0.16	0.16	0.16	0.37	---
**1i0c**	2.0	P 31	P 31 2 1	2	1	0.00	10.4	0.020	0.16	0.15	0.15	0.21	4.4
**2v8v**	2.9	P 1	C 1 2 1	2	2	0.51	6.3	0.005	0.16	0.16	0.16	0.32	8.7
**1zg1**	2.3	P 1 21 1	P 21 21 2	2	2	0.07	5.5	0.006	0.16	0.16	0.15	0.44	1.8
**1sgu**	1.9	P 61	P 61 2 2	2	1	0.00	10.9	0.022	0.16	0.16	0.16	0.20	2.5
**1jdv**	2.0	P 1 21 1	C 2 2 21	2	3	0.00	9.3	0.010	0.16	0.16	0.15	---	---
**2nud**	2.3	P 1 21 1	P 21 21 2	2	2	0.08	9.3	0.011	0.16	0.16	0.16	0.31	4.9
**2rab**	2.5	P 41	P 41 21 2	2	1	0.00	7.3	0.009	0.16	0.16	0.16	0.18	3.0
**2a1f**	2.1	P 1	C 1 2 1	2	3	0.14	9.6	0.010	0.16	0.16	0.16	0.14	8.4
**3gr0**	2.3	P 31	P 31 2 1	2	2	0.00	12.1	0.027	0.16	0.16	0.16	0.17	1.6
**3cyk**	1.4	P 1 21 1	P 21 21 2	2	2	0.02	9.0	0.010	0.16	0.16	0.16	0.04	1.9
**1hdu**	1.8	P 1	P 1 21 1	2	2	0.00	8.1	0.008	0.16	0.11	0.07	---	---
**1hee**	1.8	P 1	P 1 21 1	2	2	0.00	8.1	0.008	0.16	0.11	0.07	---	---

PDB Code	Resolution (Å)	Published Symmetry	Candidate Symmetry	No. of Cosets	Chains / asu	δ (°)	R_{symop} (I^{calc}) (%)	φ(g_i)	Δr_{sym} (Å)	Δr_{asu} (Å)	Δr_{chain} (Å)	Coord. Error (Å)	R_{symop} (I^{obs}) (%)
**1lyq**	1.5	P 41	P 41 21 2	2	1	0.00	10.3	0.014	0.16	0.16	0.16	0.10	3.9
**3bb8**	2.4	P 32	P 32 2 1	2	1	0.00	5.9	0.006	0.16	0.16	0.16	---	---
**1r0d**	1.9	C 1 2 1	F 2 2 2	2	4	0.04	8.9	0.010	0.16	0.16	0.16	0.33	---
**3dby**	2.1	R 3 :H	R 3 2 :H	2	10	0.00	6.5	0.006	0.16	0.16	0.16	0.14	---
**1ddn**	3.0	P 41	P 41 21 2	2	2	0.00	5.6	0.005	0.16	0.16	0.16	---	---
**3gmw**	2.1	P 1	C 1 2 1	2	2	0.18	7.0	0.006	0.16	0.16	0.16	0.12	4.9
**1sql**	2.2	P 1	P 1 21 1	2	8	0.15	10.3	0.014	0.16	0.16	0.16	---	5.1
**2c10**	2.5	P 43	P 43 21 2	2	2	0.00	5.2	0.004	0.16	0.16	0.16	0.17	7.4
**2b7z**	2.2	P 61	P 61 2 2	2	1	0.00	10.9	0.024	0.16	0.16	0.16	---	3.1
**1g0z**	2.2	R 3 :H	R 3 2 :H	2	1	0.00	7.0	0.006	0.16	0.16	0.16	0.23	---
**1dc3**	2.5	I 41	I 41 2 2	2	1	0.00	6.4	0.008	0.17	0.17	0.17	---	1.1
**3hhl**	2.6	R 3 :H	I 21 3	4	1	0.11	6.7	0.006	0.17	0.16	0.16	0.12	3.2
**2hen**	2.6	P 1 21 1	P 21 21 21	2	2	0.18	7.0	0.007	0.17	0.16	0.16	0.41	3.9
**1zkd**	2.1	P 61	P 61 2 2	2	1	0.00	7.0	0.008	0.17	0.17	0.17	0.18	---
**2c80**	2.3	P 32	P 32 2 1	2	1	0.00	8.6	0.011	0.17	0.17	0.17	0.18	3.2
**2ojt**	2.0	C 2 2 21	P 41 21 2	2	1	0.08	7.4	0.006	0.17	0.15	0.15	0.10	3.0
**3dgd**	1.4	P 1 21 1	P 21 21 2	2	2	0.02	9.2	0.011	0.17	0.17	0.17	0.04	1.9
**2djw**	2.4	P 32	P 32 2 1	2	5	0.00	6.1	0.006	0.17	0.17	0.16	0.23	1.3
**2bgj**	2.1	P 1 21 1	P 21 21 2	2	2	0.08	6.6	0.008	0.17	0.17	0.17	---	5.3
**1zpk**	1.7	P 61	P 61 2 2	2	1	0.00	8.5	0.013	0.17	0.16	0.16	---	1.0
**3cap**	2.9	R 3 :H	R 3 2 :H	2	1	0.00	4.6	0.006	0.17	0.17	0.17	---	3.0
**3eel**	1.9	P 41	P 41 21 2	2	1	0.00	8.8	0.015	0.17	0.17	0.17	0.12	3.1
**2c1j**	2.6	P 1 21 1	C 2 2 21	2	2	0.03	10.5	0.014	0.17	0.08	0.08	0.30	4.1
**3hum**	2.3	P 21 21 21	P 41 21 2	2	1	0.06	6.1	0.004	0.17	0.17	0.17	0.17	---
**1j51**	2.2	P 1	P 1 21 1	2	2	0.09	9.2	0.013	0.17	0.17	0.17	---	---
**1zlf**	2.3	P 61	P 61 2 2	2	1	0.00	8.2	0.015	0.17	0.17	0.17	---	1.7
**1wrq**	2.2	R 3 :H	R 3 2 :H	2	1	0.00	10.4	0.014	0.17	0.17	0.17	0.22	2.6
**1w5e**	3.0	P 1	P 31	3	3	0.36	4.8	0.003	0.17	0.17	0.16	---	7.4
**3h8b**	1.8	C 1 2 1	C 2 2 21	2	3	0.05	7.1	0.007	0.17	0.10	0.09	---	---
**2b60**	2.2	P 61	P 61 2 2	2	1	0.00	11.1	0.022	0.17	0.17	0.17	---	4.8
**3f55**	2.8	P 41	P 41 21 2	2	2	0.00	6.6	0.008	0.17	0.17	0.17	---	---
**1sof**	2.6	R 3 :H	R 3 2 :H	2	4	0.00	8.5	0.011	0.17	0.17	0.17	0.43	4.5
**2i5c**	1.8	C 1 2 1	P 31 2 1	3	1	0.03	9.0	0.011	0.17	0.16	0.16	0.05	1.6
**3bju**	2.3	P 32	P 32 2 1	2	2	0.00	10.0	0.013	0.17	0.17	0.17	0.17	6.9
**1rit**	2.9	P 32	P 32 2 1	2	2	0.00	4.5	0.003	0.17	0.17	0.17	---	10.8
**1tjc**	2.3	P 32	P 32 2 1	2	1	0.00	6.5	0.008	0.17	0.17	0.17	0.15	1.9
**3h0r**	3.0	P 1	P 1 21 1	2	12	0.12	5.1	0.004	0.17	0.17	0.16	0.55	6.2
**2ggi**	2.2	P 4	P 4 21 2	2	2	0.00	7.2	0.007	0.17	0.17	0.17	0.11	3.5
**2q64**	2.5	P 61	P 61 2 2	2	1	0.00	6.7	0.011	0.17	0.17	0.17	0.26	1.8
**2ijz**	3.0	P 1	F 2 3	12	1	0.20	7.3	0.005	0.17	0.17	0.17	0.35	---
**3htm**	2.5	P 1	C 2 2 2	4	1	0.71	7.6	0.010	0.17	0.17	0.17	---	---
**2j69**	3.0	C 1 2 1	I 4 2 2	4	1	0.31	4.6	0.003	0.17	0.14	0.14	0.34	7.3
**1tyh**	2.5	P 41	P 41 21 2	2	2	0.00	5.2	0.007	0.18	0.18	0.17	---	---
**3h07**	2.0	P 1 21 1	P 21 21 2	2	1	0.13	12.9	0.023	0.18	0.14	0.14	0.15	4.7
**2p1y**	2.4	P 1 21 1	C 2 2 21	2	2	0.04	7.6	0.008	0.18	0.18	0.17	0.18	1.8

PDB Code	Resolution (Å)	Published Symmetry	Candidate Symmetry	No. of Cosets	Chains / asu	δ (°)	R_{symop} (I^{calc}) (%)	$\varphi(g_i)$	Δr_{sym} (Å)	Δr_{asu} (Å)	Δr_{chain} (Å)	Coord. Error (Å)	R_{symop} (I^{obs}) (%)
**1r0c**	2.4	R 3 :H	R 3 2 :H	2	2	0.00	5.2	0.003	0.18	0.18	0.18	0.42	4.4
**1ql3**	1.4	P 21 21 21	P 43 21 2	2	2	0.67	13.1	0.020	0.18	0.16	0.14	0.15	---
**1qx4**	1.8	P 1 21 1	C 2 2 21	2	1	0.01	9.2	0.008	0.18	0.18	0.18	---	---
**3ch1**	2.3	P 1 2 1	P 21 21 2	2	6	0.08	4.5	0.004	0.18	0.18	0.18	0.17	2.9
**3ffq**	2.4	I 4	I 4 2 2	2	1	0.00	10.8	0.019	0.18	0.18	0.18	0.24	1.7
**1ahe**	2.3	P 1 21 1	C 2 2 21	2	1	0.03	8.0	0.011	0.18	0.18	0.18	---	---
**3eo1**	3.1	P 21 21 21	P 43 21 2	2	6	0.24	3.4	0.001	0.18	0.18	0.05	---	9.9
**2efs**	2.0	P 1	P 1 21 1	2	2	0.33	9.0	0.017	0.18	0.18	0.18	0.21	2.7
**2rmc**	1.6	P 1 21 1	P 21 21 2	2	2	0.00	7.5	0.010	0.18	0.18	0.18	---	---
**1ynb**	1.8	C 1 2 1	R 3 2 :H	3	1	0.03	8.5	0.011	0.18	0.18	0.18	0.25	1.6
**1anx**	1.9	P 1	R 3 :H	3	1	0.12	7.6	0.009	0.18	0.18	0.18	---	---
**2gax**	1.8	C 2 2 21	P 43 21 2	2	1	0.03	9.6	0.011	0.18	0.18	0.18	0.12	4.2
**2gdd**	2.4	P 31	P 31 2 1	2	2	0.00	7.6	0.009	0.18	0.18	0.18	0.40	3.9
**2r9z**	2.1	P 41	P 41 21 2	2	1	0.00	8.5	0.012	0.18	0.18	0.18	0.12	2.8
**2c7n**	2.1	P 1	C 1 2 1	2	6	0.44	14.7	0.029	0.18	0.18	0.16	0.12	5.1
**1b0w**	1.8	C 2 2 21	P 61 2 2	3	1	0.00	10.5	0.016	0.18	0.18	0.18	---	5.5
**1r1u**	2.0	P 31	P 31 2 1	2	2	0.00	7.4	0.010	0.18	0.18	0.18	0.14	---
**2oov**	1.7	P 1 21 1	C 2 2 21	2	3	0.06	4.5	0.002	0.18	0.18	0.18	0.06	---
**1jch**	3.0	P 1 21 1	C 2 2 21	2	2	0.05	3.0	0.001	0.18	0.02	0.02	0.56	5.5
**3exg**	3.0	P 1	P 1 21 1	2	16	0.05	5.3	0.004	0.18	0.18	0.18	0.35	7.1
**3b4m**	2.8	P 31	P 31 2 1	2	2	0.00	8.4	0.013	0.18	0.18	0.18	0.38	---
**2qhc**	2.8	P 61	P 61 2 2	2	1	0.00	6.5	0.009	0.18	0.17	0.17	0.32	3.9
**2qko**	2.4	P 31	P 61 2 2	4	1	0.00	7.3	0.008	0.19	0.18	0.18	0.23	---
**3dfv**	3.1	C 1 2 1	I 2 2 2	2	1	0.01	13.9	0.022	0.19	0.14	0.14	---	2.7
**2gci**	1.6	C 1 2 1	C 2 2 21	2	2	0.07	7.4	0.014	0.19	0.15	0.15	0.08	4.4
**3eem**	2.1	P 41	P 41 21 2	2	1	0.00	8.4	0.016	0.19	0.18	0.18	0.14	2.9
**1yup**	2.1	P 1	P 1 21 1	2	4	0.00	12.2	0.023	0.19	0.19	0.18	0.24	3.4
**3h89**	2.5	C 1 2 1	C 2 2 21	2	3	0.09	12.1	0.018	0.19	0.18	0.16	---	---
**3gxf**	2.4	P 1 21 1	C 2 2 21	2	2	0.01	7.3	0.006	0.19	0.19	0.19	0.18	7.2
**1ahx**	2.0	P 1 21 1	C 2 2 21	2	1	0.10	9.3	0.013	0.19	0.19	0.19	---	---
**1t6g**	1.8	P 32	P 32 2 1	2	2	0.00	5.2	0.003	0.19	0.19	0.19	0.07	1.4
**2vi5**	2.3	P 1	C 1 2 1	2	5	0.31	10.1	0.010	0.19	0.17	0.16	0.27	12.9
**3c3e**	3.0	P 32	P 65	2	2	0.00	4.0	0.003	0.19	0.18	0.18	0.63	9.1
**1ixy**	2.5	P 1 21 1	P 21 21 21	2	1	0.30	9.5	0.012	0.19	0.18	0.18	---	8.7
**2fs9**	2.3	P 31	P 31 2 1	2	2	0.00	8.2	0.009	0.19	0.19	0.19	0.41	4.0
**2qcq**	2.2	R 3 :H	R 3 2 :H	2	1	0.00	7.6	0.012	0.19	0.19	0.19	0.19	2.9
**3gxm**	2.2	P 1 21 1	C 2 2 21	2	2	0.03	8.5	0.009	0.19	0.19	0.19	0.20	8.2
**2f34**	1.7	C 2 2 21	P 41 21 2	2	1	0.15	12.4	0.019	0.19	0.16	0.16	0.07	3.9
**1keo**	2.2	I 4	I 4 2 2	2	1	0.00	7.4	0.009	0.19	0.19	0.19	0.58	4.4
**2fs8**	2.5	P 31	P 31 2 1	2	2	0.00	7.7	0.009	0.19	0.19	0.19	0.49	3.8
**1vg9**	2.5	P 1 21 1	P 21 21 2	2	4	0.13	6.3	0.005	0.19	0.19	0.19	0.36	---
**1xdl**	3.0	P 21 21 2	P 42 21 2	2	4	0.01	7.7	0.007	0.19	0.19	0.19	0.94	3.9
**1arg**	2.2	P 1 21 1	C 2 2 21	2	1	0.03	9.3	0.013	0.19	0.19	0.19	---	---
**3e4e**	2.6	P 43	P 43 21 2	2	1	0.00	6.6	0.010	0.19	0.19	0.19	0.28	2.6
**1hxb**	2.3	P 61	P 61 2 2	2	1	0.00	9.1	0.021	0.19	0.19	0.19	---	2.0

PDB Code	Resolution (Å)	Published Symmetry	Candidate Symmetry	No. of Cosets	Chains / asu	δ (°)	R_{symop} (I^{calc}) (%)	φ(g_i)	Δr_{sym} (Å)	Δr_{asu} (Å)	Δr_{chain} (Å)	Coord. Error (Å)	R_{symop} (I^{obs}) (%)
**2z02**	2.0	P 21 21 21	P 41 21 2	2	1	0.00	6.0	0.003	0.19	0.19	0.19	0.23	1.6
**2zok**	2.1	P 1 21 1	P 21 21 2	2	6	0.01	8.1	0.008	0.19	0.19	0.19	0.12	3.1
**2zg6**	2.4	P 32	P 32 2 1	2	1	0.00	6.1	0.008	0.19	0.19	0.19	0.14	---
**2jev**	2.3	P 43	P 43 2 2	2	1	0.00	6.3	0.010	0.20	0.19	0.19	0.21	1.6
**2oqe**	1.6	P 1 21 1	C 2 2 21	2	3	0.14	5.4	0.003	0.20	0.20	0.19	0.05	---
**1ahy**	2.3	P 1 21 1	C 2 2 21	2	1	0.09	8.8	0.011	0.20	0.20	0.20	---	---
**3k8e**	2.5	C 1 2 1	C 2 2 21	2	2	0.03	10.5	0.013	0.20	0.20	0.20	0.19	---
**1ahf**	2.3	P 1 21 1	C 2 2 21	2	1	0.06	8.4	0.011	0.20	0.20	0.20	---	---
**2c1c**	2.3	P 1 21 1	P 21 21 2	2	1	0.13	11.1	0.015	0.20	0.20	0.20	0.60	---
**2g45**	2.0	P 64	P 64 2 2	2	2	0.00	3.7	0.003	0.20	0.20	0.20	0.31	---
**2qfj**	2.1	P 31	P 31 2 1	2	1	0.00	16.2	0.049	0.20	0.17	0.17	0.41	---
**1vgz**	3.0	P 31	P 31 1 2	2	1	0.00	9.9	0.017	0.20	0.20	0.20	---	1.1
**3h2v**	2.9	P 1	P 1 21 1	2	4	0.23	7.6	0.008	0.20	0.20	0.19	0.40	8.5
**1xdm**	3.0	P 21 21 2	P 42 21 2	2	4	0.02	6.3	0.004	0.20	0.20	0.20	1.15	4.4
**3dhr**	2.0	P 1	C 1 2 1	2	4	0.11	12.7	0.020	0.20	0.20	0.20	0.15	10.0
**2fxr**	2.5	P 31	P 31 2 1	2	2	0.00	8.2	0.011	0.20	0.20	0.20	---	3.8
**1a94**	2.0	P 1 1 21	P 21 21 21	2	2	0.00	12.5	0.030	0.20	0.19	0.19	---	---
**1dmy**	2.5	P 1	C 1 2 1	2	1	0.09	8.8	0.015	0.20	0.20	0.20	---	---
**2fww**	2.2	P 31	P 31 2 1	2	2	0.00	8.8	0.012	0.20	0.20	0.20	---	3.5
**3e54**	2.5	P 61	P 61 2 2	2	1	0.00	4.3	0.004	0.20	0.16	0.16	0.42	---
**3hl2**	2.8	P 31 1 2	P 61 2 2	2	2	0.00	5.2	0.004	0.20	0.20	0.20	0.40	---
**1oaz**	2.8	P 21 21 21	P 41 21 2	2	3	0.08	4.7	0.002	0.20	0.17	0.07	0.26	7.3
**2gbo**	2.2	P 62	P 62 2 2	2	1	0.00	4.0	0.007	0.20	0.20	0.20	0.12	---
**3bq4**	2.7	I 4	I 4 2 2	2	3	0.00	4.6	0.006	0.21	0.20	0.20	0.85	3.6
**1yfo**	2.3	P 1 21 1	C 2 2 21	2	1	0.09	21.7	0.112	0.21	0.16	0.16	---	21.6
**2gmq**	1.8	I 21 21 21	I 41 2 2	2	1	0.19	10.5	0.012	0.21	0.20	0.20	0.08	---
**3ng1**	2.3	P 21 21 21	P 43 21 2	2	1	0.47	8.3	0.007	0.21	0.17	0.17	---	12.4
**1je8**	2.1	P 1 21 1	P 21 21 2	2	2	0.00	6.9	0.008	0.21	0.20	0.20	0.27	2.5
**1yax**	2.4	C 1 2 1	I 2 2 2	2	2	0.06	8.3	0.010	0.21	0.21	0.20	0.23	2.0
**2gqd**	2.3	P 65	P 65 2 2	2	1	0.00	7.9	0.014	0.21	0.21	0.21	0.24	2.8
**2j2p**	2.8	P 32	P 32 2 1	2	3	0.00	8.5	0.010	0.21	0.21	0.20	0.30	8.5
**3gh8**	2.6	P 1 21 1	P 21 21 21	2	4	0.08	7.7	0.010	0.21	0.21	0.21	0.27	---
**3dex**	2.7	P 1 21 1	C 2 2 21	2	4	0.05	8.6	0.009	0.21	0.21	0.20	0.52	---
**3ebn**	2.4	P 1	I 2 2 2	4	1	0.08	10.3	0.020	0.21	0.21	0.21	---	3.5
**1mes**	1.9	P 61	P 61 2 2	2	1	0.00	12.1	0.034	0.21	0.21	0.21	---	---
**1vqv**	2.6	P 43	P 43 21 2	2	1	0.00	6.3	0.011	0.21	0.21	0.21	---	0.9
**3dh2**	2.3	C 1 2 1	I 2 2 2	2	2	0.04	12.7	0.019	0.21	0.21	0.20	0.21	4.1
**1bd3**	1.9	P 1 21 1	C 2 2 21	2	2	0.01	8.1	0.013	0.21	0.21	0.21	---	---
**1h9m**	1.6	R 3 :H	R 3 2 :H	2	1	0.00	8.8	0.010	0.21	0.21	0.21	---	1.0
**1axa**	2.0	P 61	P 61 2 2	2	1	0.00	7.9	0.025	0.21	0.21	0.21	---	---
**1xsz**	1.4	P 31	P 31 2 1	2	1	0.00	6.5	0.004	0.21	0.21	0.21	0.14	---
**1f28**	1.9	P 1 21 1	P 21 21 2	2	2	0.24	6.4	0.005	0.21	0.08	0.08	0.21	---
**1a64**	2.0	P 43	P 43 21 2	2	1	0.00	10.8	0.026	0.22	0.21	0.21	---	---
**1xpy**	2.3	P 4	P 4 21 2	2	2	0.00	7.8	0.008	0.22	0.21	0.21	0.14	---
**2r8b**	2.6	P 21 21 21	P 41 21 2	2	1	0.17	7.2	0.008	0.22	0.21	0.21	0.25	3.5

PDB Code	Resolution (Å)	Published Symmetry	Candidate Symmetry	No. of Cosets	Chains / asu	δ (°)	R_{symop} (I^{calc}) (%)	$\varphi(g_i)$	Δr_{sym} (Å)	Δr_{asu} (Å)	Δr_{chain} (Å)	Coord. Error (Å)	R_{symop} (I^{obs}) (%)
**1qzu**	2.9	R 3 :H	R 3 2 :H	2	2	0.00	4.9	0.005	0.22	0.21	0.21	0.77	2.9
**2zbu**	2.1	R 3 :H	R 3 2 :H	2	2	0.00	7.5	0.007	0.22	0.22	0.22	0.24	2.6
**2r4e**	2.1	I 2 2 2	I 4 2 2	2	1	0.01	7.1	0.005	0.22	0.22	0.22	0.14	3.6
**2ofj**	2.3	P 1 21 1	P 21 21 2	2	2	0.08	12.5	0.022	0.22	0.22	0.21	0.25	5.4
**2b5u**	2.3	P 1 21 1	C 2 2 21	2	2	0.02	6.2	0.006	0.22	0.22	0.22	---	2.8
**3gqx**	2.5	P 41	P 41 21 2	2	1	0.00	7.6	0.014	0.22	0.01	0.01	---	1.6
**3gxd**	2.5	P 1 21 1	C 2 2 21	2	2	0.01	6.7	0.007	0.22	0.22	0.22	0.23	4.6
**2zn9**	2.4	P 41	P 41 2 2	2	1	0.00	7.4	0.012	0.22	0.22	0.22	0.25	1.0
**1a6p**	2.1	P 43	P 43 21 2	2	1	0.00	8.7	0.018	0.22	0.21	0.21	---	2.6
**1t3u**	2.5	P 41	P 41 21 2	2	2	0.00	10.4	0.020	0.22	0.22	0.22	0.41	---
**1ry9**	1.8	P 1 21 1	P 21 21 21	2	2	0.77	10.5	0.012	0.22	0.21	0.20	0.09	9.2
**2v65**	2.4	C 2 2 2	P 42 21 2	2	1	0.00	12.3	0.020	0.22	0.22	0.22	0.28	11.7
**1upu**	2.5	P 1 21 1	C 2 2 21	2	2	0.03	8.5	0.014	0.22	0.22	0.22	---	---
**3b85**	2.4	P 3	P 6	2	1	0.00	7.9	0.009	0.22	0.22	0.22	0.18	---
**3ily**	2.2	P 43	P 43 21 2	2	1	0.00	8.9	0.023	0.22	0.22	0.22	0.20	1.9
**2opz**	3.0	I 21 3	I 41 3 2	2	2	0.00	4.0	0.003	0.22	0.22	0.21	---	---
**2pa2**	2.5	P 31 2 1	P 61 2 2	2	1	0.00	7.2	0.008	0.22	0.17	0.17	0.52	---
**3co5**	2.4	P 32	P 32 2 1	2	1	0.00	9.6	0.016	0.22	0.22	0.22	0.20	0.8
**1rpi**	1.9	P 41	P 41 21 2	2	1	0.00	9.1	0.017	0.22	0.22	0.22	---	---
**1tku**	1.7	P 1	C 1 2 1	2	1	0.11	8.6	0.007	0.22	0.22	0.22	---	2.3
**1uvh**	2.8	R 3 2 :H	F 4 3 2	4	1	0.03	5.7	0.005	0.22	0.22	0.22	---	2.9
**1xiq**	3.1	P 1 21 1	C 2 2 21	2	3	0.00	9.3	0.013	0.22	0.22	0.22	0.48	5.0
**3e6i**	2.2	P 43	P 43 21 2	2	1	0.00	10.2	0.022	0.22	0.22	0.22	0.19	1.9
**2olk**	2.1	P 1 21 1	P 21 21 21	2	2	0.15	11.4	0.016	0.22	0.22	0.20	---	11.8
**2qhb**	2.4	P 32	P 32 2 1	2	1	0.00	12.6	0.024	0.23	0.21	0.21	---	5.0
**2r4j**	2.0	I 2 2 2	I 4 2 2	2	1	0.11	9.4	0.009	0.23	0.22	0.22	0.15	---
**1m9x**	1.7	P 1	P 1 21 1	2	4	0.11	12.0	0.016	0.23	0.23	0.20	0.09	11.9
**2zcz**	1.8	P 4	P 4 21 2	2	3	0.00	9.5	0.010	0.23	0.23	0.23	0.09	4.8
**3hrr**	1.9	P 21 21 21	P 43 21 2	2	1	0.03	7.8	0.006	0.23	0.23	0.23	0.09	---
**1diz**	2.5	P 31 2 1	P 61 2 2	2	1	0.00	9.1	0.010	0.23	0.23	0.23	0.43	---
**2cdq**	2.9	I 41	I 41 2 2	2	1	0.00	5.0	0.004	0.23	0.23	0.23	0.25	3.4
**2zh0**	2.5	P 3	P 63	2	6	0.00	9.3	0.011	0.23	0.23	0.22	0.55	3.1
**1dc4**	2.5	I 41	I 41 2 2	2	1	0.00	6.7	0.010	0.23	0.23	0.23	---	2.6
**1lnq**	3.3	P 61	P 61 2 2	2	4	0.00	1.8	0.001	0.23	0.22	0.18	0.90	5.2
**2hc0**	1.3	P 43	P 43 21 2	2	1	0.00	15.9	0.034	0.23	0.23	0.23	---	3.8
**3hlu**	2.6	P 21 21 21	P 43 21 2	2	1	0.18	6.1	0.008	0.23	0.23	0.23	0.31	4.5
**3i58**	2.7	P 65	P 65 2 2	2	1	0.00	4.1	0.005	0.23	0.23	0.23	0.26	---
**3ecy**	1.9	P 41	P 41 2 2	2	1	0.00	5.8	0.008	0.23	0.23	0.23	0.11	2.6
**1hpv**	1.9	P 61	P 61 2 2	2	1	0.00	9.6	0.027	0.23	0.23	0.23	---	---
**2iqq**	2.7	C 1 2 1	I 21 21 21	2	1	0.18	4.5	0.005	0.23	0.23	0.23	0.21	---
**3ex0**	1.3	P 1 21 1	C 2 2 21	2	1	0.34	12.6	0.017	0.23	0.11	0.11	0.03	13.1
**3evi**	2.7	P 31	P 31 2 1	2	1	0.00	5.9	0.007	0.24	0.23	0.23	0.32	9.4
**1asm**	2.4	P 1 21 1	C 2 2 21	2	1	0.14	9.0	0.014	0.24	0.23	0.23	---	8.7
**2o39**	2.9	P 3	P 3 2 1	2	2	0.00	6.2	0.005	0.24	0.24	0.23	---	4.4
**2c1n**	2.0	P 1 21 1	C 2 2 21	2	2	0.03	11.7	0.015	0.24	0.18	0.18	0.18	6.4

PDB Code	Resolution (Å)	Published Symmetry	Candidate Symmetry	No. of Cosets	Chains / asu	δ (°)	R_{symop} (I^{calc}) (%)	$\varphi(g_i)$	Δr_{sym} (Å)	Δr_{asu} (Å)	Δr_{chain} (Å)	Coord. Error (Å)	R_{symop} (I^{obs}) (%)
**1os2**	2.2	P 31	P 31 2 1	2	3	0.00	7.5	0.010	0.24	0.24	0.24	0.15	1.8
**2qar**	2.4	P 32	P 62	2	3	0.00	5.0	0.003	0.24	0.24	0.24	0.21	2.3
**1odx**	2.0	P 61	P 61 2 2	2	1	0.00	11.4	0.036	0.24	0.24	0.24	---	3.4
**2ggh**	2.2	P 4	P 4 21 2	2	2	0.00	8.4	0.009	0.24	0.24	0.24	0.13	3.3
**1zsf**	2.0	P 61	P 61 2 2	2	1	0.00	12.2	0.037	0.24	0.24	0.24	---	2.2
**3hrq**	1.8	P 21 21 21	P 43 21 2	2	1	0.01	9.0	0.007	0.24	0.24	0.24	---	---
**1kvo**	2.0	P 1 21 1	C 2 2 21	2	3	0.05	12.8	0.024	0.24	0.24	0.23	---	---
**3fla**	1.8	P 1 21 1	P 21 21 21	2	1	0.55	10.0	0.012	0.24	0.17	0.17	0.09	---
**2cl8**	2.8	P 32 2 1	P 62 2 2	2	1	0.00	6.2	0.007	0.24	0.24	0.24	0.23	2.9
**2h8n**	2.6	C 1 2 1	I 41 2 2	4	1	0.08	4.3	0.003	0.24	0.22	0.22	---	---
**3d1f**	2.0	P 32	P 32 2 1	2	2	0.00	9.7	0.015	0.24	0.24	0.23	0.06	1.6
**2qbx**	2.3	P 32	P 32 2 1	2	2	0.00	8.6	0.015	0.24	0.24	0.24	0.21	1.6
**2j0y**	2.4	P 32	P 32 2 1	2	3	0.00	8.4	0.009	0.24	0.24	0.23	0.20	3.7
**2c9b**	2.8	P 1	C 1 2 1	2	5	0.36	11.8	0.018	0.24	0.24	0.24	0.42	13.8
**1xao**	2.1	P 61	P 61 2 2	2	1	0.00	8.2	0.021	0.24	0.24	0.24	0.24	1.5
**1qbs**	1.8	P 61	P 61 2 2	2	1	0.00	13.8	0.051	0.24	0.24	0.24	---	---
**2tod**	2.0	P 1 21 1	P 21 21 21	2	2	0.58	7.1	0.007	0.25	0.21	0.18	---	---
**3gje**	2.3	P 43	P 43 2 2	2	2	0.00	8.0	0.010	0.25	0.24	0.24	0.21	1.3
**1tm0**	2.8	C 1 2 1	I 2 2 2	2	1	0.01	8.4	0.008	0.25	0.25	0.25	0.53	---
**1kx1**	2.8	P 1 21 1	C 2 2 21	2	3	0.01	8.1	0.013	0.25	0.25	0.24	0.60	3.2
**2b1g**	2.1	P 1	C 1 2 1	2	2	0.14	8.4	0.011	0.25	0.23	0.23	0.16	---
**1dmx**	2.5	P 1	C 1 2 1	2	1	0.09	12.2	0.026	0.25	0.24	0.24	---	---
**3dul**	1.8	C 1 2 1	I 2 2 2	2	1	0.01	13.0	0.020	0.25	0.25	0.25	---	---
**1a8g**	2.5	P 61	P 61 2 2	2	1	0.00	9.8	0.033	0.25	0.24	0.24	0.44	4.9
**2r45**	2.3	I 2 2 2	I 4 2 2	2	1	0.08	8.1	0.007	0.25	0.25	0.25	0.18	---
**2pdt**	2.2	P 1 21 1	P 21 21 2	2	2	0.02	6.8	0.007	0.25	0.21	0.16	---	3.2
**2c8u**	2.0	P 1 21 1	C 2 2 21	2	1	0.01	11.1	0.017	0.25	0.25	0.25	0.16	2.3
**1mer**	1.9	P 61	P 61 2 2	2	1	0.00	13.1	0.044	0.25	0.24	0.24	---	---
**1gnn**	2.3	P 61	P 61 2 2	2	1	0.00	11.6	0.040	0.25	0.24	0.24	---	---
**2r4n**	3.2	P 1 21 1	P 21 21 21	2	1	0.02	9.1	0.012	0.25	0.25	0.25	0.72	4.5
**3i0n**	2.3	P 1 21 1	C 2 2 21	2	1	0.04	7.9	0.008	0.25	0.25	0.25	0.19	---
**2j0h**	2.9	P 32	P 32 2 1	2	3	0.00	7.3	0.008	0.25	0.25	0.25	0.34	4.4
**2yrs**	2.3	P 1 21 1	P 21 21 21	2	4	0.05	8.9	0.010	0.25	0.25	0.25	0.17	5.5
**1met**	1.9	P 61	P 61 2 2	2	1	0.00	12.8	0.040	0.25	0.25	0.25	---	---
**1asn**	2.5	P 1 21 1	C 2 2 21	2	1	0.16	8.6	0.012	0.26	0.25	0.25	---	4.0
**3fa3**	2.6	P 32	P 62	2	8	0.00	9.4	0.013	0.26	0.25	0.25	0.28	8.3
**1jk6**	2.4	P 32	P 32 2 1	2	1	0.00	8.1	0.015	0.26	0.25	0.25	---	---
**1bwa**	1.9	P 61	P 61 2 2	2	1	0.00	12.6	0.041	0.26	0.24	0.24	---	---
**1hos**	2.3	P 61	P 61 2 2	2	1	0.00	13.0	0.063	0.26	0.25	0.25	---	---
**1upf**	2.3	P 1 21 1	C 2 2 21	2	2	0.00	9.4	0.022	0.26	0.26	0.25	---	---
**2d1n**	2.4	P 32	P 32 2 1	2	1	0.00	4.7	0.003	0.26	0.25	0.25	0.52	2.9
**2uwc**	2.3	P 6	P 6 2 2	2	1	0.00	5.2	0.005	0.26	0.26	0.26	0.10	3.9
**3ecn**	2.1	P 1 21 1	C 2 2 21	2	1	0.01	6.3	0.007	0.26	0.26	0.26	---	2.7
**2r46**	2.1	I 2 2 2	I 4 2 2	2	1	0.06	8.5	0.007	0.26	0.26	0.26	0.12	2.2
**2okr**	2.0	P 31	P 31 2 1	2	2	0.00	9.4	0.011	0.26	0.25	0.25	0.29	---

PDB Code	Resolution (Å)	Published Symmetry	Candidate Symmetry	No. of Cosets	Chains / asu	δ (°)	R_{symop} (I^{calc}) (%)	φ(g_i)	Δr_{sym} (Å)	Δr_{asu} (Å)	Δr_{chain} (Å)	Coord. Error (Å)	R_{symop} (I^{obs}) (%)
**1bd4**	2.2	P 1 21 1	C 2 2 21	2	2	0.18	8.7	0.019	0.26	0.26	0.25	---	---
**1g39**	1.2	P 1 21 1	P 21 21 2	2	2	0.04	8.1	0.008	0.26	0.25	0.25	0.20	2.3
**3e20**	3.5	P 43	P 43 21 2	2	4	0.00	2.8	0.003	0.26	0.26	0.25	0.34	2.3
**3d21**	2.2	P 61	P 61 2 2	2	1	0.00	6.6	0.017	0.26	0.25	0.25	0.20	0.9
**2frv**	2.5	P 1	P 65	6	2	0.23	10.7	0.011	0.26	0.06	0.05	0.27	---
**2fjm**	2.1	P 21 21 21	P 41 21 2	2	1	0.13	6.4	0.005	0.26	0.26	0.26	0.11	3.0
**1tvx**	1.8	P 1 21 1	C 2 2 21	2	2	0.04	9.8	0.012	0.26	0.26	0.26	---	5.8
**1aia**	2.2	P 1 21 1	C 2 2 21	2	1	0.00	9.4	0.015	0.26	0.26	0.26	---	---
**2ibx**	2.8	C 1 2 1	R 3 2 :H	3	2	0.15	6.7	0.007	0.26	0.26	0.26	0.32	5.2
**1umj**	1.6	P 3	P 63	2	1	0.00	11.6	0.014	0.26	0.26	0.26	0.14	1.6
**1td0**	1.9	P 3	P 63	2	2	0.00	4.6	0.002	0.26	0.26	0.26	0.35	2.3
**2j3u**	2.2	P 32	P 32 2 1	2	3	0.00	9.5	0.014	0.27	0.26	0.25	0.17	3.9
**1pl5**	2.5	P 65	P 65 2 2	2	1	0.00	5.1	0.006	0.27	0.25	0.25	---	1.8
**3c3f**	2.0	P 1 21 1	P 4 21 2	4	1	0.87	14.5	0.033	0.27	0.20	0.20	0.17	12.1
**2c7u**	2.4	P 1 21 1	P 21 21 2	2	3	0.01	9.4	0.013	0.27	0.27	0.26	0.33	3.6
**2goy**	2.7	P 1	C 2 2 21	4	2	0.16	8.3	0.009	0.27	0.14	0.13	0.58	---
**1hbv**	2.3	P 61	P 61 2 2	2	1	0.00	11.6	0.048	0.27	0.26	0.26	---	5.1
**1qbt**	2.1	P 61	P 61 2 2	2	1	0.00	14.5	0.057	0.27	0.26	0.26	---	---
**2v51**	2.4	P 1 21 1	P 21 21 2	2	2	0.06	7.0	0.009	0.27	0.27	0.26	0.19	4.3
**1arh**	2.3	P 1 21 1	C 2 2 21	2	1	0.00	11.1	0.016	0.27	0.27	0.27	---	---
**2bpe**	2.3	P 32 2 1	P 62 2 2	2	1	0.00	7.4	0.008	0.27	0.27	0.27	0.12	1.8
**1pwo**	2.6	C 1 2 1	I 21 21 21	2	2	0.05	8.5	0.011	0.27	0.27	0.27	0.44	3.9
**3jxe**	3.0	P 21 3	P 41 3 2	2	1	0.00	4.9	0.005	0.27	0.27	0.27	0.28	---
**1ahg**	2.5	P 1 21 1	C 2 2 21	2	1	0.01	11.7	0.021	0.27	0.26	0.26	---	---
**1fi8**	2.2	P 1 21 1	C 2 2 21	2	3	0.11	6.9	0.007	0.27	0.22	0.21	0.50	---
**1f5t**	3.0	P 41	P 41 21 2	2	2	0.00	6.0	0.008	0.27	0.26	0.26	0.76	5.8
**3cfk**	2.6	P 1	P 1 21 1	2	8	0.12	6.2	0.004	0.27	0.26	0.20	0.26	9.3
**3guo**	2.2	P 43	P 43 21 2	2	1	0.00	6.3	0.007	0.27	0.14	0.14	0.21	3.4
**2qnf**	3.0	P 31	P 31 2 1	2	1	0.00	3.4	0.004	0.27	0.26	0.26	0.80	3.2
**2hvb**	2.5	P 31	P 31 2 1	2	2	0.00	8.6	0.012	0.27	0.27	0.27	0.43	3.2
**1p7o**	2.3	I 41	I 41 2 2	2	3	0.00	9.4	0.013	0.27	0.27	0.27	0.30	1.2
**2a07**	1.9	P 1 21 1	C 2 2 21	2	3	0.11	8.3	0.009	0.27	0.27	0.27	---	2.2
**2ve4**	2.4	C 1 2 1	I 2 2 2	2	1	0.00	8.9	0.013	0.28	0.28	0.28	0.26	2.6
**1gnm**	2.3	P 61	P 61 2 2	2	1	0.00	11.7	0.048	0.28	0.27	0.27	---	---
**2fde**	2.7	P 61	P 61 2 2	2	1	0.00	10.2	0.030	0.28	0.27	0.27	---	2.2
**3fz3**	2.4	P 41	P 41 2 2	2	3	0.00	8.0	0.011	0.28	0.28	0.28	0.17	---
**1hvr**	1.8	P 61	P 61 2 2	2	1	0.00	10.8	0.032	0.28	0.27	0.27	---	---
**2jhh**	1.7	P 3	P 3 2 1	2	1	0.00	8.0	0.008	0.28	0.28	0.28	0.08	1.8
**1t77**	2.4	P 1 21 1	P 21 21 21	2	2	0.47	11.0	0.016	0.28	0.24	0.18	0.34	---
**2jiz**	2.3	P 1 21 1	P 21 21 21	2	7	0.19	7.9	0.008	0.28	0.16	0.09	0.14	12.7
**3ffu**	2.8	P 21 21 21	P 43 21 2	2	1	0.01	8.2	0.010	0.28	0.28	0.28	0.31	---
**1ari**	2.3	P 1 21 1	C 2 2 21	2	1	0.39	14.7	0.052	0.28	0.28	0.28	---	---
**3fhs**	2.7	P 61	P 61 2 2	2	1	0.00	7.6	0.010	0.28	0.28	0.28	0.18	1.2
**1vhb**	1.8	P 1 21 1	C 2 2 21	2	1	0.36	9.9	0.013	0.28	0.19	0.19	---	---
**2zgu**	2.4	P 32	P 32 2 1	2	1	0.00	10.7	0.029	0.28	0.28	0.28	0.47	1.9

PDB Code	Resolution (Å)	Published Symmetry	Candidate Symmetry	No. of Cosets	Chains / asu	δ (°)	R_{symop} (I^{calc}) (%)	$\varphi(g_i)$	Δr_{sym} (Å)	Δr_{asu} (Å)	Δr_{chain} (Å)	Coord. Error (Å)	R_{symop} (I^{obs}) (%)
**2p34**	2.1	P 32	P 32 2 1	2	2	0.00	8.8	0.012	0.28	0.28	0.28	0.14	2.3
**2w1i**	2.6	C 1 2 1	C 2 2 21	2	1	0.11	11.1	0.017	0.29	0.28	0.28	0.31	5.8
**2bb3**	2.3	C 2 2 21	P 41 21 2	2	1	0.15	11.5	0.018	0.29	0.29	0.29	0.28	2.2
**1bv7**	2.0	P 61	P 61 2 2	2	1	0.00	13.9	0.053	0.29	0.28	0.28	---	---
**3e6g**	2.8	P 41	P 41 21 2	2	2	0.00	6.9	0.010	0.29	0.29	0.29	0.32	1.7
**2j0g**	2.9	P 32	P 32 2 1	2	3	0.00	8.1	0.010	0.29	0.27	0.27	0.32	5.7
**2h1e**	2.2	I 41	I 41 2 2	2	1	0.00	11.4	0.016	0.29	0.29	0.29	0.14	3.9
**2glj**	3.2	P 1	P 1 21 1	2	12	0.13	5.1	0.004	0.29	0.29	0.27	0.74	---
**2nxq**	2.4	P 63	P 63 2 2	2	1	0.00	6.3	0.012	0.29	0.28	0.28	---	1.8
**1c6x**	2.5	P 21 21 21	P 43 21 2	2	1	0.00	11.8	0.020	0.29	0.29	0.29	---	---
**2pmc**	2.7	P 1	P 1 21 1	2	3	0.32	13.5	0.024	0.29	0.29	0.28	0.41	2.4
**1zoq**	2.4	P 61	P 61 2 2	2	2	0.00	4.6	0.005	0.30	0.29	0.29	---	2.8
**1htf**	2.2	P 61	P 61 2 2	2	1	0.00	11.5	0.034	0.30	0.29	0.29	---	---
**2c11**	2.9	P 43	P 43 21 2	2	2	0.00	7.6	0.012	0.30	0.14	0.11	---	4.3
**3ivu**	2.7	P 62	P 62 2 2	2	1	0.00	5.6	0.006	0.30	0.30	0.30	0.18	---
**3ex5**	1.8	P 1 21 1	C 2 2 21	2	1	0.26	12.4	0.019	0.30	0.12	0.12	---	---
**1gno**	2.3	P 61	P 61 2 2	2	1	0.00	11.5	0.049	0.30	0.30	0.30	---	---
**3dn9**	2.3	P 1	R 3 :H	3	2	0.55	10.2	0.015	0.30	0.16	0.09	0.24	19.5
**1asl**	2.6	P 1 21 1	C 2 2 21	2	1	0.07	8.6	0.015	0.30	0.30	0.30	---	8.2
**1aic**	2.4	P 1 21 1	C 2 2 21	2	1	0.02	11.4	0.020	0.30	0.30	0.30	---	---
**3buk**	2.6	R 3 :H	R 3 2 :H	2	2	0.00	7.7	0.010	0.30	0.30	0.30	---	2.8
**2gcj**	2.5	P 1 21 1	P 21 21 21	2	2	0.23	13.3	0.025	0.30	0.30	0.30	0.32	5.3
**2okv**	2.0	P 31	P 31 2 1	2	2	0.00	9.5	0.016	0.30	0.30	0.29	0.12	1.0
**2igy**	2.6	P 32 1 2	P 65 2 2	2	1	0.00	5.6	0.006	0.30	0.30	0.30	0.15	3.0
**1m9y**	1.9	P 1	P 1 21 1	2	4	0.26	14.3	0.023	0.30	0.30	0.26	0.11	17.4
**3ipj**	1.2	P 1 21 1	P 21 21 21	2	1	0.07	19.6	0.047	0.30	0.30	0.30	---	---
**1meu**	1.9	P 61	P 61 2 2	2	1	0.00	13.5	0.051	0.31	0.29	0.29	---	---
**1xqh**	1.8	P 1 21 1	P 21 21 2	2	2	0.04	9.6	0.162	0.31	0.30	0.30	0.11	3.5
**3bp9**	2.6	P 1	C 1 2 1	2	12	0.06	10.4	0.017	0.31	0.31	0.30	0.28	4.0
**2eis**	2.1	P 21 3	P 43 3 2	2	1	0.00	10.0	0.018	0.31	0.31	0.31	0.21	1.0
**2o94**	3.0	C 1 2 1	I 41 2 2	4	1	0.23	4.8	0.004	0.31	0.30	0.30	---	---
**1ws7**	1.9	P 61	P 61 2 2	2	2	0.00	7.4	0.010	0.31	0.31	0.30	0.10	1.5
**3dtj**	4.0	P 1	I 41	4	1	0.28	8.4	0.008	0.31	0.11	0.11	---	17.6
**1bv9**	2.0	P 61	P 61 2 2	2	1	0.00	13.2	0.044	0.31	0.31	0.31	---	---
**2fgk**	2.7	P 1 21 1	C 2 2 21	2	2	0.07	7.1	0.006	0.31	0.31	0.31	0.33	10.2
**3vhb**	2.1	P 1 21 1	C 2 2 21	2	1	0.08	8.8	0.015	0.31	0.30	0.30	---	---
**1ql4**	1.5	P 21 21 21	P 43 21 2	2	2	1.10	22.5	0.079	0.31	0.29	0.23	0.24	---
**1oax**	2.7	P 21 21 21	P 41 21 2	2	3	0.07	7.4	0.005	0.31	0.30	0.10	0.25	3.9
**1osi**	3.0	P 1 21 1	P 21 21 2	2	2	0.70	11.6	0.025	0.31	0.20	0.18	---	9.5
**1jss**	2.2	P 31	P 31 2 1	2	1	0.00	10.1	0.016	0.31	0.31	0.31	0.31	---
**1r19**	3.5	P 1	C 1 2 1	2	2	0.66	13.6	0.031	0.31	0.31	0.31	0.69	6.1
**3i5u**	2.6	P 65	P 65 2 2	2	1	0.00	4.0	0.004	0.31	0.31	0.31	0.62	---
**2ggg**	2.4	P 4	P 4 21 2	2	2	0.00	8.8	0.012	0.32	0.31	0.31	0.15	4.4
**1os4**	2.2	P 1	R 3 :H	3	4	0.95	14.9	0.023	0.32	0.31	0.29	0.44	14.0
**2iww**	2.7	P 21 21 21	P 43 21 2	2	1	0.62	6.2	0.004	0.32	0.31	0.31	0.25	---

PDB Code	Resolution (Å)	Published Symmetry	Candidate Symmetry	No. of Cosets	Chains / asu	δ (°)	R_{symop} (I^{calc}) (%)	φ(g_i)	Δr_{sym} (Å)	Δr_{asu} (Å)	Δr_{chain} (Å)	Coord. Error (Å)	R_{symop} (I^{obs}) (%)
**2vcv**	1.8	P 1	P 1 21 1	2	8	0.39	14.0	0.026	0.32	0.30	0.18	0.12	12.3
**1oay**	2.7	P 21 21 21	P 41 21 2	2	3	0.02	13.0	0.016	0.32	0.30	0.24	0.15	4.7
**1os9**	1.9	P 31	P 31 2 1	2	3	0.00	8.0	0.007	0.32	0.32	0.32	0.10	2.9
**1qbr**	1.8	P 61	P 61 2 2	2	1	0.00	16.0	0.068	0.32	0.32	0.32	---	---
**1sh9**	2.5	P 61	P 61 2 2	2	1	0.00	12.2	0.035	0.32	0.32	0.32	0.45	3.1
**1mui**	2.8	P 61	P 61 2 2	2	1	0.00	10.8	0.032	0.32	0.32	0.32	---	6.2
**1tqe**	2.7	P 1	C 1 2 1	2	3	0.04	6.9	0.006	0.32	0.32	0.27	0.82	2.0
**1qs1**	1.5	P 1	P 1 21 1	2	2	0.04	12.4	0.017	0.32	0.32	0.32	---	---
**2ef6**	2.1	P 32	P 32 2 1	2	2	0.00	9.3	0.013	0.32	0.32	0.32	0.14	3.2
**3b4v**	2.5	P 1	P 1 21 1	2	4	0.67	9.4	0.011	0.32	0.29	0.26	0.20	9.0
**3cjh**	2.6	P 1	C 1 2 1	2	6	0.36	10.8	0.011	0.32	0.31	0.29	0.34	9.1
**2p2k**	2.0	P 32	P 32 2 1	2	2	0.00	9.0	0.013	0.32	0.32	0.32	0.12	2.6

Table S2. Automated structure solution of 13 JCSG structures in all possible subgroups.

Refinement statistics presented in this Table suggest that the crystallographic *R*-factor is not especially sensitive to choice of symmetry, when the structure is refined in a subgroup of the published space group. Thirteen structures (randomly chosen cases representing 10 of the 13 Bravais types that have proper subgroups) were solved in all possible subgroup settings. Raw diffraction images from the JCSG were integrated and merged using symmetry constraints from the subgroup of interest, with resolution limits being determined separately for each subgroup during the data merging step. Free *R* flags were chosen randomly for each setting. Structures were not included in this Table if statistical tests (Padilla & Yeates, 2003; Zwart *et al.*, 2005) revealed the possibility of merohedral twinning. Subgroups with the same space group symbol and unit cell are not duplicate structure determinations, rather they represent different orientations of the symmetry axis with respect to the published cell. **C**, completeness of the dataset in the particular setting described, out to the indicated outer resolution limit.

PBD structure **1vr9**:

Space Group	Resolution (Å)	C (%)	*R*-work	*R*-free	*a* (Å)	*b* (Å)	*c* (Å)	α (°)	β (°)	γ (°)
C 1 2 1 (published)	1.70	80	0.247	0.270	228	53	44	90	100	90
P 1	2.01	62	0.245	0.274	44	53	117	77	80	90
C 1 2 1*	1.85	82	0.252	0.278	227	53	44	90	101	90
C 1 2 1	1.92	88	0.267	0.299	68	224	44	90	130	90
C 1 2 1	2.01	96	0.274	0.318	230	44	53	90	103	90
I 2 2 2	1.92	99	0.276	0.310	44	53	223	90	90	90

* Setting equivalent to that published. The structure can be solved in a higher-symmetry space group (*I* 2 2 2) than that published (*C* 1 2 1), albeit with a higher R-factor and an unfavorable Δr_{sym} of 0.58 Å.

PBD structure **1vrm**:

Space Group	Resolution (Å)	C (%)	*R*-work	*R*-free	*a* (Å)	*b* (Å)	*c* (Å)	α (°)	β (°)	γ (°)
P $2_1 2_1 2_1$ (published)	1.58	96	0.157	0.191	58	77	87	90	90	90
P 1	2.52	65	0.212	0.262	58	78	87	90	90	90
P 1 2_1 1	2.00	97	0.225	0.252	78	58	87	90	90	90
P 1 2_1 1	1.82	86	0.195	0.220	58	87	78	90	90	90
P 1 2_1 1	1.82	99	0.187	0.212	58	78	87	90	90	90
P $2_1 2_1 2_1$	1.75	100	0.201	0.227	58	78	87	90	90	90

PBD structure **1ztv**:

Space Group	Resolution (Å)	C (%)	*R*-work	*R*-free	*a* (Å)	*b* (Å)	*c* (Å)	α (°)	β (°)	γ (°)
$P\ 2_1\,2_1\,2_1$ (published)	3.10	100	0.193	0.224	92	92	119	90	90	90
P 1	3.50	53	0.160	0.242	92	92	120	90	90	90
$P\ 1\ 2_1\ 1$	3.43	71	0.185	0.245	92	120	92	90	90	90
$P\ 1\ 2_1\ 1$	3.23	88	0.178	0.237	92	92	120	90	90	90
$P\ 1\ 2_1\ 1$	3.23	93	0.172	0.242	92	92	120	90	90	90
$P\ 2_1\,2_1\,2_1$	3.06	100	0.186	0.244	92	92	120	90	90	90

PBD structure **1vky**:

Space Group	Resolution (Å)	C (%)	*R*-work	*R*-free	*a* (Å)	*b* (Å)	*c* (Å)	α (°)	β (°)	γ (°)
I 2 2 2 (published)	2.00	100	0.181	0.210	119	123	131	90	90	90
P 1	3.50	32	0.136	0.225	107	108	108	105	113	110
C 1 2 1	3.50	42	0.146	0.246	176	123	119	90	132	90
C 1 2 1	2.59	58	0.188	0.248	180	119	123	90	133	90
C 1 2 1	2.85	52	0.173	0.259	171	131	119	90	134	90
I 2 2 2	2.49	62	0.202	0.257	119	123	131	90	90	90

PBD structure **1z9f**:

Space Group	Resolution (Å)	C (%)	*R*-work	*R*-free	*a* (Å)	*b* (Å)	*c* (Å)	α (°)	β (°)	γ (°)
F 2 2 2 (published)	2.30	68	0.231	0.300	77	83	86	90	90	90
P 1	2.90	62	0.216	0.302	56	57	58	117	117	95
C 1 2 1	2.57	84	0.263	0.323	76	83	58	90	131	90
C 1 2 1	2.57	97	0.265	0.323	76	86	56	90	132	90
C 1 2 1	2.57	86	0.240	0.328	83	76	60	90	134	90
F 2 2 2	2.49	100	0.273	0.322	76	83	86	90	90	90

PBD structure **3b77**:

Space Group	Resolution (Å)	C (%)	*R*-work	*R*-free	*a* (Å)	*b* (Å)	*c* (Å)	α (°)	β (°)	γ (°)
P 4 (published)	2.42	100	0.216	0.254	151	151	76	90	90	90
P 1	3.50	57	0.159	0.225	76	151	151	90	90	90
P 1 2 1	3.03	95	0.212	0.255	151	77	151	90	90	90
P 4	2.83	100	0.204	0.243	151	151	76	90	90	90

PBD structure **1vrd**:

Space Group	Resolution (Å)	C (%)	*R*-work	*R*-free	*a* (Å)	*b* (Å)	*c* (Å)	α (°)	β (°)	γ (°)
I 4 (published)	2.18	94	0.219	0.258	120	120	144	90	90	90
P 1	3.05	59	0.209	0.247	112	112	112	99	115	115
C 1 2 1	2.62	87	0.219	0.258	170	144	120	90	135	90
I 4	2.48	96	0.217	0.246	120	120	144	90	90	90

PBD structure **1vjf**:

Space Group	Resolution (Å)	C (%)	*R*-work	*R*-free	*a* (Å)	*b* (Å)	*c* (Å)	α (°)	β (°)	γ (°)
P 4_3 2_1 2 (published)	1.62	99	0.146	0.167	51	51	121	90	90	90
P 1	1.85	70	0.171	0.221	51	51	121	90	90	90
P 1 2_1 1	1.74	99	0.175	0.206	51	51	121	90	90	90
C 1 2 1	1.79	75	0.173	0.213	72	72	121	90	90	90
P 1 2_1 1	1.69	98	0.178	0.202	51	51	121	90	90	90
P 1 2_1 1	1.74	79	0.172	0.201	51	121	51	90	90	90
P 2_1 2_1 2_1	1.65	99	0.178	0.215	81	51	121	90	90	90
C 1 2 1	1.74	79	0.171	0.229	72	72	121	90	90	90
C 2 2 2_1	1.69	81	0.178	0.207	72	72	121	90	90	90
P 4_3	1.65	99	0.176	0.207	51	51	121	90	90	90
P 4_3 2_1 2	1.65	100	0.183	0.219	51	51	121	90	90	90

PBD structure **1vr8**:

Space Group	Resolution (Å)	C (%)	*R*-work	*R*-free	*a* (Å)	*b* (Å)	*c* (Å)	α (°)	β (°)	γ (°)
P 3_2 2 1 (published)	1.75	93	0.150	0.182	58	58	88	90	90	120
P 1	2.17	78	0.202	0.238	58	58	88	90	90	60
C 1 2 1	2.04	89	0.197	0.248	101	58	88	90	90	90
C 1 2 1	2.04	96	0.208	0.246	101	58	88	90	90	90
C 1 2 1	2.04	99	0.202	0.256	101	58	88	90	90	90
P 3_2	1.94	100	0.200	0.237	58	58	88	90	90	120
P 3_2 2 1	1.99	100	0.204	0.245	58	58	88	90	90	120

PBD structure **2r6v**:

Space Group	Resolution (Å)	C (%)	*R*-work	*R*-free	*a* (Å)	*b* (Å)	*c* (Å)	α (°)	β (°)	γ (°)
$P\ 6_1\ 2\ 2$ (published)	1.25	85	0.128	0.154	46	46	268	90	90	120
$P\ 1$	2.03	58	0.192	0.221	46	46	268	90	90	90
$C\ 1\ 2\ 1$	1.61	82	0.201	0.221	46	80	268	90	90	90
$C\ 1\ 2\ 1$	1.71	72	0.193	0.209	80	46	268	90	120	90
$P\ 1\ 2_1\ 1$	1.84	90	0.197	0.236	46	268	46	90	90	90
$C\ 1\ 2\ 1$	1.61	56	0.194	0.226	46	80	268	90	90	90
$C\ 2\ 2\ 2_1$	1.71	91	0.201	0.241	46	80	268	90	90	90
$C\ 1\ 2\ 1$	1.61	71	0.194	0.230	80	46	268	90	90	90
$C\ 1\ 2\ 1$	1.61	82	0.203	0.239	46	80	268	90	90	90
$C\ 1\ 2\ 1$	1.61	83	0.197	0.227	80	46	269	90	90	90
$C\ 2\ 2\ 2_1$	1.61	84	0.194	0.216	46	80	268	90	90	90
$C\ 2\ 2\ 2_1$	1.53	70	0.199	0.228	46	80	268	90	90	90
$P\ 3_1$	1.53	86	0.195	0.225	46	46	268	90	90	120
$P\ 3_1\ 1\ 2$	1.61	90	0.200	0.227	46	46	268	90	90	120
$P\ 3_1\ 2\ 1$	1.46	83	0.201	0.239	46	46	268	90	90	120
$P\ 6_1$	1.53	87	0.198	0.218	46	46	268	90	90	120
$P\ 6_1\ 2\ 2$	1.53	88	0.208	0.242	46	46	268	90	90	120

PBD structure **1vlg**:

Space Group	Resolution (Å)	C (%)	*R*-work	*R*-free	*a* (Å)	*b* (Å)	*c* (Å)	α (°)	β (°)	γ (°)
R 3 2 :H (published)	2.00	100	0.180	0.218	176	176	355	90	90	120
P 1	2.62	61	0.196	0.248	156	156	156	69	69	69
C 1 2 1	2.29	80	0.205	0.237	258	176	156	90	116	90
C 1 2 1	2.29	79	0.202	0.252	258	176	156	90	116	90
C 1 2 1	2.21	98	0.203	0.246	258	176	156	90	116	90
R 3 :H	2.08	100	0.205	0.222	176	176	355	90	90	120
R 3 2 :H	2.14	100	0.204	0.219	176	176	355	90	90	120

PBD structure **1vmd**:

Space Group	Resolution (Å)	C (%)	*R*-work	*R*-free	*a* (Å)	*b* (Å)	*c* (Å)	α (°)	β (°)	γ (°)
I 2 3 (published)	2.06	98	0.170	0.219	130	130	130	90	90	90
P 1	3.50	35	0.142	0.260	113	113	113	109	110	109
C 1 2 1	3.50	49	0.154	0.214	184	131	130	90	135	90
C 1 2 1	3.50	44	0.143	0.243	184	130	130	90	135	90
R 3 :H	2.59	86	0.184	0.258	185	185	113	90	90	120
C 1 2 1	3.50	50	0.162	0.230	184	131	130	90	135	90
I 2 2 2	3.03	61	0.188	0.239	130	130	131	90	90	90
R 3 :H	2.59	92	0.199	0.248	184	184	113	90	90	120
R 3 :H	2.59	85	0.199	0.255	184	184	113	90	90	120
R 3 :H	2.59	86	0.196	0.254	184	184	113	90	90	120
I 2 3	2.33	100	0.194	0.239	130	130	130	90	90	90

PBD structure **1vlv**:

Space Group	Resolution (Å)	C (%)	*R*-work	*R*-free	*a* (Å)	*b* (Å)	*c* (Å)	α (°)	β (°)	γ (°)
F 4 3 2 (published)	2.25	99	0.190	0.215	210	210	210	90	90	90
P 1	2.75	98	N/A*							
C 1 2 1	2.75	100	0.193	0.300	258	149	149	90	125	90
C 1 2 1	2.75	99	0.181	0.267	258	149	149	90	125	90
C 1 2 1	2.75	100	0.177	0.274	210	210	149	90	135	90
C 1 2 1	2.75	100	0.191	0.261	210	210	149	90	135	90
C 1 2 1	2.75	100	0.184	0.287	210	210	149	90	135	90
F 2 2 2	2.60	100	0.190	0.236	210	210	211	90	90	90
I 2 2 2	2.67	100	0.196	0.263	149	149	211	90	90	90
I 4	2.53	100	0.188	0.285	149	149	211	90	90	90
I 4 2 2	2.60	100	0.196	0.252	149	149	211	90	90	90
C 1 2 1	2.75	100	0.190	0.260	258	149	149	90	125	90
C 1 2 1	2.75	100	0.184	0.241	258	149	149	90	125	90
C 1 2 1	2.75	100	0.182	0.233	258	149	149	90	125	90
C 1 2 1	2.75	100	0.188	0.258	258	149	149	90	125	90
I 2 2 2	2.67	100	0.181	0.238	149	149	210	90	90	90
I 2 2 2	2.60	100	0.192	0.241	149	149	211	90	90	90
I 4	2.53	100	0.184	0.254	149	149	210	90	90	90
I 4 2 2	2.67	100	0.192	0.256	149	150	210	90	90	90
I 4	2.53	100	0.184	0.222	149	149	211	90	90	90
I 4 2 2	2.60	100	0.190	0.265	149	149	211	90	90	90
R 3 2 :H	2.53	100	0.190	0.251	149	150	365	90	90	120
R 3 :H	2.67	100	0.189	0.283	149	149	365	90	90	120
R 3 2 :H	2.53	100	0.197	0.252	149	149	365	90	90	120
R 3 :H	2.67	100	0.191	0.250	149	149	365	90	90	120
R 3 2 :H	2.53	100	0.186	0.237	149	149	365	90	90	120
R 3 :H	2.67	100	0.176	0.265	149	149	365	90	90	120
R 3 2 :H	2.53	100	0.193	0.253	149	149	365	90	90	120
R 3 :H	2.67	100	0.194	0.260	149	149	365	90	90	120
F 2 3	2.53	100	0.192	0.250	211	211	211	90	90	90
F 4 3 2	2.53	100	0.196	0.265	211	211	211	90	90	90

* Refinement not performed.
